# An evolutionary driver of interspersed segmental duplications in primates

**DOI:** 10.1186/s13059-020-02074-4

**Published:** 2020-08-10

**Authors:** Stuart Cantsilieris, Susan M. Sunkin, Matthew E. Johnson, Fabio Anaclerio, John Huddleston, Carl Baker, Max L. Dougherty, Jason G. Underwood, Arvis Sulovari, PingHsun Hsieh, Yafei Mao, Claudia Rita Catacchio, Maika Malig, AnneMarie E. Welch, Melanie Sorensen, Katherine M. Munson, Weihong Jiang, Santhosh Girirajan, Mario Ventura, Bruce T. Lamb, Ronald A. Conlon, Evan E. Eichler

**Affiliations:** 1 Department of Genome Sciences, University of Washington School of Medicine, Seattle, WA 98195 USA; 2 Present Address: Centre for Eye Research Australia, Department of Surgery (Ophthalmology), University of Melbourne, Royal Victorian Eye and Ear Hospital, East Melbourne, VIC 3002 Australia; 3 Allen Institute for Brain Science, Seattle, WA USA; 4 Center for Spatial and Functional Genomics, Children’s Hospital of Philadelphia, Philadelphia, PA 19104 USA; 5 Department of Biology-Genetics, University of Bari, Bari, Italy; 6 Vaccine and Infectious Disease Division, Fred Hutchinson Cancer Research Center, Seattle, WA 98109 USA; 7 Molecular and Cellular Biology Program, University of Washington, Seattle, WA 98195 USA; 8 Pacific Biosciences (PacBio) of California, Incorporated, Menlo Park, CA 94025 USA; 9 Present Address: Department of Molecular and Cellular Biology, University of California, Davis, CA 95616 USA; 10 Present Address: Integrative Genetics and Genomics Graduate Group, University of California, Davis, CA 95616 USA; 11 Present Address: Brain and Mitochondrial Research, Murdoch Children’s Research Institute, Royal Children’s Hospital, Melbourne, VIC Australia; 12 Case Transgenic and Targeting Facility, Department of Genetics and Genome Sciences, School of Medicine, Case Western Reserve University, Cleveland, OH 44106 USA; 13 Department of Biochemistry and Molecular Biology, Department of Anthropology, Pennsylvania State University, University Park, PA 16802 USA; 14 Stark Neurosciences Research Institute, Indiana University School of Medicine, Indianapolis, IN 46202 USA; 15 Howard Hughes Medical Institute, University of Washington School of Medicine, 3720 15th Ave NE, S413C, Box 355065, Seattle, WA 98195-5065 USA

**Keywords:** Segmental duplication, Nuclear pore interacting protein, LCR16a, Gene fusion, Genomic instability

## Abstract

**Background:**

The complex interspersed pattern of segmental duplications in humans is responsible for rearrangements associated with neurodevelopmental disease, including the emergence of novel genes important in human brain evolution. We investigate the evolution of LCR16a, a putative driver of this phenomenon that encodes one of the most rapidly evolving human–ape gene families, nuclear pore interacting protein (*NPIP*).

**Results:**

Comparative analysis shows that LCR16a has independently expanded in five primate lineages over the last 35 million years of primate evolution. The expansions are associated with independent lineage-specific segmental duplications flanking LCR16a leading to the emergence of large interspersed duplication blocks at non-orthologous chromosomal locations in each primate lineage. The intron-exon structure of the NPIP gene family has changed dramatically throughout primate evolution with different branches showing characteristic gene models yet maintaining an open reading frame. In the African ape lineage, we detect signatures of positive selection that occurred after a transition to more ubiquitous expression among great ape tissues when compared to Old World and New World monkeys. Mouse transgenic experiments from baboon and human genomic loci confirm these expression differences and suggest that the broader ape expression pattern arose due to mutational changes that emerged in cis.

**Conclusions:**

LCR16a promotes serial interspersed duplications and creates hotspots of genomic instability that appear to be an ancient property of primate genomes. Dramatic changes to *NPIP* gene structure and altered tissue expression preceded major bouts of positive selection in the African ape lineage, suggestive of a gene undergoing strong adaptive evolution.

## Background

The human genome shows a complex pattern of highly identical, interspersed segmental duplications (SDs) [1, 2] as opposed to tandem and inverted SD clusters that predominate in most other mammalian lineages. This organization predisposes our species to large-scale rearrangements due to unequal crossing-over leading to genomic instability especially associated with neurodevelopmental delay and autism. Paradoxically, this susceptibility to copy number variation and disease appears to have been offset evolutionarily by the emergence of novel human-specific genes and transcripts [3] that have been associated with the expansion of the prefrontal cortex, extended neural neoteny, increased synaptic connectivity, and other potentially unique human adaptations [4–7]. The incomplete and interspersed nature of SDs has been key to their rapid innovation because the duplicate copies are often located within new genomic contexts. In most cases, they are found juxtaposing with other SDs of diverse evolutionary origin that carry different functional elements, creating potential for differential regulation and fusion of the duplicate genes [8]. The molecular basis for this modular organization among the primate lineage is largely unknown.

Human SDs are organized into an estimated 435 duplication blocks ranging in size from 50 kbp to multiple Mbp in length. Their ancestral reconstruction has revealed a highly nonrandom organization with respect to both chromosomal distribution and their structure. The 435 duplication blocks can be grouped into 24 distinct clades/groups, which are further organized around a set of 14 overrepresented “core” or seed duplicons [9]. The cores represent focal points for the expansion and duplicative transposition of SDs among primate genomes. Interestingly, core duplicons are transcriptionally active, encode gene families that are generally regarded as great ape specific or expanded, and often show signatures of positive selection. Emerging data suggest that cores have undergone independent and recurrent expansion in several primate lineages and, in some cases, demarcate at the breakpoints of large recurrent microdeletion/microduplication events associated with neurodevelopmental delay [10, 11].

Human chromosome 16 is particularly enriched in interspersed duplication blocks. In fact, approximately 10% of the euchromatic sequence of the short arm of chromosome 16 is composed of SDs referred to as LCR16 (low copy repeat on chr16) that evolved over the last 25 million years [12]. We previously identified a 20-kbp core duplicon (LCR16a) in association with almost all interspersed SDs along chr16p. Embedded within LCR16a is a gene family identified as nuclear pore complex interacting protein (*NPIP*) (aka *morpheus*). The *NPIP* gene family is remarkable because it demonstrates some of the most extreme examples of positive selection on record [13]. Moreover, most copies are interspersed (as opposed to clustered), and a comparative analysis of the human and orangutan genomes has shown that expansion has occurred independently in both lineages, including expansion to nonhomologous chromosomes [14]. The characterization of LCR16a among primates, however, has been hampered by its association with large duplicated regions, which are rarely assembled within most draft primate reference genomes.

In this study, we systematically investigate the organization of LCR16a more broadly across the primate phylogeny using hybridization of genomic libraries to discover and characterize genomic loci largely absent or collapsed within current reference assemblies. We find independent expansions of LCR16a to new chromosomal regions accompanied by the accumulation of flanking sequence, including in distantly related primate species, such as marmoset. Targeted transcript analysis in different species shows rapid turnover in gene structure with the loss and gain of entire exons and gene fusions specific to each lineage. Our results strongly support a model where LCR16a has independently driven the accumulation of interspersed primate SDs in conjunction with the evolution of a transcribed gene family undergoing strong adaptive evolution with an as-yet-unknown biological function.

## Results

### Primate survey of LCR16a copy and associated duplications

The expansion of LCR16a was originally regarded as specific to the ape lineage (based on comparisons to Old World monkey (OWM) where 1–2 copies have been identified and sequenced in baboon and macaque [12, 14]. Its presence in other non-ape genomes has been difficult to determine because associated duplications are typically large (100–500 kbp) and highly identical. As a result, they are typically collapsed or absent from the whole-genome assemblies. We therefore systematically screened for it by hybridization to primate BAC libraries from four species of New World monkey (NWM) (marmoset, dusky titi, owl monkey, and squirrel monkey) and one prosimian lineage (gray mouse lemur) for which a BAC library was available (see the “Methods” section). We initially estimated LCR16a copy number based on the number of positively hybridizing clones that were recovered (Table 1) followed by fluorescence in situ hybridization (FISH) and subsequent clone-insert sequencing. Interestingly, while most NWM and prosimian lineages had relatively low copy number estimates (< 4), one non-ape species, marmoset, stood out with 110 positively hybridizing clones suggesting an expansion of > 15 copies in that NWM lineage (average library coverage 6-7x).

**Table 1:** Estimation of LCR16a copy number in primate lineages

Primate lineage	Genomic library	Coverage	# clones	Copy number^^^
Gray mouse lemur	CHORI-257	7.7x	4	1
Dusky titi	LBNL-5	9.4x	12	< 2
Owl monkey	CHORI-258	5x	15	< 2
Squirrel monkey	CHORI-254	N/A	40	6
Marmoset	CHORI-259	N/A	110	18
Macaque	CHORI-250	5.5-6x	14	1*
Baboon	RPCI-41	5.2x	9	1*
Orangutan	CHORI-253	5-6x	127	20*
Gorilla	CHORI-255	6-7x	113	16*
Chimpanzee	RPCI4-43/CHOR-251	5-6x	212	37*
Human	RPCI-11	5-7x	–	17*

*Reported previously [14]

^^^Copy number estimated based on number of LCR16a BAC clones

**Fig. 1:**
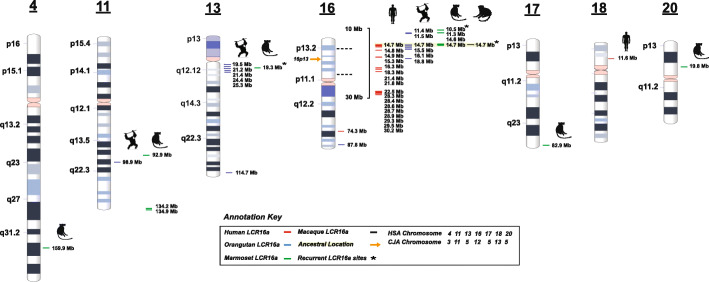
Distribution of LCR16a duplications in primate genomes. The location of LCR16a duplication blocks in marmoset (green dash), macaque (black dash), orangutan (blue dash), and human (red dash) are mapped against the GRCh38 human ideogram. The single-copy macaque locus maps to chr16p13, the ancestral origin from which all other copies were derived. Human LCR16a has expanded intrachromosomally across chromosome 16 predominantly on the short arm of chr16p. A more ancient copy of LCR16a, which is no longer expressed, locates to human chromosome 18. Marmoset and orangutan show expansions on other chromosomes including chr11 and chr13, respectively. Single-copy LCR16a duplication blocks are also mapped to chromosomes 20, 4, and 17/13 in the marmoset lineage

In order to reconstruct the evolutionary history of LCR16a, we selected BAC clones from the marmoset (CH259), squirrel monkey (CH254), and gray mouse lemur (CH257) clone libraries for complete insert sequencing using single-molecule, real-time (SMRT) sequencing (Additional file 2: Table S1) and performed comparative analysis against LCR16a-positive clones previously generated from other apes (*n* = 123) (Additional file 2: Table S2). In total, we generated ~ 11 Mbp of high-quality genomic sequence from 88 large-insert clones (Additional file 2: Table S1). We assembled large sequence contigs to traverse through SDs in order to anchor into unique sequence adjacent to each duplication (see the “Methods” section). We successfully and unambiguously mapped 11/12 marmoset-associated LCR16a copies to orthologous positions within the human reference genome assembly, GRCh38 (Fig. 1, Additional file 2: Table S3, Additional file 1: Fig. S1). Additionally, we mapped several loci from the gray mouse lemur and squirrel monkey corresponding to the ancestral location at chr16p13.1; however, in the latter case, we identified two additional LCR16a locations. The first corresponded to a shared site at 11q25 with marmoset; the second was a lineage-specific insertion on chr2q11.2. As expected, one marmoset LCR16a copy corresponded to the ancestral orthologous position on chromosome 16p13.1 (Additional file 2: Table S3) previously described as the origin of LCR16a sequence [14].

We assigned 11 LCR16a marmoset duplications to six distinct chromosomes (chr16, chr11, chr20, chr4, chr17q25/13q12.1; human phylogenetic group nomenclature) (Fig. 1). There are two striking properties of the marmoset loci. First, most of the marmoset LCR16a duplications are associated with other flanking duplicated sequences, often more than 100 kbp in length. We identify > 20 distinct LCR16a-associated duplicons in marmoset organized into duplication blocks ranging from 150.02–379.3 kbp (Table 2, Additional file 1: Fig. S1). For 12/23, the duplications appear lineage-specific with no evidence that these marmoset duplicons are duplicated in other ape lineages (see the “Methods” section, Additional file 2: Table S4). Of note, many of these secondary duplications map to genic regions (based on human RefSeq gene annotation), and we will refer to these duplicons henceforward based on their gene content. These results indicate that the expansion of LCR16a within the marmoset lineage has largely proceeded independently from that of other ape lineages. While LCR16a has clearly distributed to multiple chromosomes at different locations, more than half of the copies are clustered on either chromosome 11q or 16p where we observe three and five copies, respectively. Comparative analysis among the apes demonstrates that the p-arm of chr16 has been a particularly active for LCR16a duplication, while a 5-Mbp region adjacent to the telomeric region of chr13q12 has been a preferential target in the orangutan (Fig. 1).

**Table 2:** LCR16a-associated gene-containing segmental duplications

Lineage identified	HSA Location	Size (kbp)	Duplicon	Genes*	RefSeq gene
Primates	chr16:14711689-14726338	9	LCR16a	*NPIP*	Nuclear pore complex interacting protein
Marmoset/Orangutan	chr16:11527314-11548048	20.7	LCR16a-001	*LITAF*	Lipopolysaccharide induced TNF factor
Marmoset/Orangutan		24.0	LCR16a-001a	*RMI2*	RecQ mediated genome instability 2
Marmoset/Orangutan	chr16:11320235-11452411	132.8	LCR16a-002	*CTD-3088G3*	Pseudogene--predicted transcript
Primates	chr4:155451842-155466390	14.5	LCR16a-003	*MTRNR2L*	Pseudogene-predicted transcript
Primates	chr9:33565330-33577380	12	LCR16a-004	*ANKRD18B*	Ankyrin repeat domain 18B
				*REXO1*	RNA exonuclease 1 homolog
Marmoset	chr11:134239610-134264044	24.5	LCR16a-005	*VPS26B*	Retromer complex component B
				*THYN1*	Thymocyte nuclear protein 1
				*ACAD8*	Acyl-CoA dehydrogenase family member 8
Marmoset	chr11:93073398-93231243	157.8	LCR16a-007	*SLC36A4*	Solute carrier family 36 member 4
Primates	chr16:14965442-15044835	79.3	LCR16a-009	*PDXDC1*	Pyridoxal dependent decarboxylase domain containing 1
Primates	chr16:15387600-15416537	28.9	LCR16a-010	*MPV17L*	Mitochondrial inner membrane protein like
Marmoset/Gorilla	chr16:14681632-14616125	65.5	LCR16a-011	*PARN*	Poly(A)-specific ribonuclease
				*PLA2G10*	Phospholipase A2
				*BFAR*	Bifunctional apoptosis regulator
Primates	chr1:148979684-149033477	36.9	LCR16a-012	*PDE4DIP*	Phosphodiesterase 4D interacting protein
Marmoset	chr20:19864676-20010962	146.3	LCR16a-015	*RIN2*	Regulation of Rab5-mediated early endocytosis
Marmoset	chr20:20016850-20035552	18.7	LCR16a-016	*NAA20*	N (alpha)-acetyltransferase 20
				*CRNKL1*	Crooked neck pre-mRNA splicing factor 1
Marmoset	chr16:10666581-10697471	30.9	LCR16a-017	*TEKT5*	Tektin 5
Marmoset	chr3:150318404-150342624	24.2	LCR16a-018	*LINC01214*	Long intergenic non-protein-coding RNA 1214
Marmoset	chr2:132835355-132908086	72.7	LCR16a-019	*NCKAP5*	NCK associated protein 5
Marmoset	chr11:924494-962797	38.3	LCR16a-26	*AP2A2*	Adaptor related protein complex 2 subunit alpha 2
Marmoset	chr8:50623663-50639745	16.1	LCR16a-27	*SNTG1*	Syntrophin gamma 1
Primates	chr16:15062396-15097775	35.4	LCR16a-20	*RRN3*	RNA polymerase I transcription factor
African Ape/Prosimian	chr16:20411068-20501378	90.3	LCR16a-25	*ACSM2A, ACSM5*	Acyl-CoA synthetase medium-chain family member

*Most duplicate genes are incomplete, and annotation is based on RefSeq annotation of human reference genome (GRCh38)

### Sequence properties of LCR16a donors and acceptors

The availability of high-quality BAC sequence from the > 100 LCR16a primate loci allowed us to delineate the sequence composition of the flanking sequences that had been duplicated in association with LCR16a (termed donor sequences) and compare them to the genomic regions in which they had been integrated (termed acceptor regions). We identified 63 nonredundant “donor” duplicons compared to 27 non-overlapping acceptor regions and assessed enrichment for repeat content and GC composition by simulation (see the “Methods” section). The analyses showed that both donors and acceptor regions are significantly enriched for GC-rich and SINE (Alu) repeat content (Table 3, Additional file 1: Fig. S2). Because LCR16a associates with other flanking duplications, we analyzed seven integration sites (5 marmosets, 1 squirrel monkey, and 1 chimpanzee) corresponding to 14 duplication transition junctions in more detail (Table 4, Additional file 1: Fig. S3). We find that ~ 64% (9/14 boundaries) of new insertions have a SINE element (AluS) mapping precisely at the breakpoints (50 bp either side of the transition sequence) consistent with the threefold enrichment of Alu repeat elements reported previously for LCR16a junctions in other primates [14]. We also considered the sequence content of the locus prior to integration by examining the orthologous locus in outgroup primate species (e.g., human). Considering 13 LCR16a pre-integration loci across the primates, we find evidence of a loss of sequence at the pre-integration site ranging from 3.4 to 80.1 kbp in length for all except one case. The “deleted” sequence is particularly repeat-rich DNA (average 67.56% repeat content) (Table 4) showing the strongest enrichment for SINE elements (Table 3).

**Table 3:** Sequence composition analysis for donor/acceptor duplications and pre-integration sites

LCR16a type	Nonredundant sites	%GC (*E*, *p* value ± SE)	SINE (*E*, *p* value ± SE)	LINE (*E*, *p* value ± SE)
Donors	63	1.07, 1.6 × 10^−5^ ± 0.0005 *	1.47, 3.7 × 10^−8^ ± 2.4 × 10^−5^ *	1.03, 0.2 ± 0.05
Acceptors	27	1.11, 2 × 10^−9^ ± 6 × 10^−6^ *	1.68, 7.9 × 10^−6^ ± 0.00038 *	1.19, 0.009 ± 0.013
Pre-integration sites	13	1.18, 0.09 ± 0.08	1.66, 0.0077 ± 0.024	1.29, 0.024 ± 0.04

Note: we corrected for multiple hypothesis testing using FWER for a total of nine tests. The associations that had a corrected *p* value + SE ≤ 0.05 are denoted with an asterisk “*” 10,000 permutations. The “*E*” value represents the enrichment coefficient that was calculated based on the observed value divided by the expected, where the latter was defined as the mean of 10,000 genome-wide permutations. The retrotransposon statistics refer to the enrichment in LINE and SINE counts relative to the distributed segments

**Table 4:** Sequence composition of LCR16a sites of integration

Primate	Build	Breakpoint coordinate			Deletion at pre-integration site (kbp)	Repeats (%)	LTR (%)	LINE (%)	SINE (%)	Unique (%)	Duplication insertion (kbp)
Marmoset	hg38	chr4	160005326	160008761	3.4	99.6	.	81.3	17.6	0.03	21.6
Marmoset	hg38	chr20	20011993	20016575	4.6	66.8	4.9	19.3	37.5	33	25.7
Marmoset	hg38	chr11	134267373	134272307	4.9	48	10.3	5.8	26.6	52	243
Marmoset	hg38	chr11	93232994	93232995	0	.	.	.	.	.	148
Marmoset	hg38	chr16	11452411	11525004	72.6	57.6	3.66	14.7	29.3	42.3	21
Marmoset^^^	hg38	chr17_chr13	83228403	19349530	.	.	.	.	.	.	358.5
Chimpanzee	rheMac8	chr20	15098676	15131780	32.5	65.8	10.4	3.41	50	0.5	69.8
Squirrel monkey	hg38	chr2	100587592	100601735	14.1	69.9	2.6	4.8	35.3	30.1	52.5
Chimpanzee*	hg38	chr17	44694772	44700734	5.9	92.7	0	8.3	70.6	7.3	80
Orangutan*	hg38	chr13	24407831	24480143	80.1	55.6	5	23.1	22	44.4	140
Orangutan*	hg38	chr13	25361286	25366987	5.7	74.1	2.2	55.5	15.2	25.9	90
Gorilla*	hg38	chr16	27185611	27191436	5.8	83.7	30	26.3	22.8	16.3	100
Gorilla*	hg38	chr16	23303992	23307429	3.4	91.2	0	23.6	71.3	4.2	50
Chimpanzee*	hg38	chr16	2782469	2798105	16.1	61.8	17.3	13.4	23.7	38.2	30

Sequence composition of pre-integration site based on analysis of the orthologous location in the human genome (GRCh38)

*Reported previously [14]

^^^Cytogenetic rearrangement between chromosomes

### Recurrent sites of duplication and evolutionary chromosomal rearrangements

While the majority of LCR16a-associated marmoset duplications are independent, we identify three marmoset loci that overlap sites of LCR16a duplication among apes (Additional file 1: Fig. S4). For example, we sequence resolved a ~ 183-kbp duplication block in marmoset mapping to chr16p13.13. The duplication includes the carboxy terminus of *LITAF* and an additional noncoding RNA mapping adjacent to an LCR16a insertion (Additional file 1: Fig. S4A). A combined analysis of BAC sequencing and sequence read-depth profiles of the same region in the orangutan shows a larger ~ 220-kbp duplication block that also includes an independent duplication of *RASA3* from chromosome cytogenetic band 13q34 (chr13q34)*.* In both cases, the duplications map adjacent to LCR16a but the content and composition differ significantly, suggesting a preferential and recurrent site for LCR16a insertion (Additional file 1: Fig. S4A). Additionally, we also identified a recurrent site corresponding to chr13q12.1. In the human genome, this region harbors a 269.4-kbp duplication block containing the ~ 80-kbp duplicate gene *TPTE2*. This segment appears to have undergone multiple rounds of recurrent rearrangement with orangutan, gorilla, and human genomes showing the most extreme copy number among primates, albeit with differing breakpoints (Additional file 1: Fig. S4B). In the orangutan, there are two copies of LCR16a in association with this complex duplication block (Additional file 1: Fig. S4C). Notably, this is also a site of LCR16a duplication in marmoset (Additional file 1: Fig. S4D); however, multiple mapping signals to the GRCh38 human reference at chr13q12.1 and chr17q25 suggested this copy was part of a larger chromosomal rearrangement between marmoset and human.

**Fig. 2:**
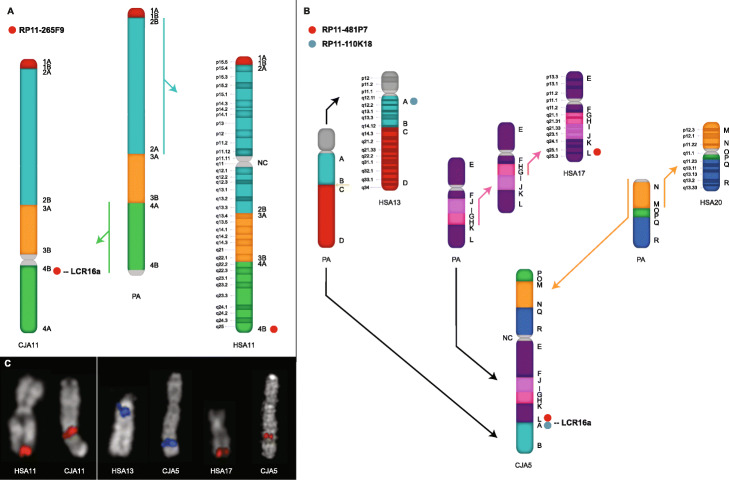
LCR16a-associated chromosomal evolutionary rearrangements in marmoset. **a** A chromosome ideogram schematic from marmoset chromosome 11 (CJA11) is compared to human and the predicted primate ancestor (PA). Synteny blocks are distinguished by colors and numbers while the position of the FISH probe is depicted by a red mark. The colored arrows indicate evolutionary inversions, and black arrows denote the ancestral orientation. A ~ 33 Mbp pericentromeric inversion in marmoset (green) is defined at the centromeric boundary by an LCR16a-associated duplication block. Both the predicted primate ancestor and human (chr11q22.2-q25) are shown to be in direct orientation based on the order of the blocks analyzed in other primate lineages [15]. **b** A complex chromosomal rearrangement on marmoset chromosome 5 (CJA5) is identified between the ancestral chromosomes of HSA17 and HSA13; again LCR16a defines the boundary of this event. Note that the evolutionary order of the two inversions that led to HSA17 is unknown and the sequence shown in the figure is only one of two possibilities. **c** Single-color FISH analysis using metaphase spreads is used to confirm the presence of chromosomal rearrangements between marmoset and human. A probe mapping to the telomeric region of HSA11 (RP11-265F9) shows a signal mapping to a syntenic region at the CJA11 centromere. At CJA5, two adjacent FISH signals from (RP11-481P7 and RP11-110 K18) map to the ancestral telomeric region of HSA17 and the centromeric region of HSA13. CJA, *Callithrix jacchus*; HSA, *Homo sapiens*; PA, primate ancestor; NC, neocentromere

To confirm that this was indeed not a reference assembly artifact, we performed a series of single-color metaphase FISH experiments in lymphoblastoid cell lines corresponding to marmoset and human (see the “Methods” section) (Fig. 2). Using two probes mapping to orthologous regions on human chr17q25 and chr13p11, we detected a single FISH signal mapping to the q-arm of CJA5 (132–133 Mbp) (Fig. 2c). This region corresponds precisely to a marmoset-specific ~ 380-kbp LCR16a-associated duplication block. Cytogenetic analysis confirms that region defines the boundary of a complex set of fusion events that led to the formation of marmoset chromosome 5 (CJA) (Fig. 2b) [16]. Of note, we also identified a large pericentromeric inversion mapping to human chromosome 11 (11q22.2-11q25) (Fig. 2a). FISH analysis confirms that this inversion spans almost ~ 33 Mbp and occurred specifically in the marmoset lineage (Fig. 2c) [15]. Again, LCR16a defines the boundary of this marmoset-specific event.

### Phylogenetic reconstruction

**Fig. 3:**
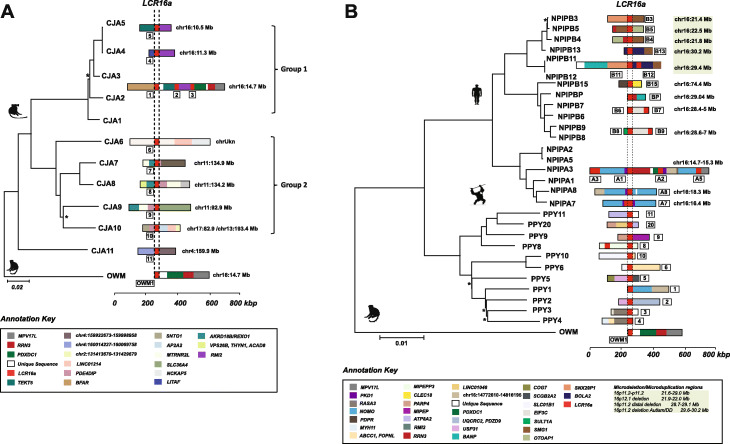
Phylogeny and sequence composition of LCR16a duplication blocks. **a** Phylogenetic analysis of LCR16a copies in the marmoset lineage. The size and complexity of the mosaic LCR16a duplications are depicted by colored duplication blocks adjacent to each node (refer to Additional file 2: Table S4 for individual duplicon map locations). Map locations for the duplication blocks are depicted against the GRCh38 reference assembly. The LCR16a core element is shown as dashed lines. Nodes with < 90% bootstrap support are indicated by stars. Phylogenetic analysis reveals two distinct clades in marmoset, one mapping to chromosome 16 (group 1), the other mapping to chromosome 11 (group 2). Duplications with similar block architectures cluster together and the two clades suggest multiple independent events in marmoset. **b** Phylogenetic sites of recurrent LCR16a duplication in the orangutan and human lineages. The duplication blocks are numbered according to genomic location of a locus in the chromosome, and block coordinates correspond to the GRCh38 reference assembly. Phylogenetic analysis predicts two distinct clades depicting the independent origins of human and orangutan LCR16a duplications. Regions of recurrent microdeletion/microduplication associated with intellectual disability and autism in humans are highlighted in gray. Nodes with < 90% bootstrap support are indicated by stars

In order to assess lineage specificity of the duplications, we constructed a phylogenetic tree using the 12 marmoset LCR16a copies and a single-copy orthologous region in macaque as an outgroup (Fig. 3a). This analysis reveals two distinct clades with strong bootstrap support in marmoset: one corresponding to the dispersal on chromosome 16 (group 1) and the other corresponding to the expansion on chromosome 11 as well as other chromosomes (group 2). As expected, the duplication architecture within groups is generally more similar than between groups as reflected by the bifurcated topology (Fig. 3a). Using a divergence time of 35 million years ago (mya) for separation from the OWM lineage [17], we estimate LCR16a initially duplicated ~ 25 mya within the NWM lineage seeding relatively few copies with most of the expansion occurring later between 5 and 13 mya (Additional file 1: Fig. S5). This relatively recent dispersal of LCR16a is consistent with almost all of the copies being lineage-specific or recurrent within marmoset when compared to other NWM species. While we cannot completely rule out the effects of interlocus gene conversion, the fact that 10/11 marmoset loci map to non-orthologous locations when compared to other primates is consistent with a more recent dispersal of LCR16a in this lineage. In addition, it should be noted that OWM species, in general, have a single copy of LCR16 mapping to the same ancestral location [13]. In this context, all duplicate copies are orthologous to this ancestral locus irrespective of lineage-specific expansions or subsequent gene conversion events, which may confound the topology and timing estimates of the terminal branches.

We repeated the analysis using 86 draft and fully sequenced LCR16a loci from humans and nonhuman primates (NHPs) (Fig. 3b and Additional file 1: Fig. S6). The tree topology among the great ape lineage indicates four waves of LCR16a expansion. First, there was a clear independent expansion of LCR16a between Asian (23 loci) and African ape lineages (63 loci). The architecture of the LCR16a duplications, however, is similar. In both lineages, these expansions occur in conjunction with the acquisition of lineage-specific duplicons occurring at the flanks and with map locations that are with few exceptions non-orthologous between African and Asian apes. Among the African ape lineage, we further identify three additional clades corresponding to expansion of the *NPIPA* isoform and two further expansions of *NPIPB*. Among the apes, there are both orthologous and non-orthologous copies in each lineage suggesting these duplications occurred before and after speciation. A detailed analysis from a subset of chimpanzee and gorilla loci (*n* = 15), for example, showed that only 40% of LCR16a copies were orthologous to locations identified in human (1/3 gorilla and 5/12 chimpanzees).

### Recurrent evolutionary restructuring of the chr16p13.1 ancestral locus

**Fig. 4:**
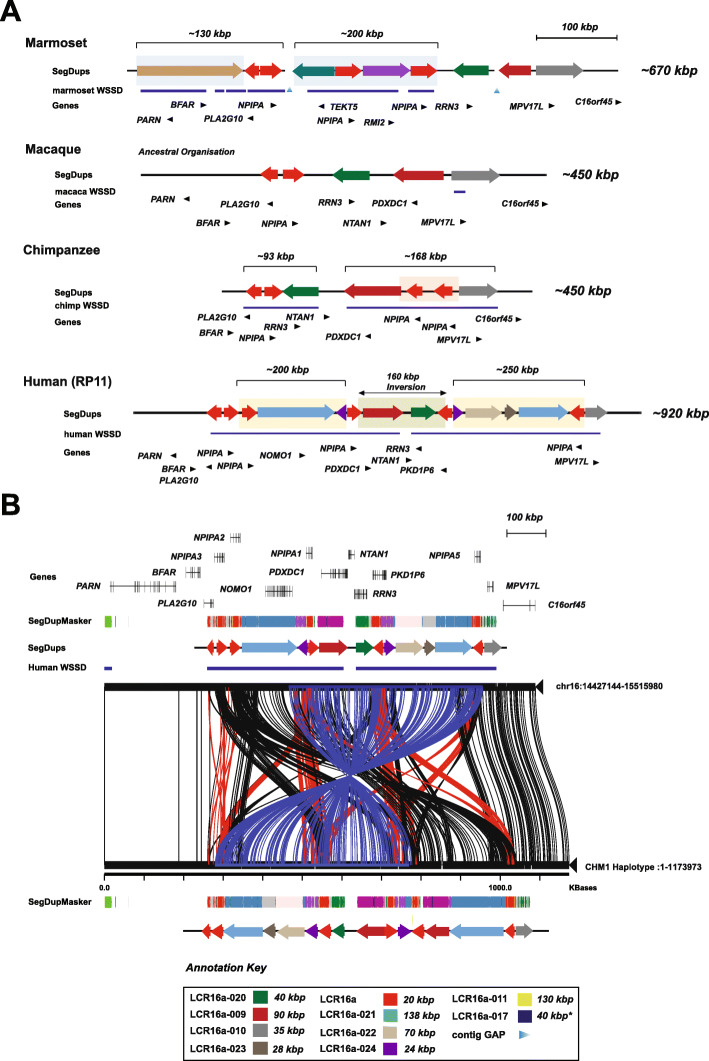
Structure of the ancestral chromosome 16p13 locus. The structure and organization of chr16p13 in four primate lineages is shown based on sequencing of a tiling path of BAC clones for each primate haplotype. SDs (colored arrows) and gene models (black arrows) are shown with respect to lineage-specific duplications (blue bars) identified based on sequence read-depth (WSSD) [1]. **a** The chromosome 16p13 region has expanded and contracted hundreds of kilobases due to lineage-specific duplication. Note the ancestral ~ 160-kbp inversion between the human RP11 haplotype and all other primates. The ancestral LCR16a duplicon in macaque shows a single copy of *NPIP*, compared to three copies in marmoset and chimpanzee, and five copies in human. **b** A Miropeats comparison between two human haplotypes at the ancestral locus on chr16p13. CHM1 BACs tiling across the chr16p13 region were sequenced and assembled using PacBio SMRT sequencing to create a super contig. The SD organization is depicted using colored arrows. Miropeats between RP11 and CHM1 contigs shows pairwise differences between orthologous regions. A ~ 400-kbp inversion is detected in the CHM1 haplotype, flanked by LCR16a core duplicons (blue lines). CHM1 also carries an additional duplication corresponding to LCR16a-009, which contains *PDXDC1* (maroon arrow) and incomplete duplication of LCR16a-021 *NOMO1* (blue arrow)

We previously mapped the ancestral location of LCR16a to chr16p13.1 in macaque and baboon, both confirmed to represent only a single copy of LCR16a [13, 14]. In order to gain insight into the structural diversity of this region throughout primate evolution, we sequenced an additional 28 large-insert clones across four primate lineages targeted to the ancestral chr16p13.1 locus (Additional file 2: Table S3). We sequenced and assembled contiguous haplotypes in macaque and chimpanzee, as well as an additional human haplotype CHM1 generating > 2 Mbp of high-quality finished sequence (Fig. 4). Our analysis suggests that the 16p13.1 locus has been subject to multiple rounds of recurrent rearrangement where each lineage differs structurally with respect to gene and duplication content.

The macaque and chimpanzee haplotypes, for example, show the simplest organization containing four ancestral duplicons, which include the genes *MPV17L*, *PDXDC1*, *RRN3*, and *NPIPA* (green, maroon, gray, and red arrows, respectively). These duplications are shared among all the lineages and likely represent the primate archetype (Fig. 4a). In chimpanzee, we identify two additional inverted copies of LCR16a, which are absent from the macaque assembly. These chimpanzee-specific LCR16a copies reside in a large ~ 168-kbp region of increased read depth (detected by whole-genome shotgun sequence detection [WSSD]; blue underlining bar) and add an additional 40 kbp of sequence to the locus (red shading) (Fig. 4a). The human locus is particularly derived, and we identify numerous additional structural changes between it and other NHPs. For example, a ~ 160-kbp inversion containing three genes (*PDXDC1*, *RRN3*, and *NTAN1*) is present in all NHPs relative to the human reference assembly*.* The inversion is flanked by LCR16a repeats mapping in inverted orientation. Mouse synteny analysis confirms that the NHP inversion is likely the ancestral state. Compared to human, chimpanzee lacks at least eight individual duplicons totaling > 505 kbp of sequence. These duplicons are part of two larger cassettes of ~ 200 and ~ 250 kbp, which are flanked by LCR16a duplications. Sequence analysis of an alternative human haplotype (CHM1) reveals a larger ~ 450-kbp inversion spanning inverted copies of LCR16a (*NPIPA1* to *NPIPA5*) (Fig. 4b). We used Strand-seq data to infer the frequency of the inversion and found it segregated in approximately 79% of European individuals [18] consistent with a potential large-scale inversion polymorphism in the human population. In addition to the inversion, the CHM1 haplotype differs structurally from the GRCh38 reference assembly by the presence of two additional duplications, including a complete duplication of LCR16a-024 (*PDXDC1*) and incomplete duplication of LCR16a-021 (*NOMO1*).

In marmoset, our analysis of the ancestral region shows an independent pattern of SD. We estimate ~ 330 kbp of duplication; however, the region has been completely restructured when compared to the human assembly. For example, we identify a ~ 130-kbp marmoset duplication containing *BFAR*, *PLA2G10*, and the first 15 exons from *PARN* (Fig. 4a)*.* Comparative sequence analysis reveals that only *PLA2G10* is duplicated in chimpanzee and human, while this entire 130-kbp block has undergone an independent duplication in gorilla, albeit with differing breakpoints (Additional file 1: Fig. S7). Finally, we also identified a ~ 200-kbp segment composed of two incomplete duplications of *TEKT5* and *RMI2*. These duplications appear to be specific to the marmoset lineage and contain three copies of LCR16a mapping, once again, at the boundaries of the events.

### Transcript characterization, diversity, and positive selection

**Fig. 5:**
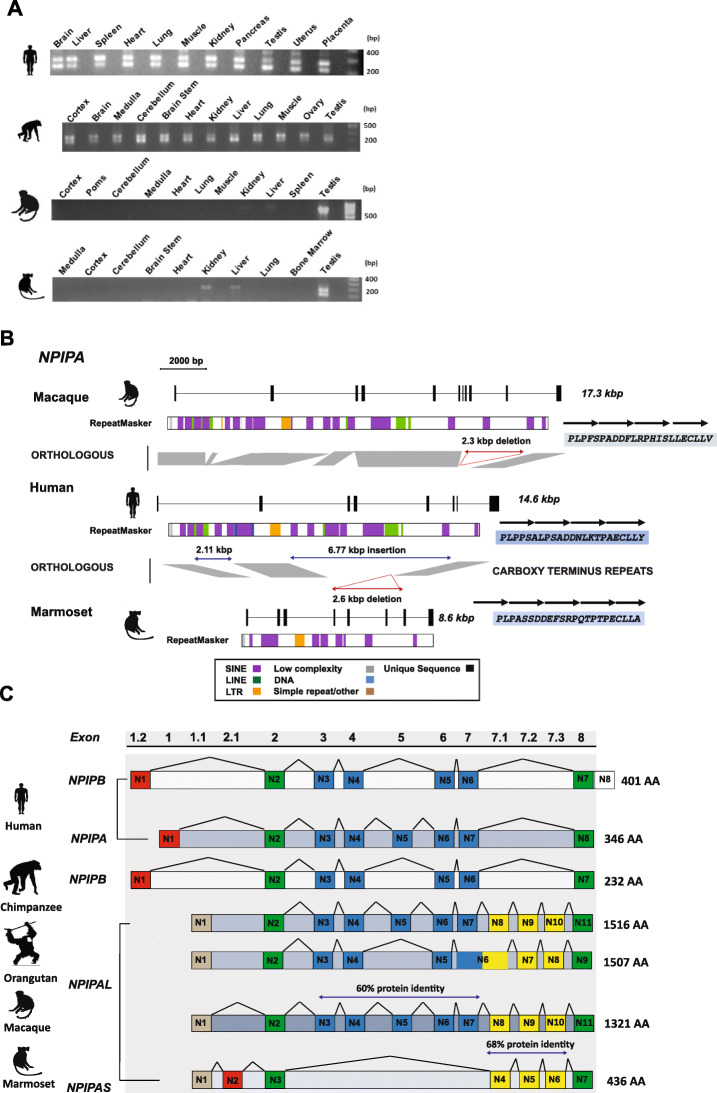
*Continued*

Among humans and chimpanzees, LCR16a is remarkable in that it encodes a gene family, *NPIP*, which demonstrates some of the strongest signatures of positive selection based on an excess of amino acid replacement changes [13]. The canonical gene structure for the *NPIP* family in humans consists of eight exons with a variable amino acid repeat motif located at the carboxy terminus. Based on this structure and genome sequence from different primate lineages, we initially investigated patterns of *NPIP* expression in a diversity panel of tissues/subtissues (see the “Methods” section for complete list) originating from human and NHP primary source material (Additional file 1: Fig. S8). We designed a series of specific and degenerate RT-PCR assays based on RNA generated from marmoset testis source tissue (Additional file 2: Table S7). We observe that *NPIP* is most highly expressed in the testis for both NWM and OWM lineages, with weaker expression in the liver (NWM), thymus, and kidney (OWM) (Fig. 5a and Additional file 1: Fig. S8). In stark contrast, the pattern of expression among humans and nonhuman great apes appears much more ubiquitous. This suggests that the regulatory machinery required for ubiquitous expression may have been acquired in *cis* possibly by the juxtaposition of SDs adjacent to LCR16a during great ape evolution.

In order to generate more complete gene models, we focused on capturing the predominant full-length cDNA sequences from various primates followed by long-read SMRT sequencing (see the “Methods” section) and mapping transcripts against the primate genomic loci (Fig. 5b). Comparison of the NHP and canonical human gene models reveals several major structural differences. First, the human and macaque loci are almost twofold larger than marmoset due to an accumulation of Alu repeats within the introns leading to expansion and restructuring of the intronic regions in both macaque and apes (Fig. 5b). With the exception of the African ape lineage, all NHPs carry an additional three spliced exons (referred to as ex.7.1–7.3) (Fig. 5c). These three exons map to a 2.73-kbp deletion in intron 7 that removes these ancestral exons from all human copies. We map the corresponding breakpoint in > 125 large-insert clones representing nine primate lineages. The deletion is present in all LCR16a copies associated with African apes, but absent in all but one orangutan copy, suggesting the deletion and loss of these three exons occurred in the great ape ancestor and subsequently became fixed among African apes. Finally, we identify ~ 8.7-kbp of intronic sequence in human *NPIPA* that is not orthologous to the marmoset gene model. This includes a 6.77-kbp region containing introns 2–6 and an additional five transcribed exons. This region in human is notable because it contains tandem arrays of anti-sense Alu/SINE elements that flank or are adjacent to these five exons.

Overall, our analysis reveals four canonical subtypes of the *NPIP* family predicted to encode proteins that vary radically in amino acid composition and length: *NPIPA*, *NPIPA-S*, *NPIPA-L*, and *NPIP-B* (Additional file 1: Fig. S9). Among marmoset, the predominant gene model is *NPIPA-S* (NPIP type A-short) consisting of seven exons, encoding a 436 AA open reading frame (ORF) with a predicted molecular weight of 47.9 kDa. *NPIPA-L* (NPIP A-long) is the most abundant among primate species present in the OWMs (macaque and baboon) and orangutan. It is also the largest, containing 9–11 exons and encoding a predicted protein > 135 kDa. A major difference between these two subtypes is the presence of a short exon anchored in a DNA/MER30 element present in all *NPIPA-S* members and the addition of four constitutive exons to *NPIPA-L* observed in both macaque and orangutan transcripts (exons N3–7). *NPIP-Type A*, as described previously [13], represents the African ape archetype. The evolution of this subtype is characterized by major restructuring at both the N- and C-termini when compared to *NPIPA-L*, which includes the acquisition of a novel promoter/start initiation codon (~ 700 bp upstream) and the loss of three exons in the C-terminal region of the peptide (ex7.1–7.3) described above. Cloning and subsequent sequencing of the fourth subtype *NPIPB* [bioRxiv 10.1101/116087] in humans reveals an alternate promoter and translation initiation, the complete absence of exon 5, a 17 amino acid expansion of exon 4, and an Alu insertion in exon 8. This insertion and subsequent frameshift creates an entirely new amino acid repeat motif specific to all members of the *NPIPB* subtype identified in human and correlates with two independent expansions and positive selection in the human lineage over the last two million years (Additional file 1: Fig. S6 and S10, Additional file 2: Table S5).

One feature of the predicted protein structure of the *NPIP* family is the variable number of tandem amino acid repeats that define the carboxy terminus and distinguish paralogous copies (Fig. 5d). Once again, this protein-encoding VNTR shows lineage-specific signatures. In gorilla for example, we find that the *NPIP* ORF is expanded by several kilobase pairs and shows extensive copy number diversity when compared to marmoset, where the repeat structure appears to be far more stable among the paralogs. The composition of the individual amino acid repeat units also varies substantially for different species (Fig. 5e). OWMs and NWMs, for example, contain only an *NPIPA*-associated repeat unit consisting of 23 AA. This repeat contains a characteristic *PLPPS* motif at the beginning of the repeat. By comparison, all orangutan copies contain a larger 69 AA cassette, consisting of two 23 AA *NPIPA* units and a final divergent repeat (23AA) created through a 24-bp frameshift insertion. Similarly, we identify 6/21 chimpanzee copies that contain a highly repetitive tandemization of four AA (PPSP and PPSA), which are interspersed throughout the larger cassettes consisting of repeat units ranging between 14 and 25 AA (Fig. 5e).

We previously reported evidence of strong positive selection for two coding exons, based on an excess of nonsynonymous amino acid replacements in the African ape lineages [13]. We revisited this positive selection analysis in light of our broader survey of gene structure among additional species. We performed maximum likelihood analysis and hypothesis tests using the dN/dS (nonsynonymous/synonymous) (ω) ratio implemented in PAML [19] based on the entire gene model from representative mammals (dog), OWM (macaque and baboon), and apes (orangutan and human) (Additional file 1: Fig. S11-S12) (see the “Methods” section). In the dog, we found that there was no significant difference between the null model under neutrality and test for positive selection (*p* = 0.2254, LRT d.f. = 1) indicating that the dog *NPIP* copies likely evolved neutrally. In comparison, the model under selection within the primate lineage was a significantly better fit to the data than the neutral model (*p* = 2.6867e-5, LRT, d.f. = 1). Branch tests specifically comparing the African ape clades and other NHPs confirm that the signal is driven by copies of *NPIP* confined to the African ape lineage (*p* = 6.5975e-5, LRT, d.f. = 1), and this effect is largely driven by members of the *NPIPB* subfamily, which emerged and expanded in chimpanzees, humans, and gorillas (Additional file 1: Fig. S18-S19). These observations are consistent with dN/dS ratio estimates performed at the level of single exons, where we identify exons 2, 4, and 6 showing a significant excess of amino acid replacements only in African apes when compared to the OWM lineage (Additional file 2: Table S5, Additional file 1: Fig. S10). While we also detect some evidence of elevated dN/dS in other ape lineages, for example, exon 7.3 in the orangutan lineage (Additional file 2: Table S5), we find that this does not reach statistical significance using a likelihood ratio test (*p* = 0.087). These results combined with our earlier work [13] support a major burst of positive selection specifically in the African ape lineage, while other NHPs (OWM or NWM lineages) and mammals (canids) show patterns of amino acid replacement indistinguishable from neutral evolution (Additional file 2: Table S5, Additional file 1: Fig. S11-S12 and S18-S19).

### Locus-specific expression and duplication dispersal

Because of the deeper evolutionary age and greater diversity among the 12 marmoset loci, it is possible to assign full-length cDNA transcripts to specific loci based on diagnostic paralogous sequence variants allowing us to distinguish actively transcribed loci. Our analysis finds that all *NPIP* transcripts in this species originate solely from chromosomes 11 and 16—chromosomes that experienced independent intrachromosomal expansions (Fig. 3a). Notably, four marmoset LCR16a copies appear transcriptionally silent (chr4, chr20, chrUkn, and chr17q25-13q). These loci correspond to chromosomes that harbor solo copies, without evidence of subsequent intrachromosomal expansions. The situation is analogous to the dispersal of LCR16a among the great apes. In the ape lineages, the majority of transcripts originate from chromosome 16 (African apes) or chromosome 13 (orangutans) where the duplications have spread by successive rounds of intrachromosomal duplication.

A common feature of all LCR16a duplications in the different primate lineages is that they are associated with other lineage-specific SDs on their flanks [14]. Because the SDs are often gene rich, this juxtaposition creates a tremendous potential for transcript and gene fusions. In marmoset, almost all transcripts originating from chromosome 11 are fusion transcripts with flanking SDs, while the chromosome 16 transcripts maintain an ORF consistent with the *NPIPA-S* ancestral gene model. At chromosome 11q25 in marmoset, for example, we identify a 252 AA fusion transcript that originates from an adjacent 1q21.1 duplication containing *PDE4DIP* and LCR16a (Additional file 1: Fig. S13). This gene fusion maintains 80% and 77% protein homology with the corresponding genic segments from *PDE4DIP* and *NPIPA-S*, respectively. Similar transcript fusions have been documented in orangutan, chimpanzee, and gorilla such as the *ABCC1-NPIP* fusion transcript spanning a 195-kbp gorilla-specific duplication (Additional file 1: Fig. S13). In humans, one of the most abundantly expressed sequence tags that associate with *NPIP* spans a *PKD1/NPIP* fusion transcript on chr16p13.1. Although these fusion transcripts seldom maintain an ORF, they are often species-specific because most involve lineage-specific duplications.

### BAC transgenic model of expression

**Fig. 6:**
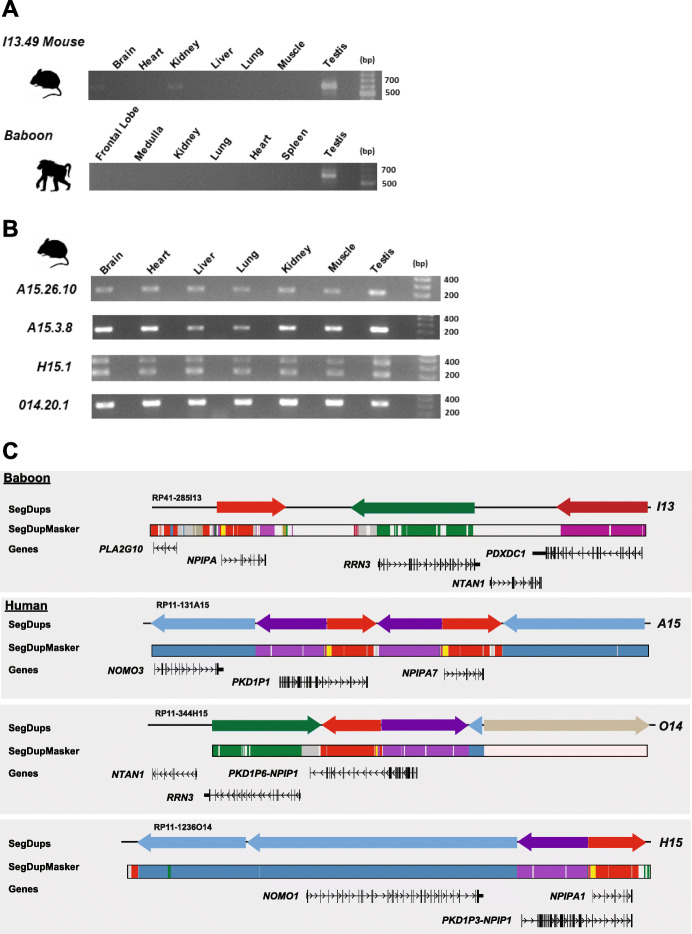
Comparison of mouse BAC transgenic and *NPIP* expression. **a** RT-PCR of cDNA obtained from a panel of mouse transgenic tissues. Mouse lines (I13.49) derived from baboon (RPCI41-285I13) BAC integration demonstrate robust expression in the testis. This is a single copy of *NPIP* and orthologous to the ancestral location. **b** RT-PCR of cDNA generated from a panel of mouse transgenic tissues carrying human BAC clones with different *NPIP* paralogs. Mouse transgenic A15.3.8 (RP11-1381A15 *NPIPA7*), H15.1 (RP11-344H15 *PKD1P6-NPIPP1*), and O14.1 (RP11-1236O14 *NPIPA1*) each show a ubiquitous pattern of tissue expression. The H15.1 line shows two distinct bands because this locus contains a tandem duplication of exon 2 resulting two sets of products by RT-PCR. **c** Organization and sequence composition of large-insert BAC clones used in mouse transgenic experiments. Annotations include SDs (colored arrows), gene models with the direction of transcription, and DupMasker annotation [20]. The baboon and three distinct human *NPIP* loci exhibit a diverse set of flanking duplicons and LCR16a copies

Because apes and monkeys show such dramatic differences in *NPIP* tissue expression, we performed mouse transgenesis experiments to determine if the differences in the two patterns of expression evolved in *cis* or *trans*. We constructed multiple independent mouse transgenic lines by pronuclear microinjection (see the “Methods” section and Additional file 1: Methods) and random integration of BACs carrying genomic copies of *NPIP.* We selected three human BACs (RP11-344H15, RP11-1381A15, RP11-1236O14) corresponding to different *NPIP* paralogs (*NPIPA1*, *PKD1P6-NPIPP1*, *NPIPA7*) and one baboon (RP41-285I13) corresponding to the single copy in that species that mapped to the ancestral locus common among all primates. We generated two founder mice for each line where mice carried full-length inserts and showed evidence of *NPIP* expression. The founder mice (A15.26, A15.3, O14.20, O14.23, H15.1, H15.2, I13.43, and I13.49) were crossbred with littermates to obtain homozygous stocks. We assessed expression of *NPIP* in both human and baboon by RT-PCR and then compared expression from seven tissues from six of the transgenic lines (A15.26, A15.3, O14.20, H15.1, I13.43, and I13.49). For all transgenic lines derived from human random integrations (A15.26, A15.3, O14.20, and H15.1), *NPIP* expression was detected in all seven tissues (Fig. 6) consistent with the broader expression pattern observed among apes (Fig. 5a). A15.26 and A15.3 represent independent integrations from the same BAC and the same human locus. For the mouse lines derived from the baboon BAC, we observed robust expression in the testis for both lines, which represented independent integrations. We did observe weaker expression patterns in the brain and kidney for line I13.49 (Fig. 6a) in contrast to line I13.43 (Fig. 6b), which showed marginal signals in the brain. This is likely due to position effects of the different integrations of the BACs into the mouse genome. The robust expression pattern in testis closely resembles baboon RT-PCR results (Additional file 1: Fig. S8). Sequencing of the baboon RT-PCR and the mouse transgenic found that only one of the three alternative splice isoforms maintains a predicted ORF and that this ORF carried the additional NHP exons (7.1, 7.2 and 7.3) shared among monkey species but absent in African apes. For both human and baboon, the overall differences in expression of the transgenic mice recapitulated that observed in primary tissues from the same species.

**Fig. 7:**
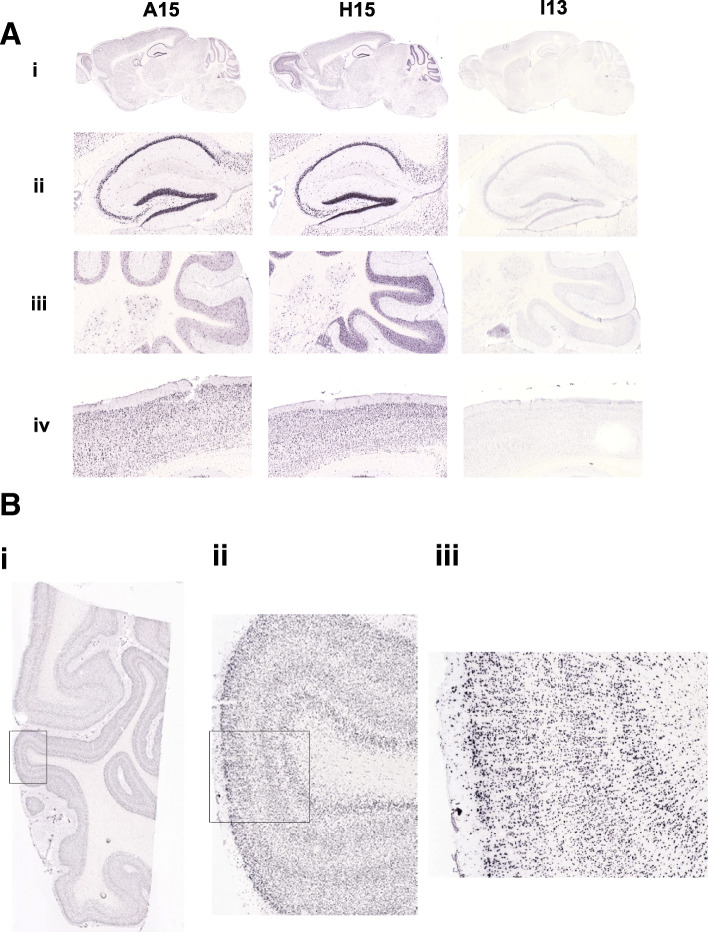
In situ hybridization of *NPIP* transgenic lines in brain tissue. **a** The transgenic line is indicated in each column (A15 (human), H15 (human), and I13 (baboon), respectively) and a representative sagittal section from each transgenic line is shown. (i) Expression of *NPIP* is undetectable in I13. (ii) Expression in hippocampal subregions. High expression is evident in the hippocampus pyramidal cells for both the A15 and H15 lines (derived from human BAC integrations). (iii) Cerebellar expression. High, widespread expression is apparent in the cerebellar granule cells and molecular layer in H15. In A15, large scattered cells in the granule layer are expressing *NPIP*. (iv) Examples of cortical expression patterns. High, widespread expression is evident in the cortex for both the A15 and H15 lines. **b** In situ hybridization of *NPIP* to human visual cortex. Human visual cortex, containing Brodmann’s areas 17 and 18, is shown hybridized to the riboprobe to the *NPIP* in the H15 transgenic line. Strong widespread expression is clearly visible throughout the visual cortex gray matter (ii and iii) as well as the white matter (i)

We also compared patterns of in situ hybridization (ISH) expression analysis for the BAC transgenic mice focusing specifically on a more detailed analysis of the brain. Because the *NPIP* gene family has been a target of positive selection in the human lineage compared to OWM, we designed ISH probes to the homologous region for humans and OWM cDNA separately (Additional file 1: Methods). Analysis of the ISH expression patterns in the H15 brains revealed that *NPIP* is expressed at high levels throughout the brain in easily definable cell types, specifically localizing to the nucleus of neurons (see Fig. 7a for representative images from one animal). In contrast, *NPIP* expression in the I13 transgenic line (baboon transgenic) was not detected by ISH in the brain (Fig. 7a) for all six I13 transgenic animals tested. The A15 (human) transgenic lines produced a more heterogeneous expression pattern in the brain that varied from strong widespread expression (similar to the expression pattern seen in H15) to less widespread expression, with various levels of sparseness in the expression pattern for all six A15 animals examined. Analysis of gene expression in the human visual cortex (containing Brodmann’s areas 17 and 18) (Fig. 7b) and human temporal cortex (containing Brodmann’s areas 21 and 22) was similar to the results obtained with the H15 transgenic line (Fig. 7b) confirming widespread expression among human cortical neurons. These observations, however, should be regarded with caution because of potential genetic background effects from the non-isogenic mice, variability in the number of copy number of BAC transgenes, and the fact that relatively few independent integrations have been studied for each construct.

## Discussion

The goal of this research was to provide insight into the formation and origin of interspersed duplications given their importance in both gene innovation and recurrent disease rearrangements. Human chromosome 16 stands out as being particularly enriched [12]. There are at least 15 blocks of interspersed duplications that now predispose to at least four recurrent rearrangements in humans [21], including the second most common cause of autism [22]. All interspersed duplication blocks on chromosome 16 are associated with a transcriptionally active, low-copy repeat sequence, LCR16a. In this 20-year study, we focused on understanding the evolution of LCR16a providing a framework for its origin, dispersal, and transcriptional potential among primates. Its association with large blocks of high-identity duplications makes this task particularly challenging because reference genomes have consistently failed to assemble these regions or discriminate among copies or transcripts. Thus, much of the work entailed the recovery, sequencing, and manipulation of large-insert BAC clones in order to understand its evolution and transcription. This large-scale comparative sequence study of 114 LCR16a loci from nine primate lineages provides some key insights into the mechanism of SD formation and how new gene families evolve.

First, we demonstrate that LCR16a exhibits an inherent property to recurrently duplicate in an interspersed fashion. Both large-scale comparative sequencing and phylogenetic analyses confirm LCR16a and its associated *NPIP* family have expanded independently in at least five different primate lineages. The process has seeded LCR16a to non-orthologous positions in different primate genomes, including different chromosomes. Although LCR16a duplication was originally thought to be specific to the apes [13, 14], our survey of NWM lineages provides evidence for an independent expansion of at least 11 copies in marmoset indicating that LCR16a’s propensity to duplicate has persisted for at least 35 million years. The presence of the *NPIP* intron-exon structure implies duplicative transposition as opposed to retrotransposition as the underlying mechanism for its dispersal.

Second, new insertions are nonrandomly distributed with a preference to the short arm of chromosome 16 (human phylogenetic group). In each primate lineage, many of the new insertions map within 5 Mbp (Fig. 1) of the ancestral location of LCR16a, which itself has been targeted by subsequent rounds of LCR16a duplication (Fig. 4). Nevertheless, LCR16a has also “colonized” other chromosomes where it has subsequently propagated, including chromosome 13 in orangutan [14], chromosome 11 in marmoset, and chromosome 17 in chimpanzee [14]. The only common feature of the acceptor regions is that that they tend to be GC-rich and enriched for SINE (in particular Alu) repeats (Additional file 1: Fig. S2 and S3). Unlike other African ape duplicated loci, which are largely intrachromosomal in their distribution, marmoset shows the greatest interchromosomal dispersal with LCR16a distributed to six different chromosomes in addition to chromosome 16p. LCR16a copies that have expanded to these new chromosomes are more closely related phylogenetically and map within a few megabase pairs of each other, consistent with a serial expansion of LCR16a in each lineage (Fig. 1). We propose that these unique patterns of interspersed duplications create lineage-specific hotspots of copy number variation predisposing these regions to non-allelic homologous recombination and large-scale variation associated with disease as has already been observed for human [21] and chimpanzee-specific SDs [23].

Third, sequencing of LCR16a genomic loci in different primate lineages has shown, with few exceptions, that the duplications do not occur in isolation but are accompanied by flanking SDs, ranging in size from ~ 15 to 180 kbp (Fig. 3). These flanking or “donor” segments are GC-rich and significantly enriched in SINE repeat content (Additional file 1: Fig. S2 and S3). They contain genes or parts of genes, are often lineage-specific, and concatenate to form large mosaic structures often hundreds of kilobase pairs in length. In general, duplication blocks located in close proximity are more similar with respect to their sequence composition and phylogenetically more closely related (Fig. 3). In the marmoset genome, for example, we characterize LCR16a-associated duplication blocks that span at least 150 kbp in length. The chromosome 16 and 11 copies are phylogenetically distinct with chromosome 11 copies sharing multiple flanking SDs when compared to marmoset chromosome 16. Of these marmoset duplicons, ~ 52% (12/23) are lineage-specific, with most sequences showing homology to human RefSeq gene annotations, such as a marmoset-specific duplication of *RIN2*, a gene encoding the RAB5 protein involved in both cellular signal transduction and the regulation of endocytoplasmic protein trafficking [24]. Studies in humans have shown that these gene-rich duplications flanking core duplicons such as LCR16a are associated with gene innovations implicated in the expanded prefrontal cortex, extended neural neoteny, increased synaptic connectivity, and other potentially unique human adaptations [4–7]. As such, the discovery of these lineage-specific duplicates flanking LCR16a represents candidates for species-specific adaptations [25] in other species such as marmoset.

Fourth, LCR16a duplication integrations are consistently associated with the loss of corresponding sequence at the site of integration. Sequence analysis of 13 integrations in NHP genomes when compared to human finds that 12 of the sites (92%) show a loss of repeat-rich sequence ranging in length from 3.4 to 80.1 kbp (median 5.8-kbp deletion). Only one locus in marmoset showed a precise integration with no associated loss of intervening sequence at the pre-integration site. In addition to deletions, comparative breakpoint analyses of great ape genomes have shown that LCR16a insertions often delineate the boundaries of large-scale inversions [7]. Our analysis of marmoset extends the association of LCR16a to the breakpoints of large-scale evolutionary chromosome rearrangements such as those leading to formation of marmoset chromosomes CJA5 and CJA11 [15, 16, 26]. We recently reported a similar association of chromosomal evolutionary rearrangements with another interchromosomal core duplicon, *OR7E* [27]. Although the cause-and-effect relationship cannot be determined, this association with deletions and larger genome instability events suggests double-strand breakage of DNA and is reminiscent of replication-based pathways proposed to explain the origin of SDs in yeast [28].

It is interesting in this context that the only common feature of the LCR16a acceptor and donor regions are that that they tend to be GC-rich and significantly enriched for SINE (in particular Alu) repeats (Additional file 1: Fig. S2 and S3). This independent association with Alu repeats in multiple primate lineages may provide some insight into mechanism of origin and propagation. Among common repeat elements, Alu repeats are known to be enriched and possibly selected for in early replicating GC-rich regions of the genome [29], they are preferential sites for structural variation and segmental duplication possibly due to homology directed repair/recombination [30, 31], and Alu-rich DNA appears to be organized in the interphase nucleus along the surface of chromatin facing the nuclear envelope [32]. These apparently unique properties of this repeat may facilitate repair and replication of non-allelic homologous segments of DNA through homology, proximity, and accessibility leading to the enrichment of Alu repeats at donor, acceptor, and breakpoint regions. Moreover, the fact that the primate-specific Alu repeat subfamily experienced a burst of retrotransposition 40 million years ago [33] may also explain why these complex LCR16a-associated duplications have been largely restricted to NWM and OWM species such as the apes. These observations are consistent with the broader hypothesis that the expansion of the Alu repeat mobile element sensitized primate genome to segmental duplications [30, 34].

Transcript analysis of LCR16a shows that these independent primate expansions of LCR16a have occurred against a backdrop of remarkable restructuring of the encoded *NPIP* family gene model in different primates. This has led to the wholesale gain and loss of exons creating new isoforms that bear little resemblance to each other or ancestral reconstructions despite the maintenance of an ORF. In this regard, it is interesting that the proportion of Alu repeat sequences has continued to increase at the *NPIP* locus over the course of primate evolution with the number of intronic Alu repeats more than doubling since divergence of the OWM and NWM lineages (Fig. 5). Sequence analysis indicates that this repeat enrichment has contributed in some cases to Alu-mediated rearrangements as well as potentially altered splicing patterns [35]. In addition, Alu insertions have even altered the predicted ORF. For example, we observed a partial AluY insertion in the carboxy terminal repeat unit of the African ape *NPIPB* isoform (Fig. 5c). This insertion creates a frameshift leading to a highly divergent amino acid repeat array, an event that preceded the evolution of *NPIPB* isoform, which is specific to the African ape lineage. Such rapid evolutionary turnover in gene structure has been described for other loci where Alu repeats are abundant, such as in *BRCA1* [36, 37].

It may be noteworthy that copies of LCR16a that have become isolated onto new chromosomes appear to be more likely to become pseudogenized and transcriptionally inactive. Humans, chimpanzees, and gorillas, for example, share one solo LCR16a that duplicatively transposed to the African ape ancestral chromosome 18 [13] based on current reference genomes. This locus neither is transcribed nor maintains an ORF and has been described as a pseudogene (*NPIPBP1*). Comparative and phylogenetic analyses of the chromosome 18 ape locus indicate that it has not subsequently propagated (Additional file 1: Fig. S6). Similarly, we find no evidence of transcription for the four marmoset copies that map as single copies to chromosomes 4, 20, chrUkn, and 17/13. Although our evidence is limited to these five loci of LCR16a, phylogenetic analysis shows that these solo copies fail to produce progeny. This is in stark contrast to chromosomes that carry multiple copies where most of the transcripts map (e.g., chromosomes 11 and 16 in marmoset) and where the phylogeny (Fig. 3) indicate successive rounds of intrachromosomal duplication. These observations suggest that that transcription of *NPIP* and duplication may be fundamentally linked.

**Fig. 8:**
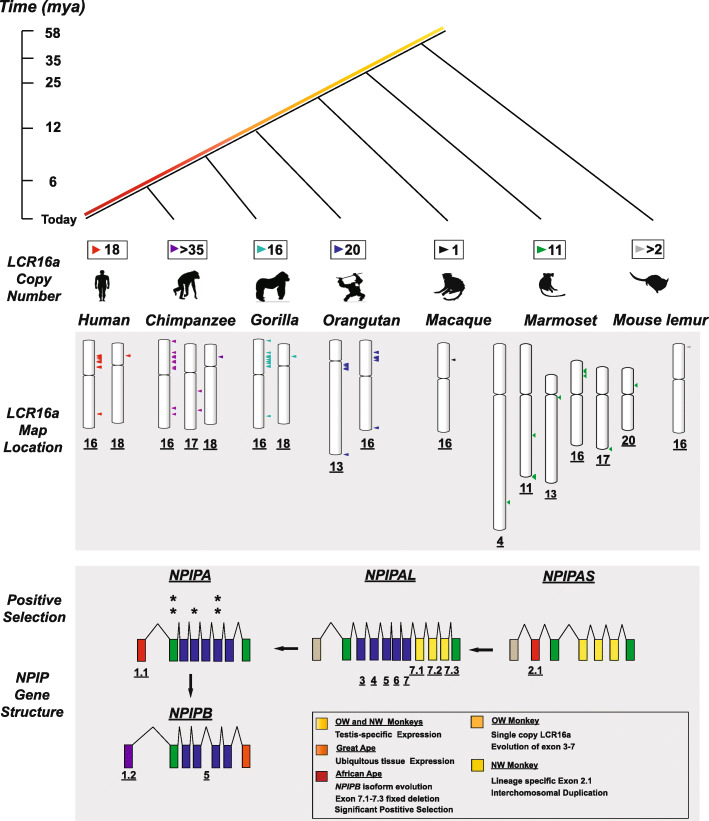
Model of NPIP/LCR16a evolution. Comparative analysis in primates reveals changes associated with both LCR16a duplication and the *NPIP* model. Diversity in map location, structural variation, expression, and selection of the *NPIP* family has occurred over ~ 58 mya of primate evolution. Note the disproportionate amount of change in the great ape lineage

The *NPIP* family was originally identified as one of most dramatic examples of positive selection in the African ape lineage due to an excess of amino acid replacements occurring over exons 2 and 4 of the canonical human gene model [13]. Our broader survey of primate loci and *NPIP* gene models extends these observations to also include exon 6 (Additional file 2: Table S5 and Additional file 1: Fig. S10). Notably, both marmoset and macaque gene models show a pattern of amino acid replacements indistinguishable from neutral evolution. The identification of an ortholog in dog generally confirms the overall ancestral gene structure and suggests the gene has been evolving neutrally for at least 70 million years (Additional file 1: Fig. S14 and S15). The evolutionary transition from neutral to positive selection occurs after a dramatic change in *NPIP* expression (Fig. 8). Our analysis of OWM and NWM species suggests that the ancestral *NPIP* expression was largely restricted to the testis, in contrast to both African and Asian apes where *NPIP* is expressed in all tissues (Fig. 5a).

BAC transgenic experiments in mouse with three different human *NPIP* paralogs confirm a broad expression profile for each human copy in contrast to the baboon, which shows signal predominantly in the testis (Fig. 6). The fact that three distinct human *NPIP* loci with diverse flanking duplicons (Fig. 6c) and integration sites show a similar broad pattern of expression in all tissues argues that this shift to a more ubiquitous pattern of expression evolved in *cis* in the ancestral ape paralog prior to the gene family’s expansion among the African apes. A more detailed in situ analysis of the brain (with both humanized mouse transgenic as well as human tissue) shows strong signal to the nuclei of neurons (Fig. 7). These findings are consistent with immunostaining and GFP-fusion transfection experiments, which localized *NPIP* to the outer photoreceptor cone segments of the retina and the nucleus of neuroblastoma cell lines [38].

Our comparative analyses suggest that the widespread neuronal expression of this gene family occurred early in great ape evolution prior to the major bouts of exonic positive selection in the African ape lineage, but that the propensity of LCR16a to create interspersed duplications is a far more ancient property. A critical next step in this research is a determination of the molecular function and phenotypic consequence of the gain and loss of individual *NPIP* genes. At present, there are only a few clues. Consistent with the BAC transgenic in situ experiments, *NPIP* has been reported to be highly expressed (eightfold) in the foveomacula when compared to the retina [38]—second in transcript abundance only after the cone opsin genes. In a separate study, analysis of the single-cell gene expression data from primate cerebral organoid tissue reveals that one member, *NPIPB5*, shows distinguishable levels of human-specific gene expression in excitatory neurons and radial glia cells [39]. This is particularly noteworthy because the *NPIPB* subfamily has expanded almost exclusively in chimpanzee, human, and gorilla and is underlying much of the African ape positive selection. There is also evidence that the gene family continues to experience rapid positive selection specifically in the human lineage. For example, we recently identified a previously unknown member of the *NPIPB* family, *NPIPB16*, mapping to a 383-kbp Melanesian-specific duplication that originated ~ 400 kya in a Denisova hominin [40]. Despite its recent origin, *NPIPB16* is expressed, maintains an ORF, and shows strong signals of positive selection (~ 3% amino acid divergence) consistent with other gene paralogs we report here. Remarkably, the 383-kbp segment of DNA that introgressed into the Melanesian ancestral populations ~ 40,000 kya has now risen to high frequency (> 79%) among modern-day Papuan populations likely as a result of partial selective sweep. These findings suggest that *NPIP* continues to not only restructure hominin genomes but also rapidly adapt and evolve possibly in response to a pressure or agent still challenging the human and African great ape species more broadly.

## Conclusion

In this study, we characterize the evolution and transcript diversity for a 20-kbp core duplicon, LCR16a, and its encoded gene family nuclear pore interacting protein. Using large-insert clone resources and long-read genomic/transcriptomic sequencing, we investigate high-identity duplicated sequence largely intractable to standard genome assembly approaches. Our results strongly support a model where LCR16a has independently driven the accumulation of interspersed primate SDs in conjunction with the evolution of a transcribed gene family undergoing signals of strong adaptive evolution.

## Methods

### Library hybridization and BAC end sequencing

LCR16a hybridizations on four large-insert clone genomic BAC libraries (average sixfold coverage) representing marmoset (CHORI-259), squirrel monkey (CHORI-254), owl monkey (CHORI-258), and gray mouse lemur (CHORI-257) were performed as previously described [14, 41]. PCR-amplified products (Additional file 2: Table S6) derived from LCR16a sequences were used as radioactive probes, and 169 total LCR16a-positive BACs were recovered. LCR16a copy number was estimated in each lineage by taking the ratio of recovered clones by the estimated clone coverage per library (Table 1). A subset of LCR16a-positive BAC clones from the CH259 library were also validated by PCR-based screening (Additional file 1: Fig. S16). In order to prioritize clones for high-quality sequence and assembly, we used repeat-masked BAC end sequence pairs that were rescored for quality and mapped against the human reference genome (GRCh38). In the absence of BAC-end sequence placements, we also sequenced a subset of BAC clones using short-read Illumina sequencing as outlined below.

### Sequencing and assembly of BAC inserts

High-quality sequence and assembly of large-insert clones was performed as previously described [42, 43]. In brief, DNA from human (CH17) and NHP (CH251, CH276, CH277, CH250, CH259) BAC clone libraries were isolated, prepped into bar-coded genomic libraries, and sequenced (PE101) on a Illumina MiSeq or HiSeq 2500 using a Nextera protocol described previously [44]. Sequence data were mapped with mrsFAST [45] to the GRCh38 reference genome, and singly unique nucleotide (SUN) identifiers were used to discriminate between highly identical SDs [46]. We pooled nonoverlapping BACs at equal molar amounts before library preparation, and SMRTbell libraries were prepared and sequenced using RS II C2P6 chemistry on the PacBio SMRT sequencer (Pacific Biosciences, Inc., Menlo Park, CA). Inserts were assembled using the Canu assembler [47] followed by consensus sequence calling using Quiver [48]. PacBio clone inserts were reviewed for misassembly by mapping clone end sequences back to the insert and visualizing read depth of PacBio reads in Parasight (http://eichlerlab.gs.washington.edu/jeff/parasight/index.html) using coverage summaries generated during the resequencing protocol. Contig assembly was performed using Sequencher (Gene Codes Corporation, Ann Arbor, MI) and compared to the human reference genome (GRCh38) using Miropeats [49] and BLAST [50].

### Sequence, selection, and duplication analysis

SDs were annotated within individual contigs using a combination of WSSD [1], DupMasker [20], and a modified version of whole-genome assembly comparison (WGAC) [2]. Comparative sequence analysis between reference and large-insert clone-based assemblies was performed using aligners: BLASR [51] with parameters fine-tuned for contig alignments (-bestn 1 -minAlignLength 1000 -m 1 -alignContigs –piecewise), BLAT [52], and BLAST [50]. Breakpoints were refined using local sequence alignments performed using MAFFT [53]. For all coding exons (exon 8 was omitted due to complications optimizing global alignments of the repeat array), the average number of synonymous (dS) and non-synonymous (dN) substitutions per site were estimated using the modified Nei-Gojobori method [54]. We extracted coding sequences from high-quality contiguous BAC sequence (~ 79) and from working draft assemblies primarily ordered and orientated into multiple contigs using Sanger sequencing (~ 72). In order to create optimal global alignments for downstream analysis, groups of finished and unfinished clones were analyzed separately. To test for positive Darwinian selection at the level of single exons, we calculated the difference between dN and dS (*D* = dN − dS) within primate groups (defined as species) for all pairwise comparisons of paralogues [13] and implemented a one-tailed *Z*-test (*Z* = *D*/*ơ*) to determine the level of significance (HSA = human, GGO = gorilla, PTR = chimpanzee, PPY = orangutan, OWM = baboon and macaque, CJA = marmoset). dN/dS quotients were also compared between primate groups with LCR16a duplication and the OWM lineage, which represents only a single copy of LCR16a. A maximum likelihood analysis using the entire gene model was also performed (with the exclusion of exon 1 and exon 8, which are highly variable among loci) using PAML [19] with phylogenies reconstructed using the maximum likelihood based method in IQ-TREE [55] (Additional file 1: Methods). The phylogeny included sequences representing African apes (7), great apes (7), OWM (2), and a wider mammalian outgroup that includes two LCR16a copies identified in dog. Note that we excluded the marmoset paralogs in this analysis due to the dramatic restructuring of the NWM gene model.

### Phylogenetic analyses

We generated multiple sequence alignments using MAFFT [53] from (human, chimpanzee, gorilla, orangutan, macaque, gibbon, baboon, marmoset, squirrel monkey, and gray mouse lemur) orthologous and paralogous sequences. We constructed unrooted phylogenetic trees using the neighbor-joining method (MEGA5) [56]. Genetic distances were computed using the Kimura two-parameter method with standard error estimates and interior branch test of phylogeny (*n* = 500 bootstrap replicates). Tajima’s relative rate test (MEGA5) was used to assess branch length neutrality. We estimated the coalescence of time using the equation *R* = *K*/2*T*, assuming a chimpanzee–human divergence time (T) of 6–7 mya for chimpanzee, 15 mya for orangutan, and 25 mya for macaque. Phylogenetic group designations based on synteny to human chromosomes [57] were used when referring to chromosomal band positions in NHPs unless otherwise indicated. When species-specific chromosomal nomenclature was used, we applied the shorthand convention of the species name followed by the chromosome number (e.g., CJA5 = *Callithrix jacchus* (marmoset) chromosome 5).

### FISH analysis

Single-color metaphase FISH was performed using lymphoblast cell lines obtained from marmoset (CJA) from The Biomedical Primate Research Centre (Netherlands). FISH experiments were performed using the following human clones derived from the RPCI-11 BAC library—chr11: RP11-265F9, chr17: RP11-481P7 and chr13: RP11-110 K18—directly labeled by nick-translation with Cy3-dUTP (PerkinElmer) as described previously [58] with minor modifications [27].

### Tissue samples

Chimpanzee (*Pan troglodytes*) tissue material (testis, pancreas, brain stem, cerebellum, medulla oblongata, thalamus, spleen, heart, small and large intestine) was obtained < 8 h post-mortem from a male specimen from the Southwest Foundation for Biomedical Research (courtesy of Jerilyn Pecotte) San Antonio, TX 78227, USA. Orangutan (*Pongo pygmaeus*) tissue samples were obtained post-mortem (Dan Anderson, Yerkes Primate Center Atlanta, GA 30329, USA) from two different male orangutan specimens (YN98-329/Gelar for spleen, liver, brain; and YN98-389/Ayer for liver and heart). Both macaque (*Macaca mulatta*) and baboon (*Papio anubis*) tissues were obtained from euthanized specimens at Southwest Foundation for Biomedical Research.

### RT-PCR analysis

Total RNA was extracted from tissue panels of the primates or mice transgenic animals using the RNeasy® Mini or Midi Kit from Qiagen. Tissues were homogenized using an OMNI rotor in a mixture of 300 μL buffer RLT plus 590 μL of RNase free water. The on-column DNase treatment was skipped, and total RNA was DNase treated with the Amnbion Turbo DNase kit. RNA quality and quantity were assessed by agarose gel electrophoresis. c-DNA were prepared using 1 μg of total RNA (Powersript™ Reverse Transcriptase from Clontech/Takara Bio kit and protocol). PCR reactions were performed using 1 μL of cDNA, Qiagen master mix, and primers specific for the ubiquitin-activating enzyme 1 (UBE1) and *NPIP* family (Additional file 2: Table S7). Cycling conditions consisted of 35 cycles with an annealing temperature of 55°. PCR products were run on a 1% agarose gel with 0.5 μg of 100 bp ladder.

### Full-length cDNA sequencing

We performed full-length cDNA capture and isoform sequencing as previously described [3]. In brief, we designed a set of complementary oligonucleotide capture probes (Additional file 2: Table S8) to enrich for cDNA originating from *NPIP* paralogous copies and coupled this with a method to enrich for full-length cDNA molecules based on reverse transcriptase (RT) template switching [59]. Next, we generated PacBio Iso-Seq libraries and performed post-capture size selection to enrich for larger cDNA molecules according to the manufacturer’s guidelines (SMRTbell template prep kit 1.0, PacBio). SMRT sequencing was performed using the P6-C4 chemistry on the PacBio RS II instrument with 6-h movies [3]. A modified version of the Iso-Seq bioinformatics incorporating ToFU (Transcript isOforms: Full-length and Unassembled) was used for processing the long-read RNA-seq data (available at https://github.com/EichlerLab/isoseq_pipeline). Circular consensus sequence reads designated as putatively full length (if the expected terminal sequences and a poly(A) tract were observed) were then mapped to large-insert clone-assembled custom contigs using GMAP (v 2015-07-23) [60]. ORFs were identified using ANGEL (https://github.com/PacificBiosciences/ANGEL) and TRANSLATE as part of the ExPASy: SIB bioinformatics resource portal [61].

### BAC transgenic

We generated random integration mouse transgenic lines from three human *NPIP*-containing BACs [RP11-1381A15 (AC141267), RP11-1236O14 (AC142080), and RP11-344H15 (AC092137)] obtained from the human RPCI-11 library and one OWM BAC, RP41-285I13 (AC092562) from the baboon RPCI-41 library. BAC DNA was purified using the Clontech Nucleobond column (Palo Alto, CA) followed by passage through a CL4B Sepharose column (Amersham Biosciences, Buckinghamshire, England) to obtain higher grade DNA. The column was equilibrated with injection buffer (10 mM Tris-HCl, pH 7.5, 0.1 mM EDTA, and 100 mM NaCl) and the DNA collected in 12 elution fractions. The appropriate fraction containing the BAC was diluted to a concentration ranging between 0.6 and 1.0 ng/μl. Transgenic mice were generated by direct microinjection of BAC DNA into the pronuclei of fertilized mouse eggs. This method was performed as previously described [62]. The embryos injected were F2 progeny of a C57BL6/SJL F1 cross and were surgically transferred into the oviducts of a pseudopregnant CD-1 female. Founder mice transgenic for BAC clones corresponding to the LCR16a locus were identified by PCR from mouse tail DNA followed by Southern Blot hybridization. Hybridization was done at 65 °C with Church and Gilbert Solution (500 mM NaPO4, 1 mM EDTA, 1% bovine serum albumin, 7% SDS). Blots were washed with 0.2x SSC and 0.5% SDS at 65 °C.

### In situ hybridization (ISH)

Serial 25-μm fresh-frozen cryostat sections were systematically collected from male mice starting at a standardized sagittal plane of section in the brain to ensure reproducible anatomical coverage. High-throughput data generation was performed using a nonradioactive colorimetric ISH protocol as described previously [63]. Briefly, riboprobes labeled with digoxigenin were hybridized to post-fixed sections on an automated Tecan platform, using tyramide signal amplification (tyramide biotin) to amplify the signal and alkaline-phosphatase to catalyze the colorimetric reaction. Following the completion of the ISH protocol, an acid alcohol step was performed to reduce background signal. An automated image capture platform digitized the ISH data. In brief, a Leica DM6000B microscope, with a Leica DC500 camera, is mounted on an air table to isolate the microscope from external sources of vibration that would affect image quality. The image capture procedure is essentially fully automated and collects each image section at × 10 magnification at a resolution of approximately 1.0 μm/pixel and stores the image directly into the JPEG2000 format as previously described [63]. For ISH on human tissue, 20-μm thick tissue samples were sectioned in the coronal plane and slides were organized in groups of four, representing four tissue sections spaced 1 mm apart across the sample. After sectioning, the tissue is fixed, acetylated, and dehydrated. A nonradioactive colorimetric ISH protocol was used. Following the completion of the ISH protocol, an acid alcohol step was performed to reduce background signal. Image acquisition was completed using ScanScope® scanners (Aperio Technologies, Inc., Vista, CA). The ScanScope scanner uses a × 20 objective that is downsampled in software. The downsampling provides image resolution at approximately 1 μm/pixel. The human tissue ISH protocol has been previously described [63].

### Permutation testing

We tested for enrichment of three genomic features (i.e., GC, SINE and LINE content) for SDs found in association with LCR16a (i.e., donor segments, *n* = 95), for unique sequences flanking where LCR16a duplication blocks integrated (i.e., acceptor sites, *n* = 37) or sequence that existed at the integration site prior to duplication (i.e., pre-integration loci, *n* = 13). All coordinates were based on the human genome reference, GRCh38, and redundant donor, and acceptors sites were merged to generate 63 and 27 nonredundant regions, respectively. We identified acceptor sites as the nearest contiguous 10 kbp of unique sequence flanking the duplication block containing LCR16a. SINE and LINE element annotations were extracted from the RepeatMasker tracks of GRCh38. The null distribution for each of the three features was generated using 10,000 permutations excluding SDs, centromeres, telomeres, and gaps in the GRCh38 assembly. BEDTools version 2.28.0 and R version 3.6.0 were used for the generation of the null and computing statistical significance, respectively. The Kolmogorov-Smirnov test was used to establish the statistical significance of the difference between the observed GC content and the null, while an empirical *p* value was calculated for the SINE and LINE enrichment tests via the *Z*-score transformation. Multiple testing correction (i.e., Bonferroni correction) was applied to *p* values assuming a total of nine tests. The raw *p* values were assumed to follow a Binomial distribution, allowing us to estimate the Standard Error (SE) using the following formula:

$$ \mathrm{SE}=\sqrt{\frac{p\times \left(1-p\right)}{n.}} $$

where *p* is the *p* value and *n* is the number of regions tested.

## Supplementary information

**Additional file 1: **Methods. **Figure S1.** 11 LCR16a marmoset insertions anchored to the GRCh38 reference genome. **Figure S2.** Sequence properties and enrichment analysis of donor and acceptor regions in association with LCR16a. **Figure S3.** Breakpoint resolution from eight LCR16a insertions. **Figure S4.** Evidence of recurrent LCR16a duplication during primate evolution. **Figure S5.** Evolutionary analysis and timing estimates of LCR16a copies in primates. **Figure S6.** Evolutionary analysis of LCR16a copies in great apes. **Figure S7.** Lineage-specific duplicate genes. **Figure S8.** *NPIP* expression in a diversity panel of tissues/subtissues originating from human and NHP primary source material. **Figure S9.** Gene amino acid structure of *NPIP* isoforms throughout primate evolution. **Figure S10.** Selection analysis across four *NPIP*-coding exons based on an excess of nonsynonymous amino acid replacements. **Figure S11.** Evidence for positive selection in the African ape lineages using branch model analysis (PAML). **Figure S12.** Evidence for positively selected amino acid sites within the African ape lineage. **Figure S13.** *NPIP* gene fusion transcripts detected using PacBio Iso-Seq and RT-PCR. **Figure S14.** NPIP protein alignment and exon comparison between dog, macaque and human. **Figure S15.** Cross-species comparison of the predicted ancestral *NPIP* organization between macaque, mouse lemur and dog. **Figure S16.** PCR-based testing of LCR16a-positive BAC clones derived from the CH259 large-insert clone library. **Figure S17.** ISH expression analysis for BAC transgenic mice. **Figure S18.** Evidence for positive selection in the *NPIPB* subtype using branch model analysis (PAML). **Figure S19.** Evidence for positive selection relating to the NPIPB subtype within the African ape lineage.

**Additional file 2: Table S1.** List of large-insert clones from OWM, NWM and prosimian lineages. **Table S2.** List of LCR16a positive clones from apes. **Table S3.** Sequence contigs generated using large-insert BAC clones. **Table S4.** Characteristics of LCR16a-associated duplications. **Table S5.** dN/dS ratio analysis (synonymous/nonsynonymous) for individual NPIP exons. **Table S6.** Primer sequences for BAC hybridization probes. **Table S7.** Primer sequences for RT-PCR expression experiments. **Table S8.** Oligonucleotide capture probes used for Iso-Seq analysis.

**Additional file 3.** Review history.

## Data Availability

All data have been deposited in NCBI GenBank and can be accessed under the following BioProject ID numbers PRJNA593285 [64] and PRJNA369439 [65] or from individual NCBI accession numbers listed in Additional file 2: Tables S1 and S2. Mouse transgenic strains can be accessed as part of the Jackson laboratory Cryo recovery service (https://www.jax.org/mouse-search?searchTerm=NPIP).
