# Supplementary material for: An evolutionary driver of interspersed segmental duplications in primates
Source: Genome Biol. 2020 Aug 10;21:202. doi: 10.1186/s13059-020-02074-4 (PMC7419210; doi:10.1186/s13059-020-02074-4)
Supplement: Supplementary file 1 — Additional file 1: Methods. Figure S1. 11 LCR16a marmoset insertions anchored to the GRCh38 reference genome. Figure S2. Sequence properties and enrichment analysis of donor and acceptor regions in association with LCR16a. Figure S3. Breakpoint resolution from eight LCR16a insertions. Figure S4. Evidence of recurrent LCR16a duplication during primate evolution. Figure S5. Evolutionary analysis and timing estimates of LCR16a copies in primates. Figure S6. Evolutionary analysis of LCR16a copies in great apes. Figure S7. Lineage-specific duplicate genes. Figure S8. NPIP expression in a diversity panel of tissues/subtissues originating from human and NHP primary source material. Figure S9. Gene amino acid structure of NPIP isoforms throughout primate evolution. Figure S10. Selection analysis across four NPIP-coding exons based on an excess of nonsynonymous amino acid replacements. Figure S11. Evidence for positive selection in the African ape lineages using branch model analysis (PAML). Figure S12. Evidence for positively selected amino acid sites within the African ape lineage. Figure S13. NPIP gene fusion transcripts detected using PacBio Iso-Seq and RT-PCR. Figure S14. NPIP protein alignment and exon comparison between dog, macaque and human. Figure S15. Cross-species comparison of the predicted ancestral NPIP organization between macaque, mouse lemur and dog. Figure S16. PCR-based testing of LCR16a-positive BAC clones derived from the CH259 large-insert clone library. Figure S17. ISH expression analysis for BAC transgenic mice. Figure S18. Evidence for positive selection in the NPIPB subtype using branch model analysis (PAML). Figure S19. Evidence for positive selection relating to the NPIPB subtype within the African ape lineage. [file 13059_2020_2074_MOESM1_ESM.pdf]

An Evolutionary Driver of Interspersed Segmental Duplications in Primates

Stuart Cantsilieris[1,**2**], Susan M. Sunkin[3], Matthew E. Johnson[4], Fabio Anaclerio[5], John Huddleston[6,7], Carl Baker[1], Max L. Dougherty[1], Jason G. Underwood[8], Arvis Sulovari[1], PingHsun Hsieh[1], Yafei Mao[1], Claudia Rita Catacchio[5], Maika Malig[1,**9,10**], AnneMarie E. Welch[1,**11**], Melanie Sorensen[1], Katherine M. Munson[1], Weihong Jiang[12], Santhosh Girirajan[13], Mario Ventura[5], Bruce T. Lamb[14], Ronald A. Conlon[12], and Evan E. Eichler[1,15]*

[1]Department of Genome Sciences, University of Washington School of Medicine, Seattle, WA 98195, USA.
[2]Centre for Eye Research Australia, Department of Surgery (Ophthalmology), University of Melbourne, Royal Victorian Eye and Ear Hospital, East Melbourne, VIC 3002, Australia.
[3]Allen Institute for Brain Science, Seattle, WA, USA.
[4]Center for Spatial and Functional Genomics, Children's Hospital of Philadelphia, Philadelphia, PA 19104, USA.
[5]Department of Biology-Genetics, University of Bari, Bari, Italy.
[6]Vaccine and Infectious Disease Division, Fred Hutchinson Cancer Research Center, Seattle, WA 98109, USA.
[7]Molecular and Cellular Biology Program, University of Washington, Seattle, WA 98195, USA.
[8]Pacific Biosciences (PacBio) of California, Incorporated, Menlo Park, CA 94025, USA.
[9]Department of Molecular and Cellular Biology, University of California, Davis, CA 95616, USA.
[10]Integrative Genetics and Genomics Graduate Group, University of California, Davis, CA 95616, USA.
[11]Brain and Mitochondrial Research, Murdoch Children's Research Institute, Royal Children's Hospital, Melbourne, VIC, Australia.
[12]Case Transgenic and Targeting Facility, Department of Genetics and Genome Sciences, School of Medicine, Case Western Reserve University, Cleveland, OH 44106, USA.
[13]Department of Biochemistry and Molecular Biology, Department of Anthropology, Pennsylvania State University, University Park, PA 16802, USA.
[14]Stark Neurosciences Research Institute, Indiana University School of Medicine, Indianapolis, IN 46202, USA.
[15]Howard Hughes Medical Institute, University of Washington, Seattle, WA 98195, USA.

[#]Bold represent current affiliations.

*Corresponding author: Evan E. Eichler, Ph.D.
University of Washington School of Medicine
Howard Hughes Medical Institute
3720 15th Ave NE, S413C
Box 355065
Seattle, WA 98195-5065
Phone: (206) 543-9526
E-mail: eee@gs.washington.edu

Methods

Transgenic Mouse Lines

We established two independent lines for each transgenic. A15.26, A15.3, O14.20, O14.23, H15.1, and H15.2 corresponded to three human BACs (RP11-344H15, RP11-1381A15, and RP11-1236O14) and lines I13.43 and I13.49 corresponded to a single BAC from the baboon (RP41-285I13). RP11-1381A15 (205 kbp), which was used to generate transgenic mouse line A15, represented the transcriptional splice form of *NPIP* (*NPIPA7*), which contained all eight exons and produces a 1,070 bp transcript in humans. The BAC also contains the NODAL modulator 3 gene (*NOMO3*) downstream of *NPIP*. RP11-1236O14 (218 kbp), which was used to generate transgenic mouse line O14, represents the transcriptional splice form *NPIPA1* and contains all eight exons but had a 54 bp larger spliceform of exon 4. RP11-1236O14 also contains a full-length gene transcript of NODAL modulator 1 (*NOMO1*) located upstream of *NPIPA1*. RP11-344H15 (185 kbp), which was used to generate transgenic mouse line H15, expresses a variation of *NPIP* containing two copies of exon 2 in the full-length transcript. We identified three other full-length genes; the first two, RNA polymerase I transcription factor (*RRN3*) and N-terminal Asnamidase (*NTAN1*), reside downstream of the *NPIP* transcript. The third gene, polycystic kidney disease 1 (*PKD1*), is upstream of the *NPIP* transcript. Finally, transgenic mice were created by using the ancestral single-copy *NPIP* gene from baboon RP41-285I13 (184 kbp) and this line was referred to as I13. The BAC also contained two homologous genes (*NTAN1* and *RRN3*) downstream of the single-copy *NPIP* gene. We investigated expression patterns across two independent lines and in all transgenics (3 x Human: A15, O14, H15; 1 x Baboon: I13). RT-PCR experiments were performed from cDNA sourced from a panel of seven tissues that included brain, heart, kidney, liver, lung, muscle, and testis (data not shown). In each independent line the expression results were near identical. For all human BAC integrants we observed a pattern of ubiquitous expression, while both independent lines generated from the baboon showed expression specific to the testis.

In situ Hybridization (ISH)

For the ISH experiments, we proceeded with four human BAC transgenic lines representing two independent integrations (A15.26 and A15.3) of the same BAC, RP11-344H15, and two independent integrations (I13.43 and I13.49) of the baboon BAC, RP41-285I13. A control probe from the mouse locus, Drd1a, was used as a positive control for the ISH experiments and was hybridized to tissue sections from each of mouse transgenic lines. *NPIP* probes specific to the corresponding sequence in both baboon and human were designed based on cDNA sequence available for representative loci. In the case of baboon, we specifically designed a 659 bp probe derived from macaque cDNA (XM_001109190), which is 99% identical. In the case of human, we selected two probes based on available human cDNA (Additional file 2: Table S9). A homologous *NPIP* gene and/or the gene family does not appear to exist in the mouse. Sequence analysis of the corresponding homologous locus shows that it is highly divergent and lacks any significant sequence homology with the transcripts identified amongst primates. The three independent paralogs of the human *NPIP* versions gave near identical ISH results consistent with the observations from the primary tissue (Additional file 1: Fig. S17). We do note, however, there was some evidence of differences in levels of expression although no difference in the overall pattern with respect to the primary tissue was observed.

Maximum likelihood analysis using PAML

We performed a maximum likelihood analysis using the branch models of PAML [19] and the entire gene model with the exclusion of exons 1 and 8, which are highly variable among the *NPIP* copies from dog (n=2), macaque (n=1), baboon (n=1), orangutan (n=5), gorilla (n=5), chimpanzee (n=5), and human (n=7) (Additional file 1: Fig. S11-S12 and S18-S19). Note that we excluded the marmoset paralogs in this analysis due to the dramatic restructuring of the New World gene model. The input phylogeny is inferred using these 26 *NPIP* paralogs and the maximum likelihood-based method in IQ-TREE [48]. We applied the codon substitution models (codeml) implemented in PAML to test positive selection and constructed a null model under neutrality (w=1 on all branches; H0) for the entire inferred phylogeny (Additional file 1: Fig. S11). The significance of each test was evaluated using the likelihood ratio chi square test.

Selection analysis in canine lineages: To search for evidence for selection in the canine lineage, we set w=1 for all branches except the canine lineages (CAN1 and CAN2; Additional file 1: Fig. S11) and estimate w0 (H1).

Selection analysis in primate lineages: We first built a model (H2) to infer selection on all ape lineages (a single free parameter w:=w1=w2, and set w0=1; Additional file 1: Fig. S11), then considered two additional models: selection on the African ape sequences (H3: a free parameter w2, and set w0=w1=1) or only within the Old World monkey (OWM) and orangutan (H4: a free parameter w1, and set w0=w2=1). We also tested whether there was evidence for selection on either subfamily A (H5_A; Additional file 1: Fig. S18) or the subfamily B (H5_B; Additional file 1: Fig. S18) in the African ape lineages.

Positive selection sites model: To identify sites that are likely the targets of positive selection, we applied the branch-site test of positive selection implemented in PAML (model=2, NSsites=2, fix_omega=0) to the same 26 sequences from exons 2 to 7 with the same configuration in the models H3 (Additional file 1: Fig S12) and H5_B (Additional file 1: Fig. S19).

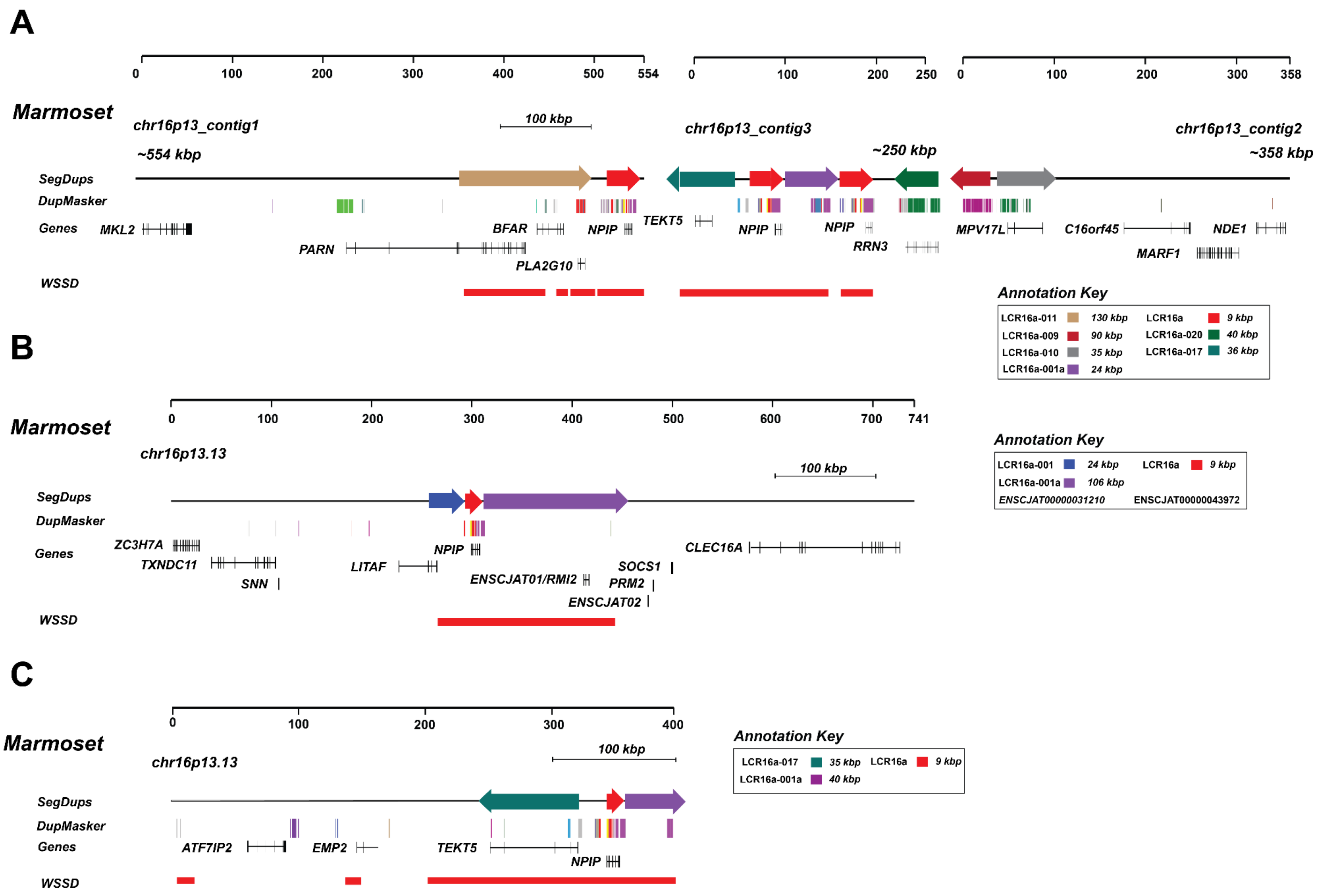

A
Marmoset
chr16p13_contig1
~554 kbp
chr16p13_contig3
~250 kbp
chr16p13_contig2
~358 kbp
100 kbp
SegDups
DupMasker
Genes
WSSD
MKL2
PARN
BFAR
NPIP
PLA2G10
TEKT5
NPIP
NPIP
RRN3
MPV17L
C16orf45
NDE1
MARF1
Annotation Key
LCR16a-011 130 kbp
LCR16a-009 90 kbp
LCR16a-010 35 kbp
LCR16a-001a 24 kbp
LCR16a 9 kbp
LCR16a-020 40 kbp
LCR16a-017 36 kbp
B
Marmoset
chr16p13.13
100 kbp
SegDups
DupMasker
Genes
WSSD
ZC3H7A
TXNDC11
SNN
LITAF
NPIP
ENSCJAT01/RMI2
ENSCJAT02
SOCS1
PRM2
CLEC16A
Annotation Key
LCR16a-001 24 kbp
LCR16a-001a 106 kbp
LCR16a 9 kbp
ENSCJAT00000031210
ENSCJAT00000043972
C
Marmoset
chr16p13.13
100 kbp
SegDups
DupMasker
Genes
WSSD
ATF7IP2
EMP2
TEKT5
NPIP
Annotation Key
LCR16a-017 35 kbp
LCR16a-001a 40 kbp
LCR16a 9 kbp

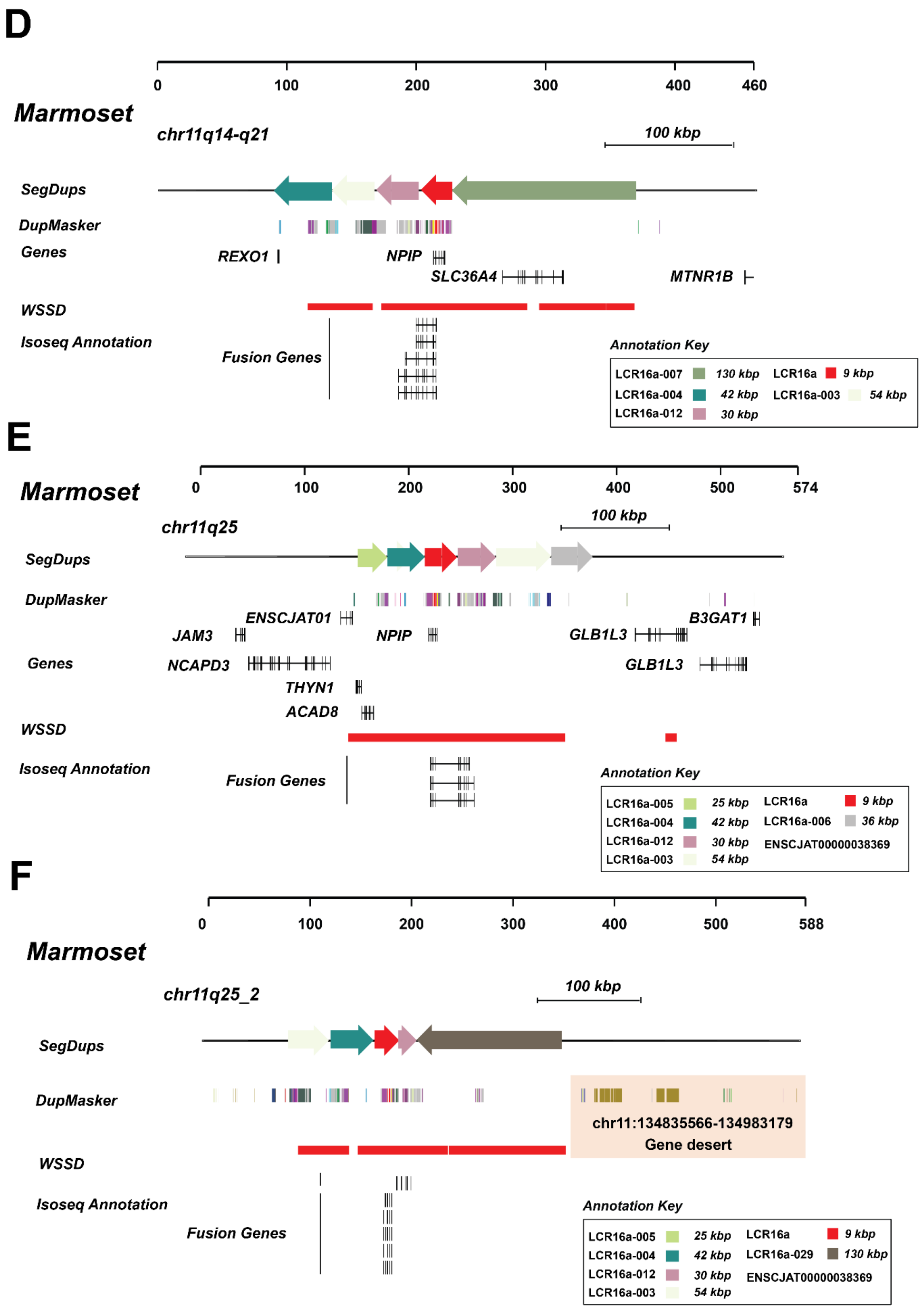
D
0
100
200
300
400
460
Marmoset
chr11q14-q21
100 kbp
SegDups
DupMasker
Genes
REXO1
NPIP
SLC36A4
MTNR1B
WSSD
Isoseq Annotation
Fusion Genes
Annotation Key
LCR16a-007 130 kbp
LCR16a 9 kbp
LCR16a-004 42 kbp
LCR16a-003 54 kbp
LCR16a-012 30 kbp
E
0
100
200
300
400
500
574
Marmoset
chr11q25
100 kbp
SegDups
DupMasker
ENSCJAT01
B3GAT1
JAM3
NPIP
GLB1L3
Genes
NCAPD3
GLB1L3
THYN1
ACAD8
WSSD
Isoseq Annotation
Fusion Genes
Annotation Key
LCR16a-005 25 kbp
LCR16a 9 kbp
LCR16a-004 42 kbp
LCR16a-006 36 kbp
LCR16a-012 30 kbp
ENSCJAT00000038369
LCR16a-003 54 kbp
F
0
100
200
300
400
500
588
Marmoset
chr11q25_2
100 kbp
SegDups
DupMasker
chr11:134835566-134983179
Gene desert
WSSD
Isoseq Annotation
Fusion Genes
Annotation Key
LCR16a-005 25 kbp
LCR16a 9 kbp
LCR16a-004 42 kbp
LCR16a-029 130 kbp
LCR16a-012 30 kbp
ENSCJAT00000038369
LCR16a-003 54 kbp

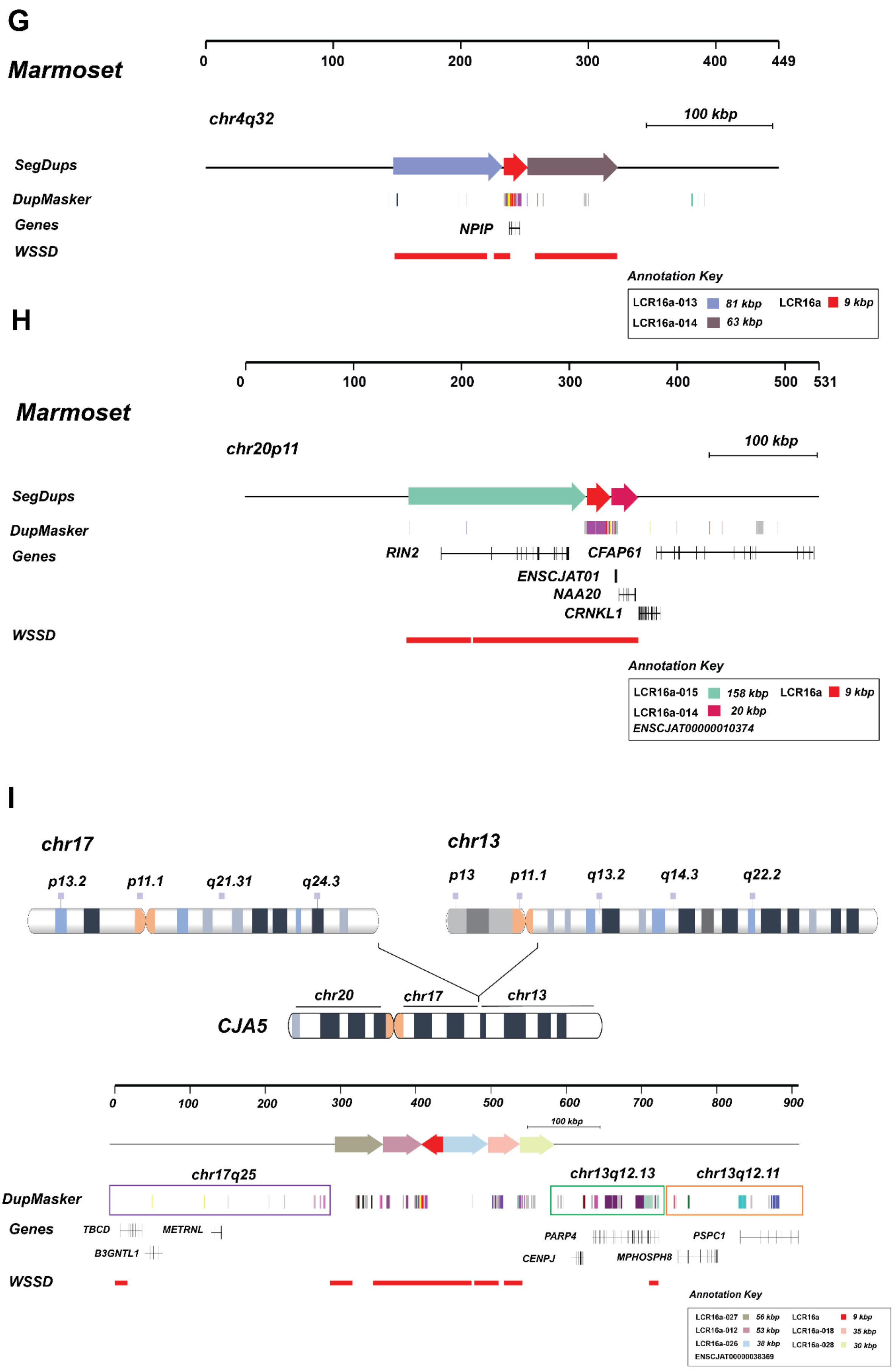
G
Marmoset
0 100 200 300 400 449
chr4q32
100 kbp
SegDups
DupMasker
Genes
NPIP
WSSD
Annotation Key
LCR16a-013 81 kbp
LCR16a 9 kbp
LCR16a-014 63 kbp
H
Marmoset
0 100 200 300 400 500 531
chr20p11
100 kbp
SegDups
DupMasker
Genes
RIN2
CFAP61
ENSCJAT01
NAA20
CRNKL1
WSSD
Annotation Key
LCR16a-015 158 kbp
LCR16a 9 kbp
LCR16a-014 20 kbp
ENSCJAT00000010374
I
chr17
p13.2 p11.1 q21.31 q24.3
chr13
p13 p11.1 q13.2 q14.3 q22.2
chr20 chr17 chr13
CJA5
0 100 200 300 400 500 600 700 800 900
100 kbp
chr17q25
chr13q12.13
chr13q12.11
DupMasker
Genes
TBCD
METRNL
B3GNTL1
PARP4
PSPC1
CENPJ
MPHOSPH8
WSSD
Annotation Key
LCR16a-027 56 kbp
LCR16a 9 kbp
LCR16a-012 53 kbp
LCR16a-018 35 kbp
LCR16a-026 38 kbp
LCR16a-028 30 kbp
ENSCJAT00000038369

Figure S1: Eleven LCR16a marmoset insertions anchored to the GRCh38 reference genome. The schematics represents the organization nine LCR16a marmoset insertions based on sequence and assembly of large-insert clones. Supercontigs (396–920 kbp) were created to span duplicated sequences and are anchored in unique sequence (>20 kbp). Segmental duplication (SD) organization is depicted using coloured arrows; regions of increased read depth (WSSD) identify duplicated regions (red bars). Gene models are predicted using GMAP based on Iso-Seq and Ensembl transcript data. Additional annotations include DupMasker. The chromosomal map location of the insertions (GRCH38 coordinates) map to **A)** 16p13.12, **B)** 16p13.13, **C)** 16p13.13, **D)** 11q14, **E)** 11q25, **F)** 11q25, **G)** 4q32, **H)** 20p11, and **I)** 17q25_13q14.

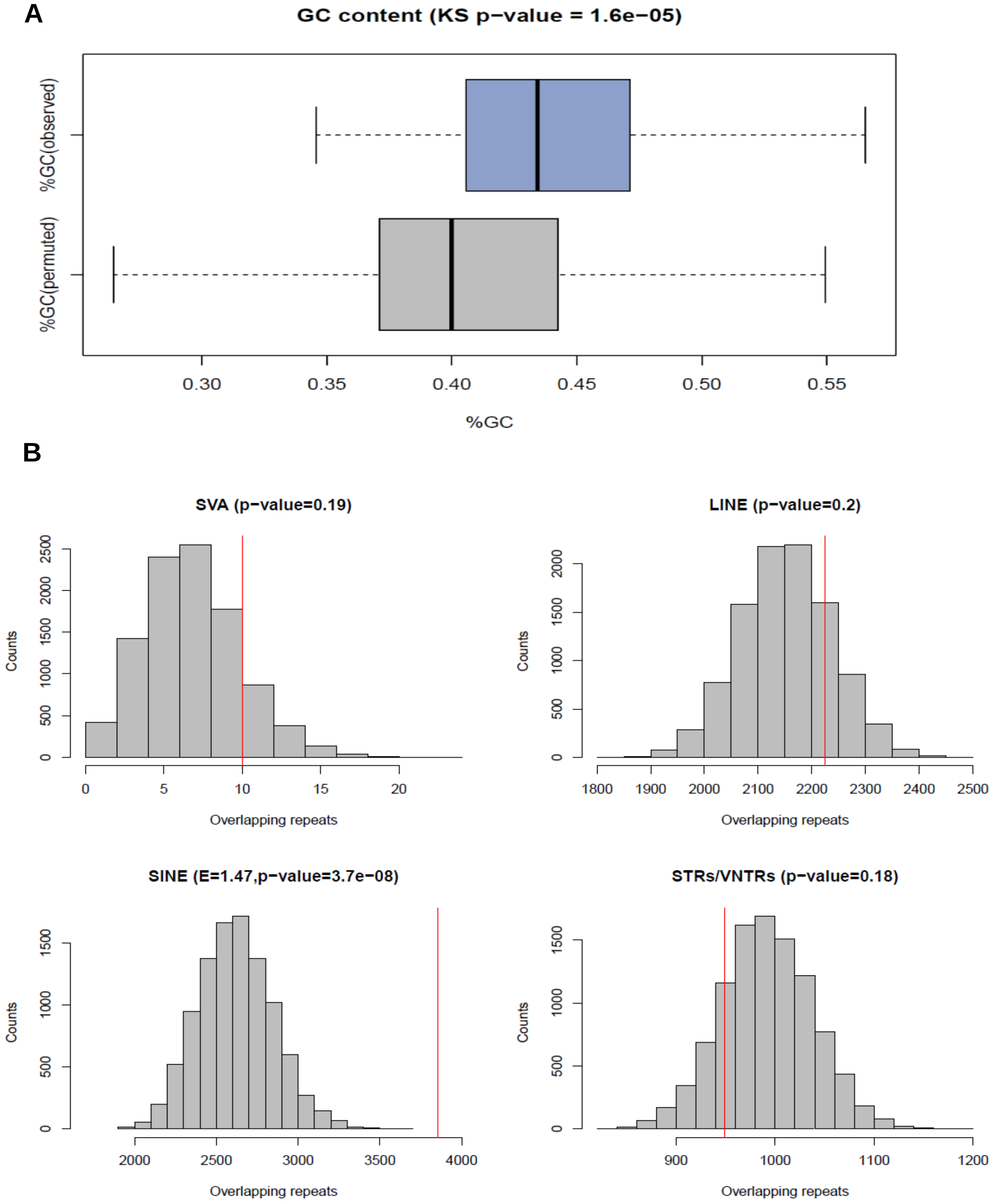
A
GC content (KS p−value = 1.6e−05)
%GC(observed)
%GC(permuted)
0.30
0.35
0.40
0.45
0.50
0.55
%GC
B
SVA (p−value=0.19)
Counts
Overlapping repeats
LINE (p−value=0.2)
Counts
Overlapping repeats
SINE (E=1.47,p−value=3.7e−08)
Counts
Overlapping repeats
STRs/VNTRs (p−value=0.18)
Counts
Overlapping repeats

C

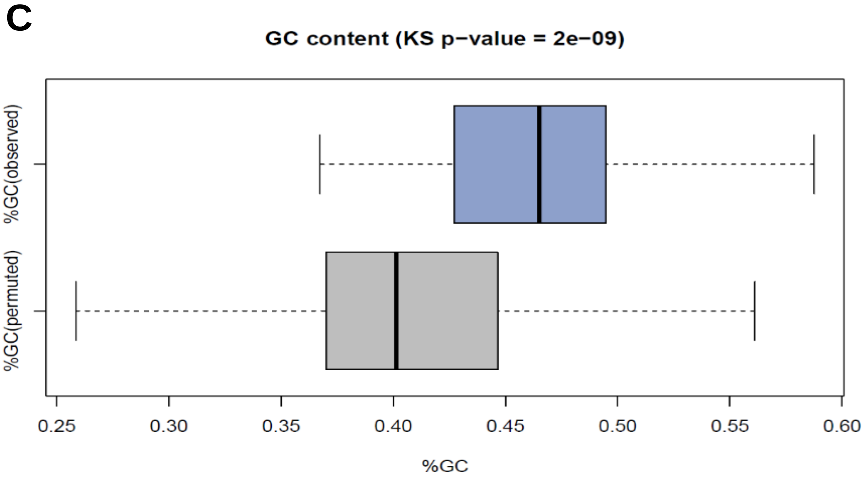

GC content (KS p−value = 2e−09)
%GC(observed)
%GC(permuted)
0.25
0.30
0.35
0.40
0.45
0.50
0.55
0.60
%GC

D

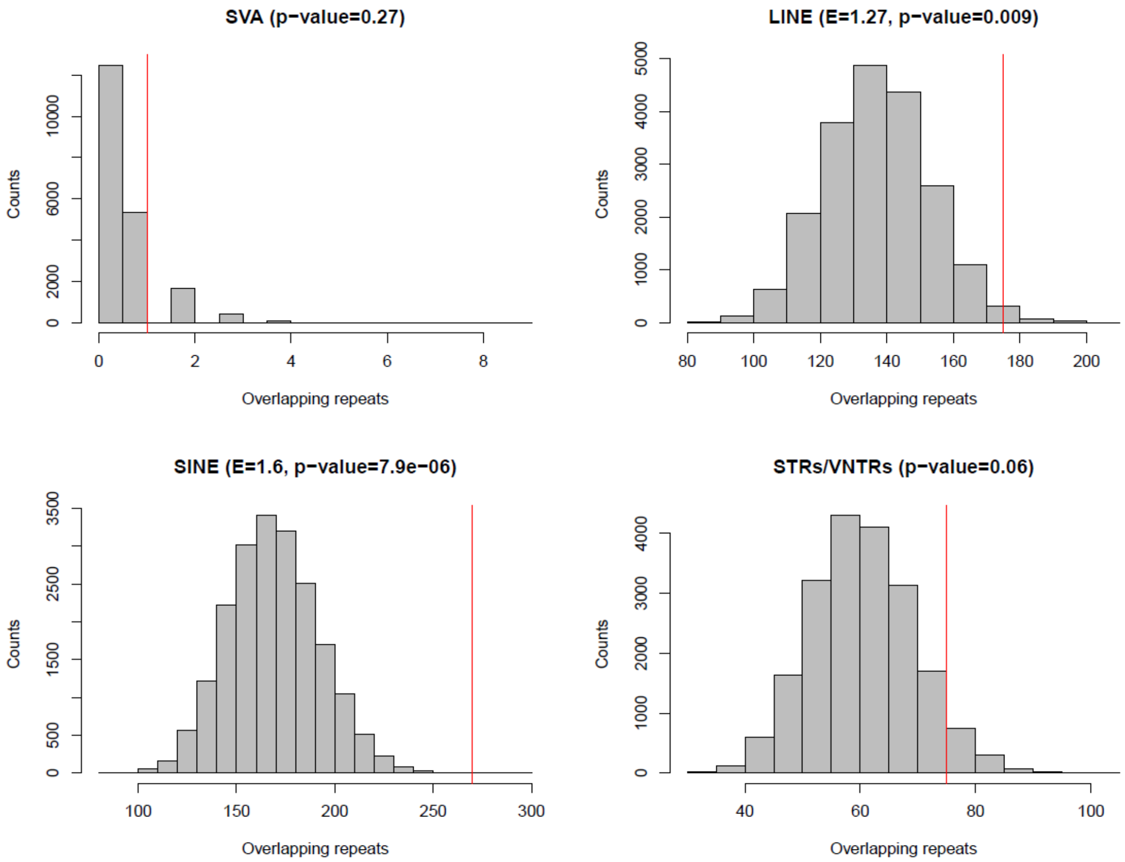

SVA (p−value=0.27)
Counts
0
2000
6000
10000
0
2
4
6
8
Overlapping repeats
LINE (E=1.27, p−value=0.009)
Counts
0
1000
2000
3000
4000
5000
80
100
120
140
160
180
200
Overlapping repeats
SINE (E=1.6, p−value=7.9e−06)
Counts
0
500
1500
2500
3500
100
150
200
250
300
Overlapping repeats
STRs/VNTRs (p−value=0.06)
Counts
0
1000
2000
3000
4000
40
60
80
100
Overlapping repeats

E

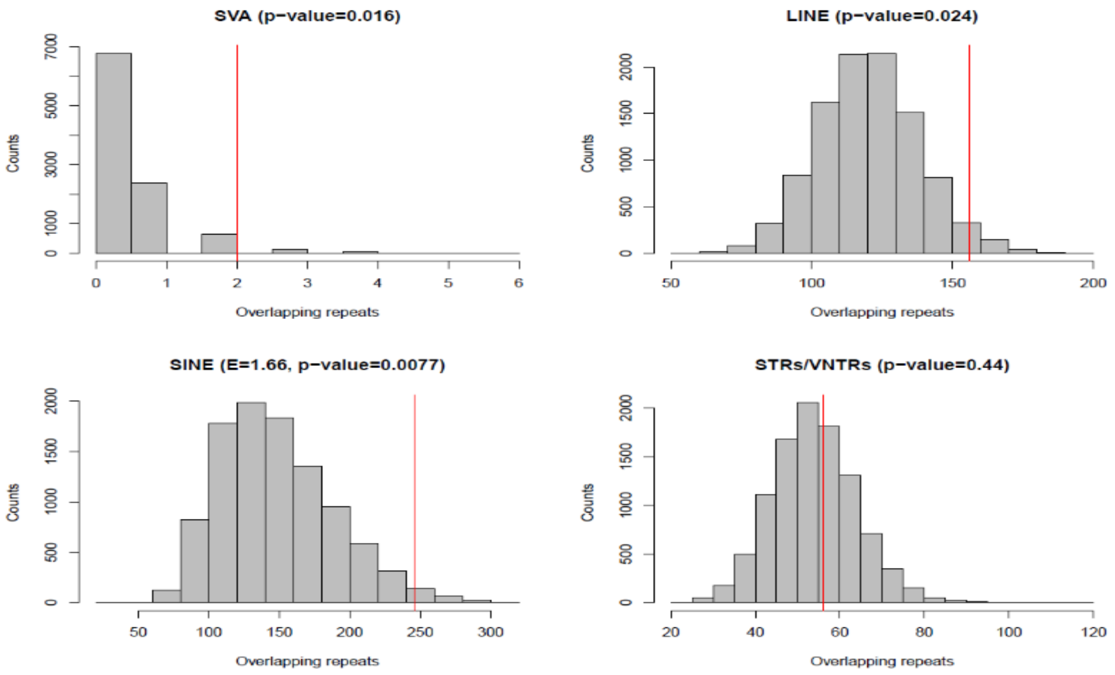

Figure S2: Sequence properties and enrichment analysis of donor and acceptor regions in association with LCR16a. **A)** GC content is elevated among donor duplication sites (n=63) compared to the null distribution (10,000 permutations). **B)** Analysis of common repeat content in donor duplication sites shows a relative enrichment of SINE elements compared to the null distribution. No evidence of SVA, LINE, or VNTR enrichment is identified at these sites. **C)** GC content is elevated at acceptor duplication sites (n=27) compared to the null distribution (10,000 permutations). **D)** Acceptor sites show a relative enrichment of SINE and LINE elements compared to the null distribution. **E)** LCR16a integration sites (n=13) show a relative enrichment of SINE elements compared to the null distribution, consistent with sequence resolved breakpoints among primates.

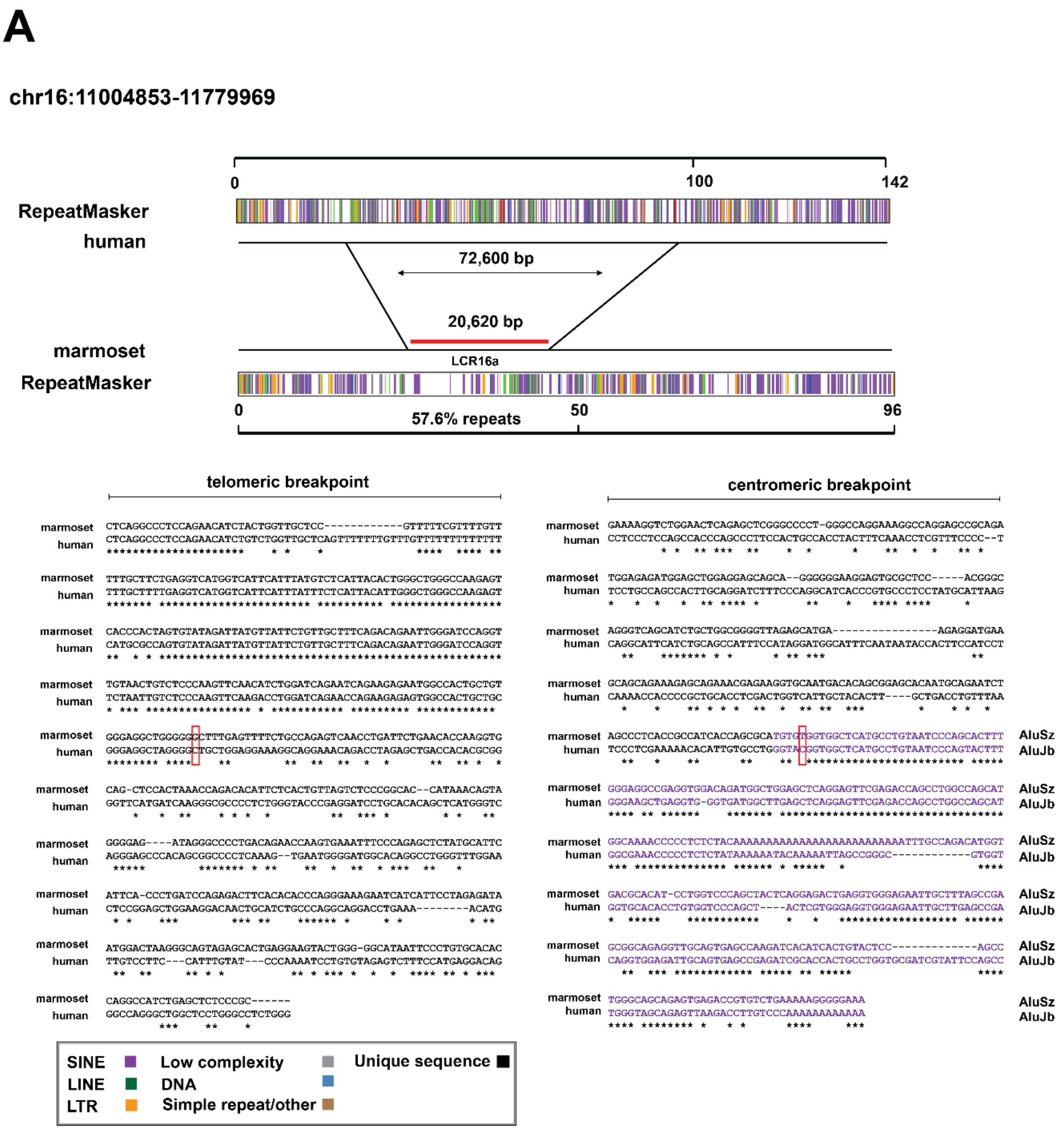
A
chr16:11004853-11779969
RepeatMasker
human
0
100
142
72,600 bp
20,620 bp
marmoset
LCR16a
RepeatMasker
0
50
96
57.6% repeats
telomeric breakpoint
centromeric breakpoint
marmoset
human
AluSz
AluJb
SINE
LINE
LTR
Low complexity
DNA
Simple repeat/other
Unique sequence

B

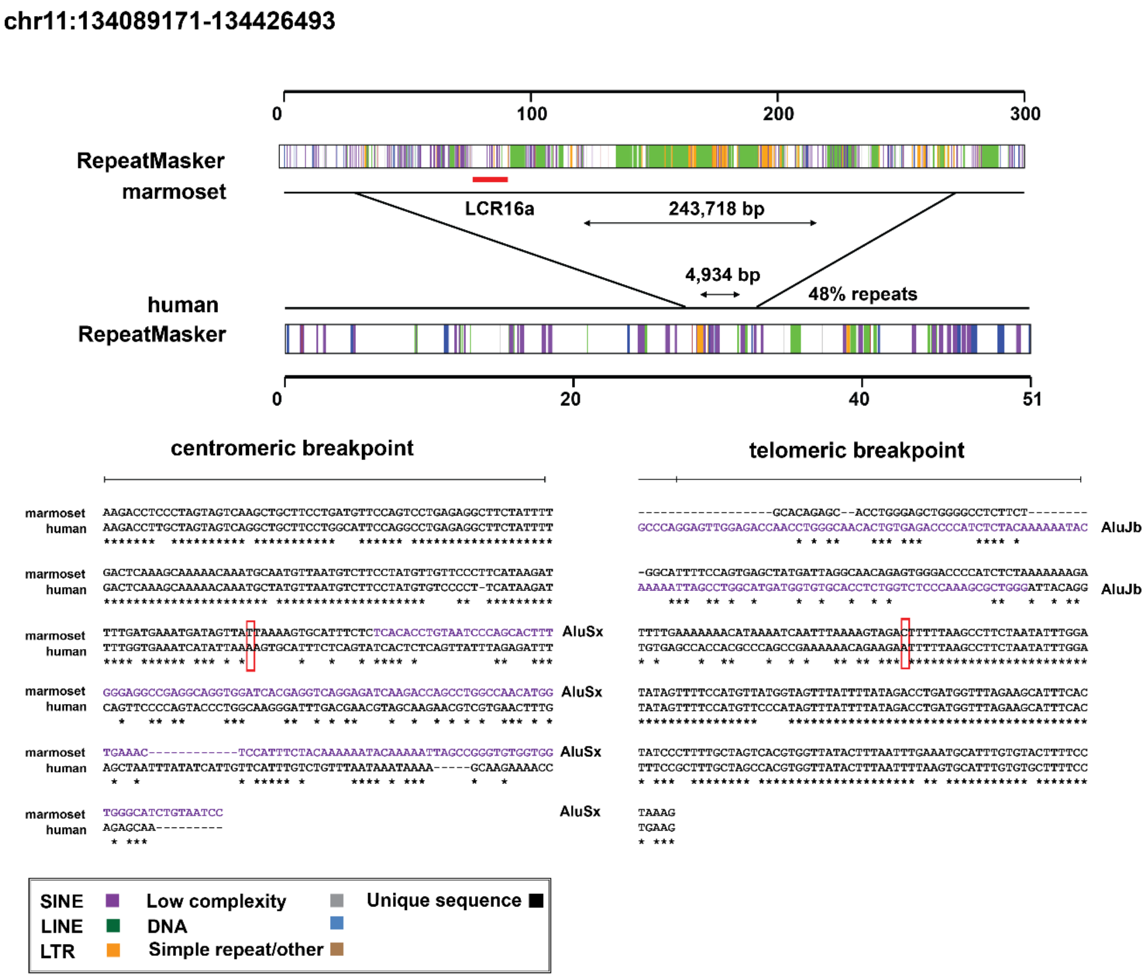
chr11:134089171-134426493
0
100
200
300
RepeatMasker
marmoset
LCR16a
243,718 bp
4,934 bp
48% repeats
human
RepeatMasker
0
20
40
51
centromeric breakpoint
telomeric breakpoint
marmoset
human
AluSx
AluJb
SINE
LINE
LTR
Low complexity
DNA
Simple repeat/other
Unique sequence

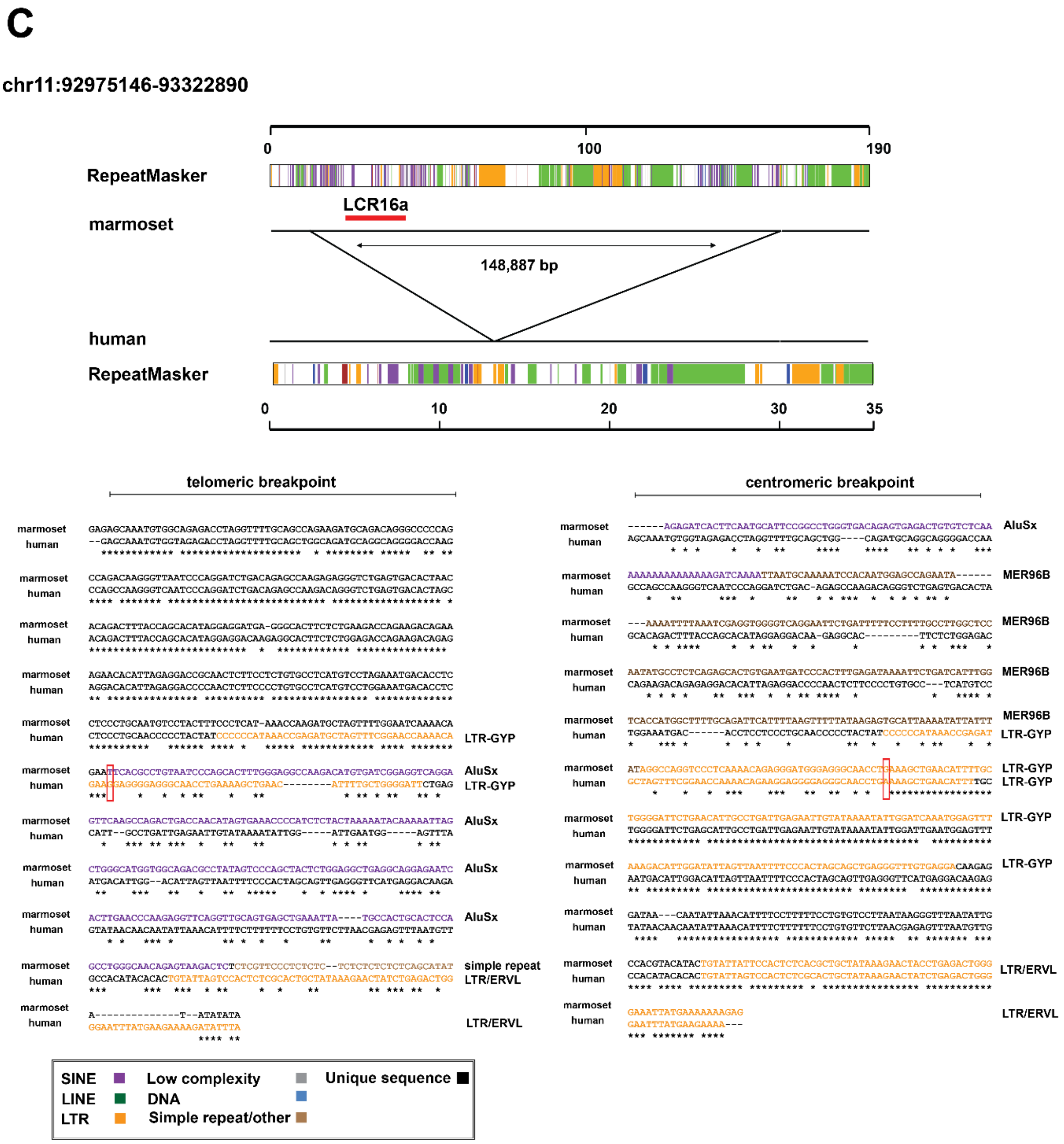
C
chr11:92975146-93322890
0
100
190
RepeatMasker
LCR16a
marmoset
148,887 bp
human
RepeatMasker
0
10
20
30
35
telomeric breakpoint
centromeric breakpoint
LTR-GYP
AluSx
LTR-GYP
AluSx
AluSx
AluSx
simple repeat
LTR/ERVL
LTR/ERVL
AluSx
MER96B
MER96B
MER96B
MER96B
LTR-GYP
LTR-GYP
LTR-GYP
LTR-GYP
LTR-GYP
LTR/ERVL
LTR/ERVL
SINE
LINE
LTR
Low complexity
DNA
Simple repeat/other
Unique sequence

D

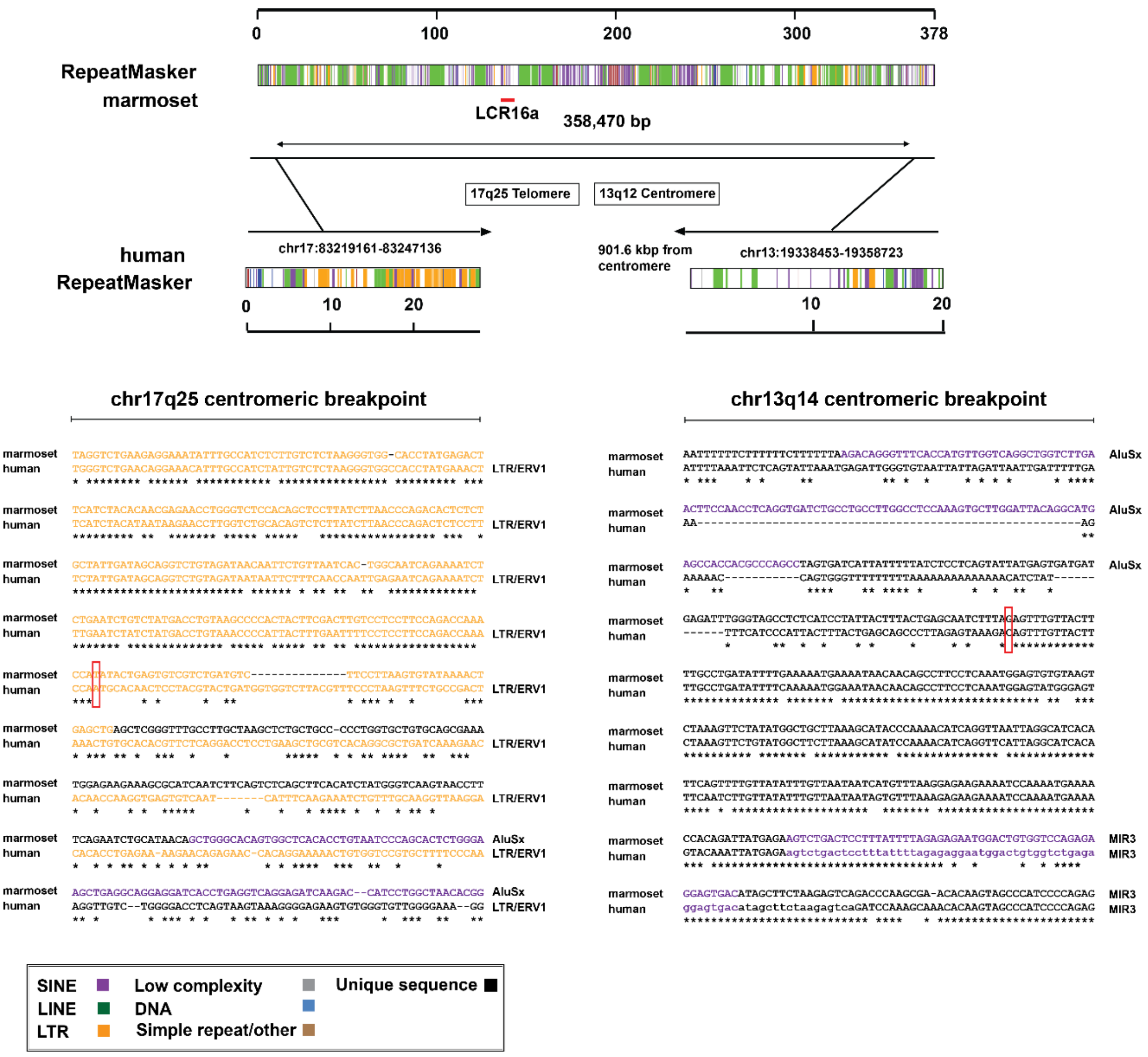
0
100
200
300
378
RepeatMasker
marmoset
LCR16a
358,470 bp
17q25 Telomere
13q12 Centromere
human
RepeatMasker
chr17:83219161-83247136
901.6 kbp from centromere
chr13:19338453-19358723
0
10
20
10
20
chr17q25 centromeric breakpoint
chr13q14 centromeric breakpoint
marmoset
human
LTR/ERV1
AluSx
MIR3
SINE
LINE
LTR
Low complexity
DNA
Simple repeat/other
Unique sequence

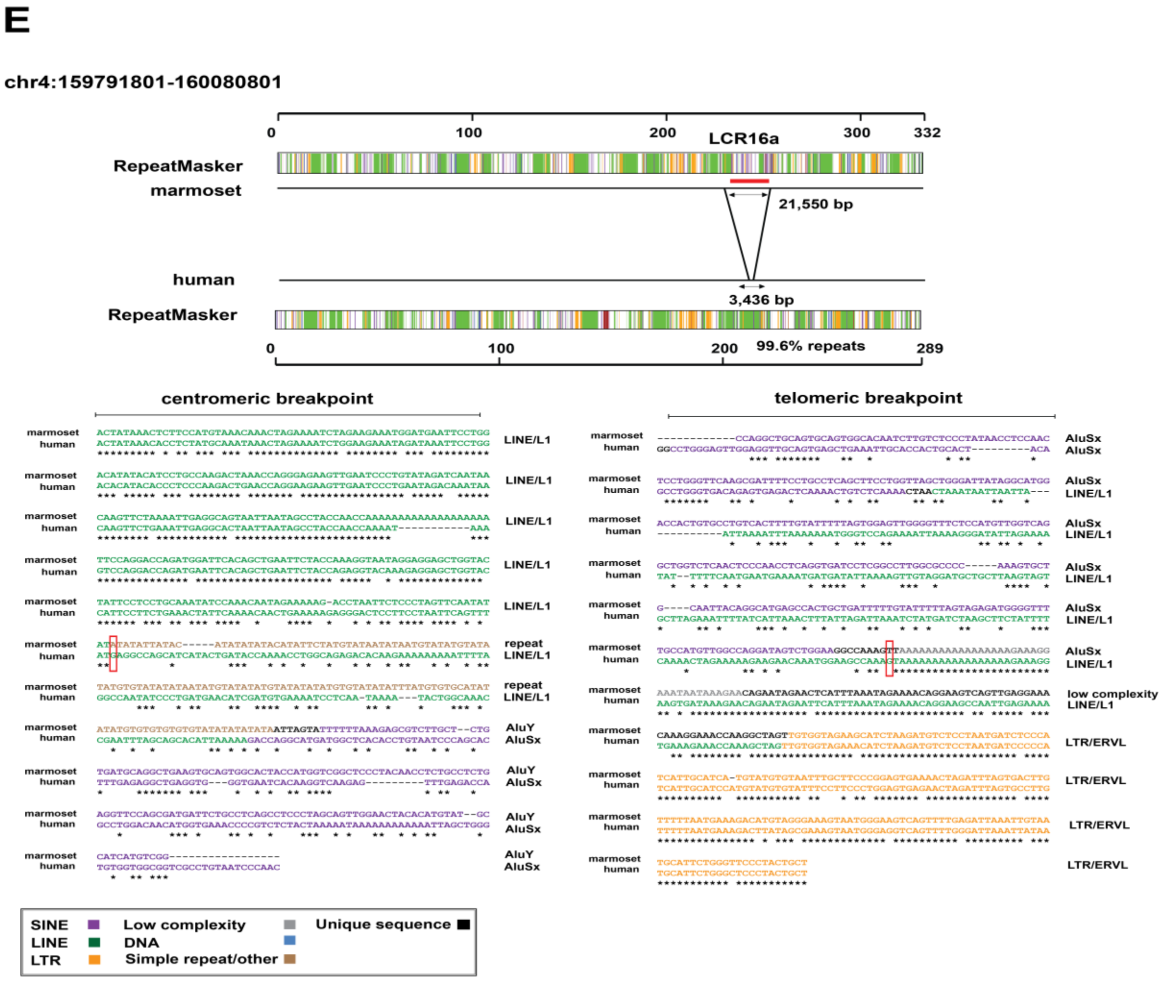

E
chr4:159791801-160080801
0
100
200
300
332
LCR16a
RepeatMasker
marmoset
21,550 bp
human
3,436 bp
RepeatMasker
0
100
200
99.6% repeats
289
centromeric breakpoint
telomeric breakpoint
LINE/L1
repeat
AluY
AluSx
AluSx
low complexity
LTR/ERVL
SINE
LINE
LTR
Low complexity
DNA
Simple repeat/other
Unique sequence

F

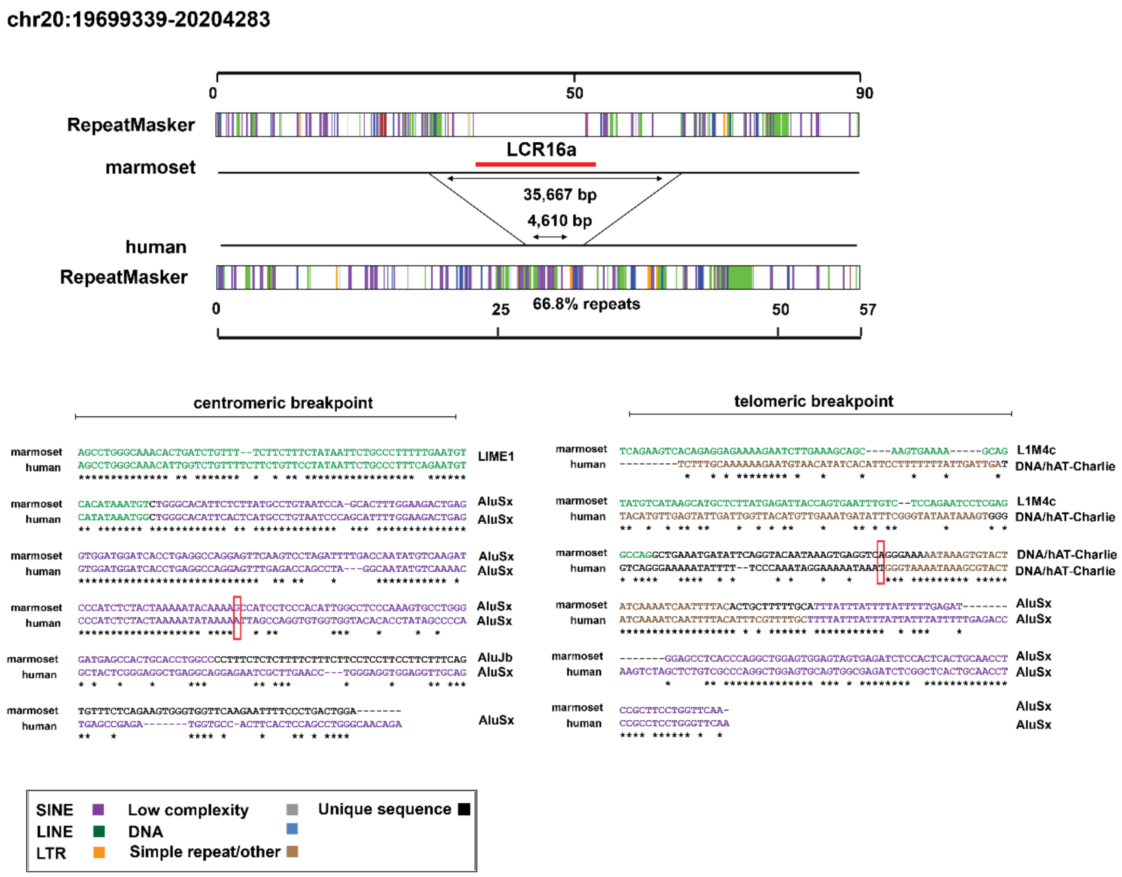
chr20:19699339-20204283
0
50
90
RepeatMasker
LCR16a
marmoset
35,667 bp
4,610 bp
human
RepeatMasker
0
25
66.8% repeats
50
57
centromeric breakpoint
telomeric breakpoint
marmoset
human
LIME1
AluSx
AluJb
L1M4c
DNA/hAT-Charlie
SINE
LINE
LTR
Low complexity
DNA
Simple repeat/other
Unique sequence

G

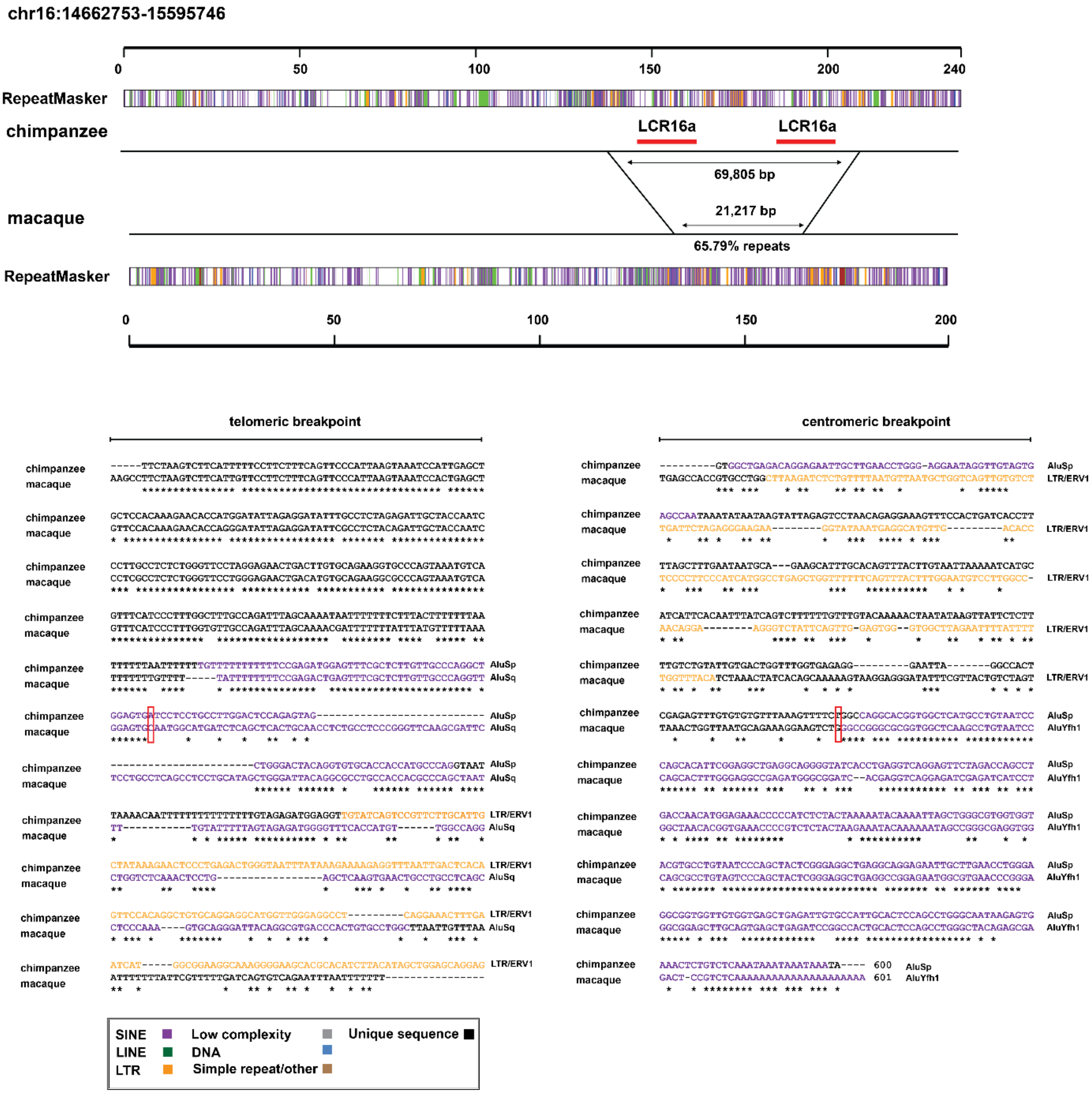

chr16:14662753-15595746
0
50
100
150
200
240
RepeatMasker
chimpanzee
LCR16a
LCR16a
69,805 bp
macaque
21,217 bp
65.79% repeats
RepeatMasker
0
50
100
150
200
telomeric breakpoint
centromeric breakpoint
chimpanzee
macaque
AluSp
AluSq
LTR/ERV1
AluYfh1
SINE
LINE
LTR
Low complexity
DNA
Simple repeat/other
Unique sequence

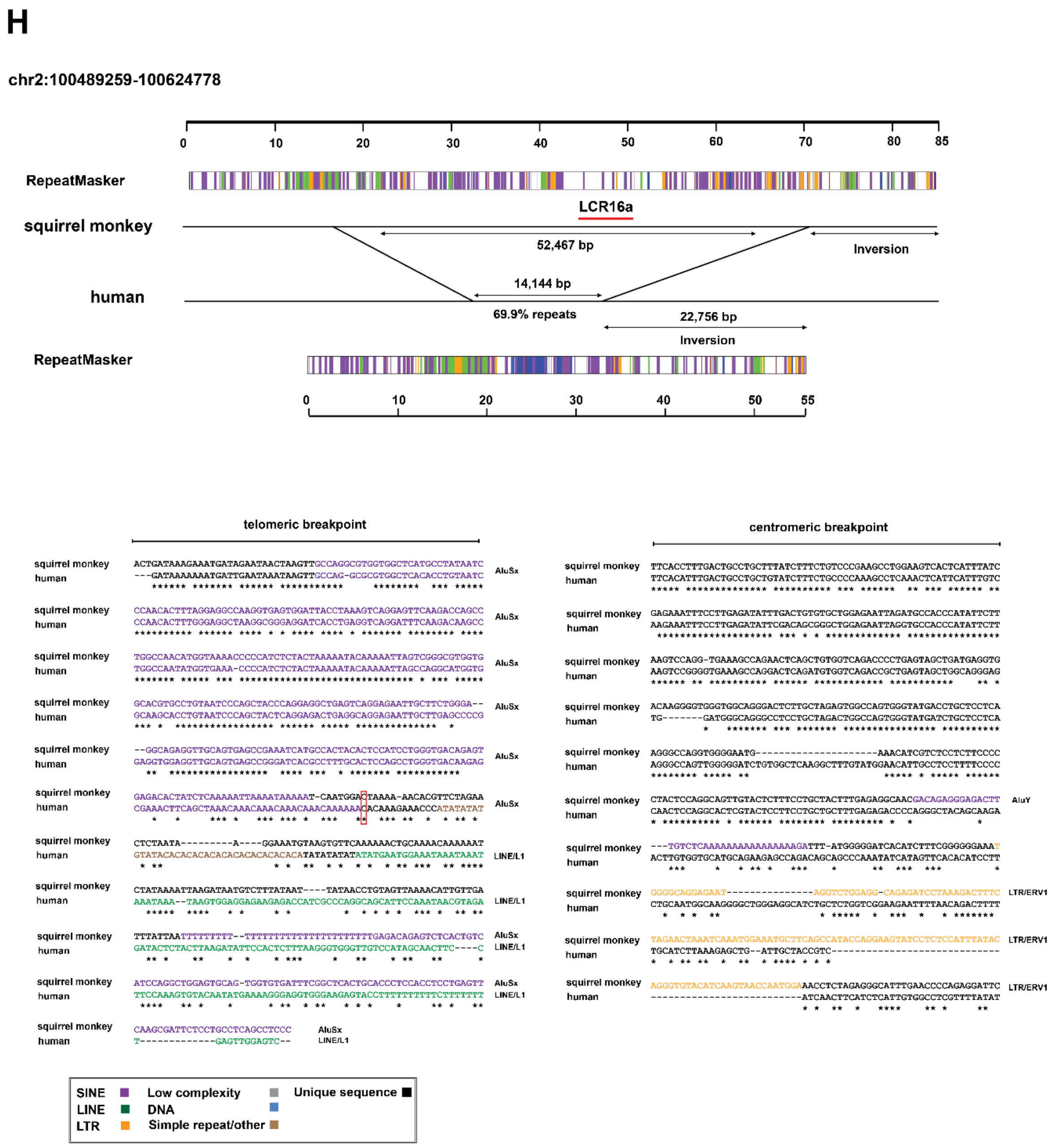

Figure S3: Breakpoint resolution from eight LCR16a insertions. The schematic depicts eight SD insertions corresponding to six marmoset (**A-F**), one chimpanzee (**G**), and one squirrel monkey (**H**). The chromosomal map location of the insertions (GRCH38 coordinates) map to **A)** 16p13.13, **B)** 11q25, **C)** 11q14, **D)** 17q25_13q14, **E)** 4q32, **F)** 20p11, **G)** 16p13, and **H)** 2q11. All comparisons were made against GRCh38, except in panel (**G**) whereby the chimpanzee assembly was compared to a custom sequence contig derived from the macaque CH250 BAC library. Starting from the top, tracks correspond to RepeatMasker annotation, size of the duplication insertion, LCR16a location, and corresponding sequence deleted from the insertion site. Pairwise sequence alignments taken upstream and downstream of the telomeric and centromeric breakpoints are depicted, with common repeats annotated at the breakpoints (red box). 64% of LCR16a-associated insertions map to an AluS element and in all but one case (**C**) where we find coordinated deletion of repeat-rich sequence (average 67.56%) ranging from 3.4 to 72.6 kbp in length.

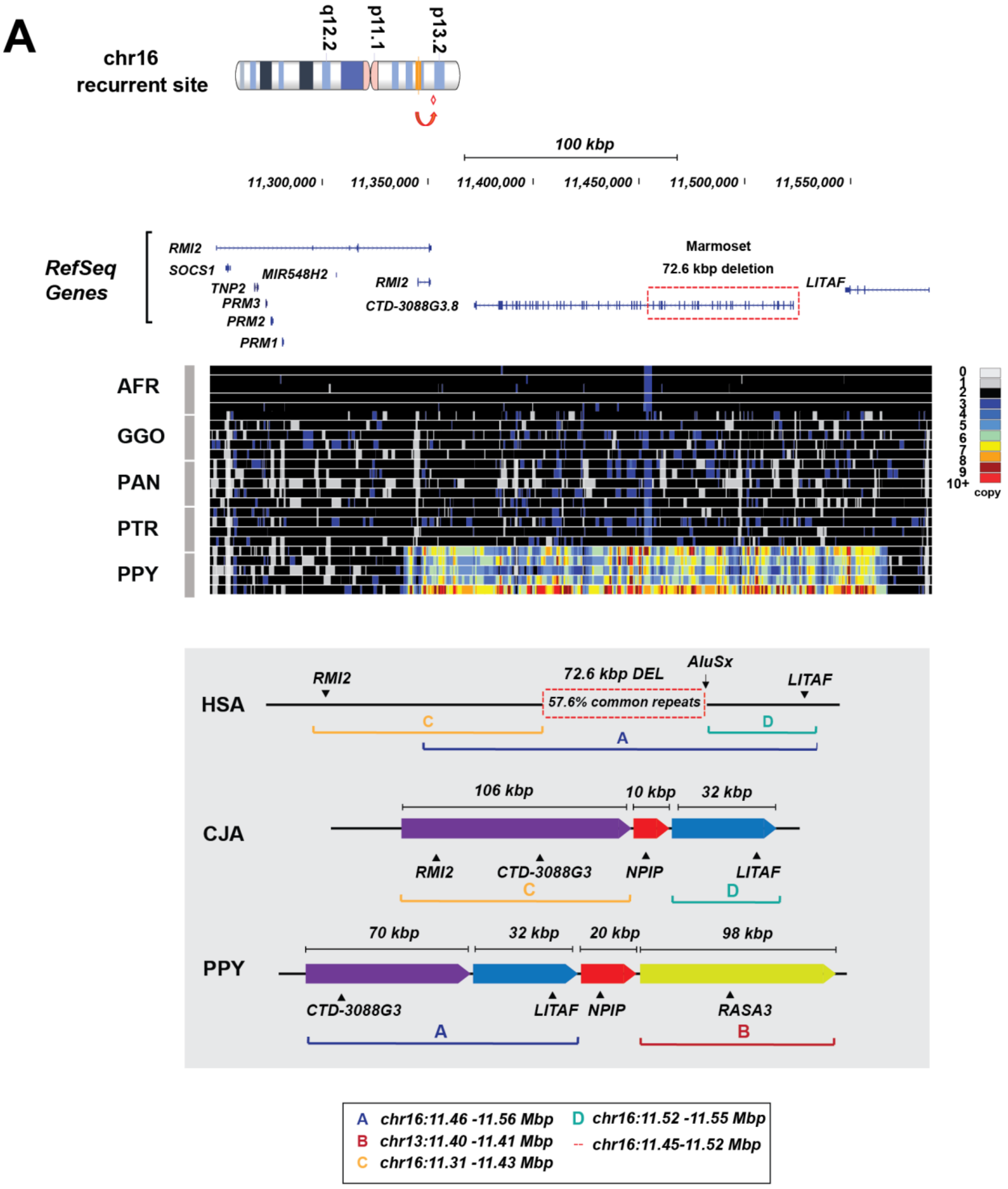
A
chr16
recurrent site
q12.2
p11.1
p13.2
100 kbp
11,300,000
11,350,000
11,400,000
11,450,000
11,500,000
11,550,000
RefSeq Genes
RMI2
SOCS1
MIR548H2
TNP2
PRM3
PRM2
PRM1
RMI2
CTD-3088G3.8
Marmoset
72.6 kbp deletion
LITAF
AFR
GGO
PAN
PTR
PPY
0
1
2
3
4
5
6
7
8
9
10+
copy
HSA
RMI2
72.6 kbp DEL
AluSx
LITAF
57.6% common repeats
C
D
A
CJA
106 kbp
10 kbp
32 kbp
RMI2
CTD-3088G3
NPIP
LITAF
C
D
PPY
70 kbp
32 kbp
20 kbp
98 kbp
CTD-3088G3
LITAF
NPIP
RASA3
A
B
A chr16:11.46 -11.56 Mbp
B chr13:11.40 -11.41 Mbp
C chr16:11.31 -11.43 Mbp
D chr16:11.52 -11.55 Mbp
-- chr16:11.45-11.52 Mbp

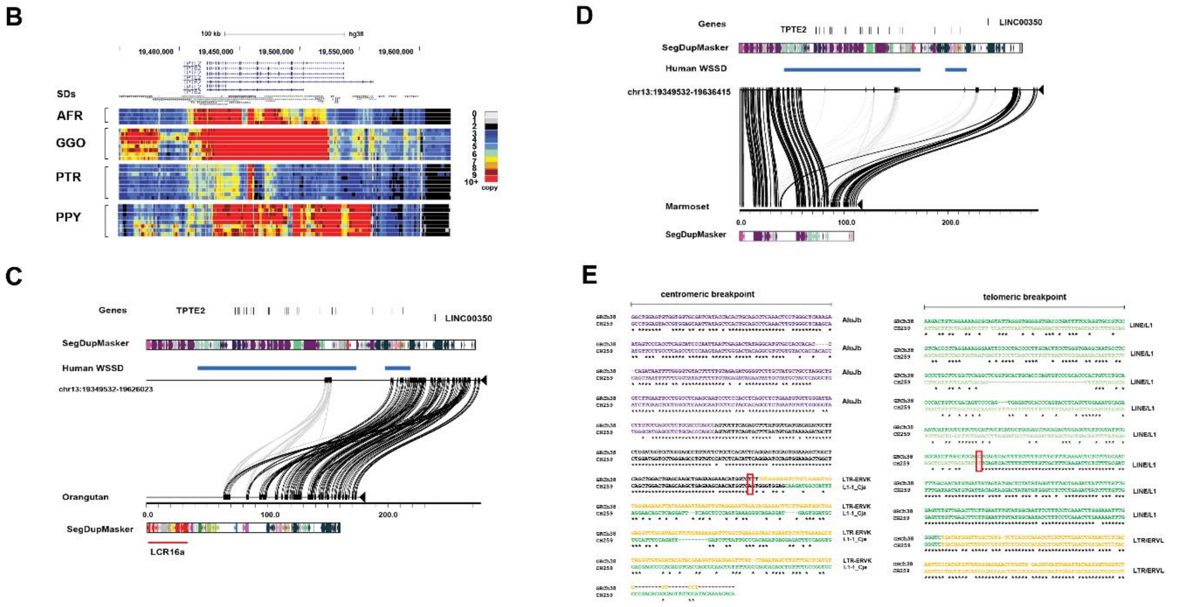

Figure S4: Evidence of recurrent LCR16a duplication during primate evolution. A) A UCSC Genome Browser snapshot of a recurrent LCR16a duplication block on chr16p13.13. Annotation tracks include RefSeq gene annotation and copy number (CN) heatmaps (CN index shown) produced from Illumina read-depth profiles from modern humans and nonhuman primates (NHPs). All orangutans carry a large >220 kbp duplication block, which includes the carboxy-terminus of *LITAF* and a long noncoding RNA LOC101927131. At the bottom, a schematic of the locus in human, marmoset, and orangutan is provided based on comparative analysis of large-insert clones. SDs were identified within the contigs of marmoset and orangutan shown as coloured arrows. The marmoset contains a 176.73 kbp duplication block, which includes duplications of *RMI2*, *ENST00000598234.5*, and *LITAF*. The orangutan has a larger >220 kbp duplication block that includes *ENST00000598234.5*, *LITAF*, and a gene from 13q24, *RASA3*. All of these duplications in marmoset and orangutan are in association with LCR16a. **B)** A UCSC Genome Browser snapshot of a recurrent LCR16a duplication block on chr13q12.1. CN heatmaps show that this region has been subject to multiple rounds of rearrangement during ape evolution. The CN heatmaps include representations of AFR=African Humans, GGO=gorilla, PTR=chimpanzee, PPY=orangutan. **C)** A Miropeats comparison of the human and orangutan contigs shows the pairwise differences between the orthologous regions. Annotations include whole-genome shotgun sequence detection (WSSD) in human indicating duplicated regions identified by sequence read depth, DupMasker, and exons of genes. Miropeats identifies a bifurcated alignment that includes >80 kbp of orthologous sequence and a >100 kbp of duplication block absent from the human assembly adjacent to LCR16a. **D)** A Miropeats comparison of human and marmoset chr13q12.1 region shows a large ~208 kbp duplication block missing from the marmoset assembly that includes the ~80 kbp duplicate gene *TPTE2*. LCR16a is located ~210 kbp downstream of this site. **E)** Pairwise sequence alignment flanking the breakpoint of the ~80 kbp *TPTE2* deletion in marmoset shows an AluJb element at the centromeric breakpoint and a LINE/L1 element at the telomeric breakpoint.

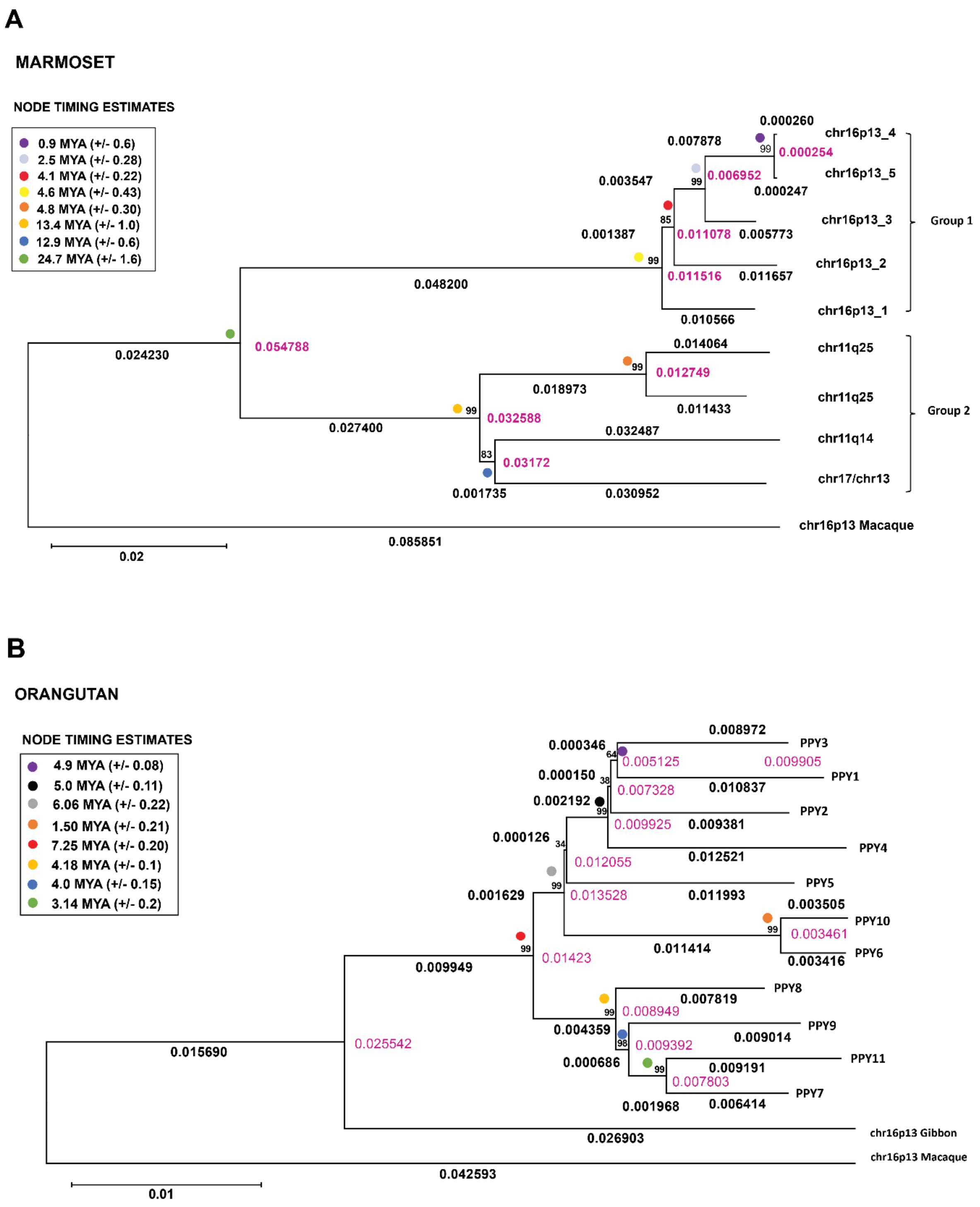

A
MARMOSET
NODE TIMING ESTIMATES
0.9 MYA (+/- 0.6)
2.5 MYA (+/- 0.28)
4.1 MYA (+/- 0.22)
4.6 MYA (+/- 0.43)
4.8 MYA (+/- 0.30)
13.4 MYA (+/- 1.0)
12.9 MYA (+/- 0.6)
24.7 MYA (+/- 1.6)
chr16p13_4
chr16p13_5
chr16p13_3
chr16p13_2
chr16p13_1
Group 1
chr11q25
chr11q25
chr11q14
chr17/chr13
Group 2
chr16p13 Macaque
0.000260
0.000254
0.000247
0.007878
0.006952
0.003547
0.011078
0.005773
0.001387
0.011516
0.011657
0.048200
0.010566
0.014064
0.012749
0.054788
0.024230
0.018973
0.011433
0.027400
0.032588
0.032487
0.03172
0.001735
0.030952
0.085851
0.02
B
ORANGUTAN
NODE TIMING ESTIMATES
4.9 MYA (+/- 0.08)
5.0 MYA (+/- 0.11)
6.06 MYA (+/- 0.22)
1.50 MYA (+/- 0.21)
7.25 MYA (+/- 0.20)
4.18 MYA (+/- 0.1)
4.0 MYA (+/- 0.15)
3.14 MYA (+/- 0.2)
PPY3
PPY1
PPY2
PPY4
PPY5
PPY10
PPY6
PPY8
PPY9
PPY11
PPY7
chr16p13 Gibbon
chr16p13 Macaque
0.008972
0.000346
0.005125
0.009905
0.000150
0.007328
0.010837
0.002192
0.009925
0.009381
0.000126
0.012055
0.012521
0.013528
0.011993
0.001629
0.003505
0.003461
0.011414
0.003416
0.01423
0.009949
0.007819
0.008949
0.004359
0.009014
0.009392
0.000686
0.009191
0.007803
0.001968
0.006414
0.015690
0.025542
0.026903
0.042593
0.01

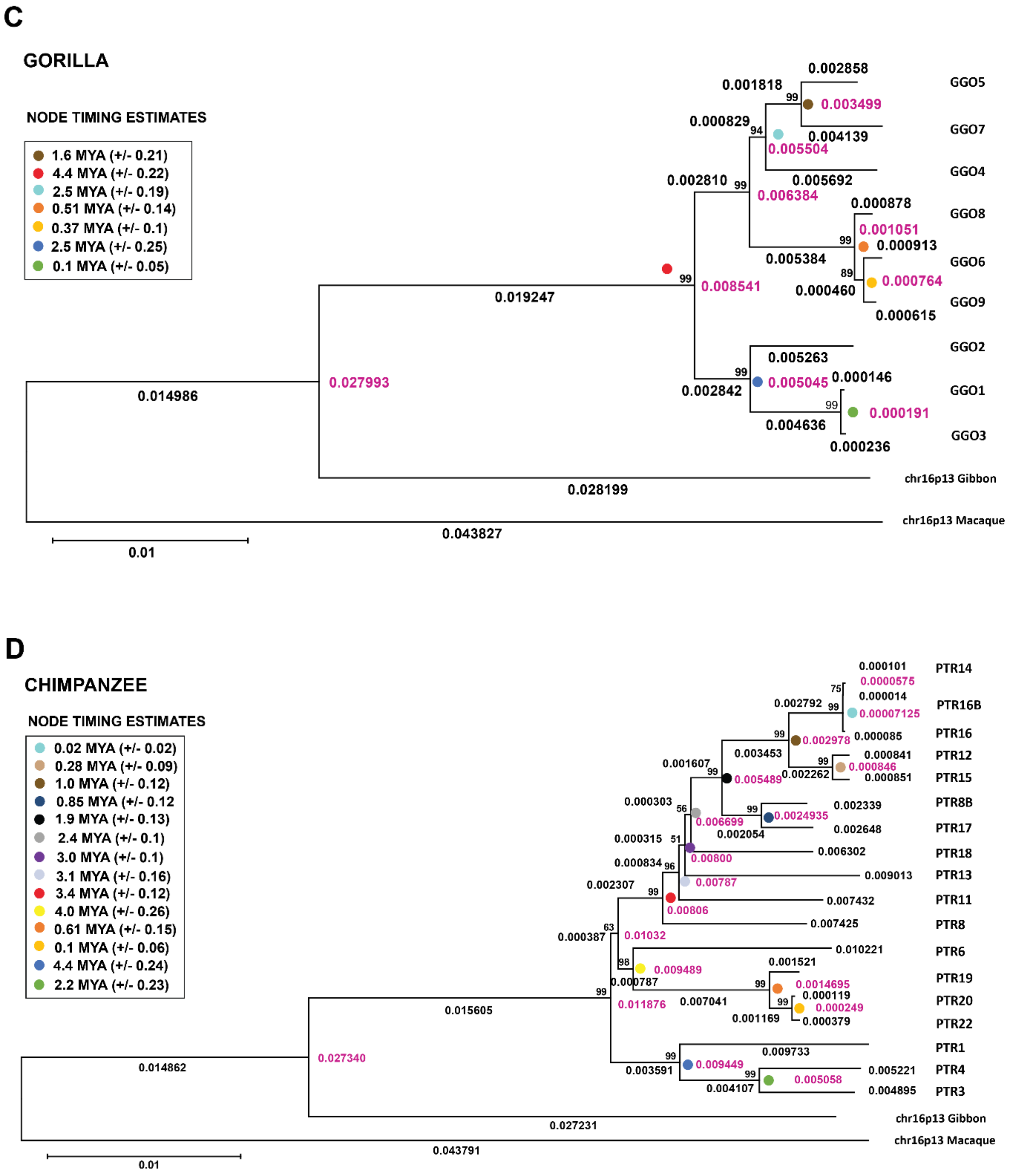
C
GORILLA
NODE TIMING ESTIMATES
1.6 MYA (+/- 0.21)
4.4 MYA (+/- 0.22)
2.5 MYA (+/- 0.19)
0.51 MYA (+/- 0.14)
0.37 MYA (+/- 0.1)
2.5 MYA (+/- 0.25)
0.1 MYA (+/- 0.05)
GGO5
GGO7
GGO4
GGO8
GGO6
GGO9
GGO2
GGO1
GGO3
chr16p13 Gibbon
chr16p13 Macaque
0.002858
0.001818
0.003499
0.000829
0.004139
0.005504
0.002810
0.005692
0.006384
0.000878
0.001051
0.000913
0.005384
0.000764
0.000460
0.000615
0.019247
0.008541
0.005263
0.005045
0.000146
0.002842
0.000191
0.004636
0.000236
0.014986
0.027993
0.028199
0.043827
0.01
D
CHIMPANZEE
NODE TIMING ESTIMATES
0.02 MYA (+/- 0.02)
0.28 MYA (+/- 0.09)
1.0 MYA (+/- 0.12)
0.85 MYA (+/- 0.12
1.9 MYA (+/- 0.13)
2.4 MYA (+/- 0.1)
3.0 MYA (+/- 0.1)
3.1 MYA (+/- 0.16)
3.4 MYA (+/- 0.12)
4.0 MYA (+/- 0.26)
0.61 MYA (+/- 0.15)
0.1 MYA (+/- 0.06)
4.4 MYA (+/- 0.24)
2.2 MYA (+/- 0.23)
PTR14
PTR16B
PTR16
PTR12
PTR15
PTR8B
PTR17
PTR18
PTR13
PTR11
PTR8
PTR6
PTR19
PTR20
PTR22
PTR1
PTR4
PTR3
chr16p13 Gibbon
chr16p13 Macaque
0.000101
0.0000575
0.000014
0.002792
0.00007125
0.002978
0.000085
0.003453
0.000841
0.001607
0.000846
0.005489
0.002262
0.000851
0.000303
0.002339
0.006699
0.0024935
0.002648
0.002054
0.000315
0.00800
0.006302
0.000834
0.00787
0.009013
0.002307
0.007432
0.00806
0.007425
0.000387
0.01032
0.010221
0.001521
0.009489
0.000787
0.0014695
0.000119
0.011876
0.007041
0.000249
0.001169
0.000379
0.015605
0.009733
0.009449
0.003591
0.005221
0.005058
0.004107
0.004895
0.014862
0.027340
0.027231
0.043791
0.01

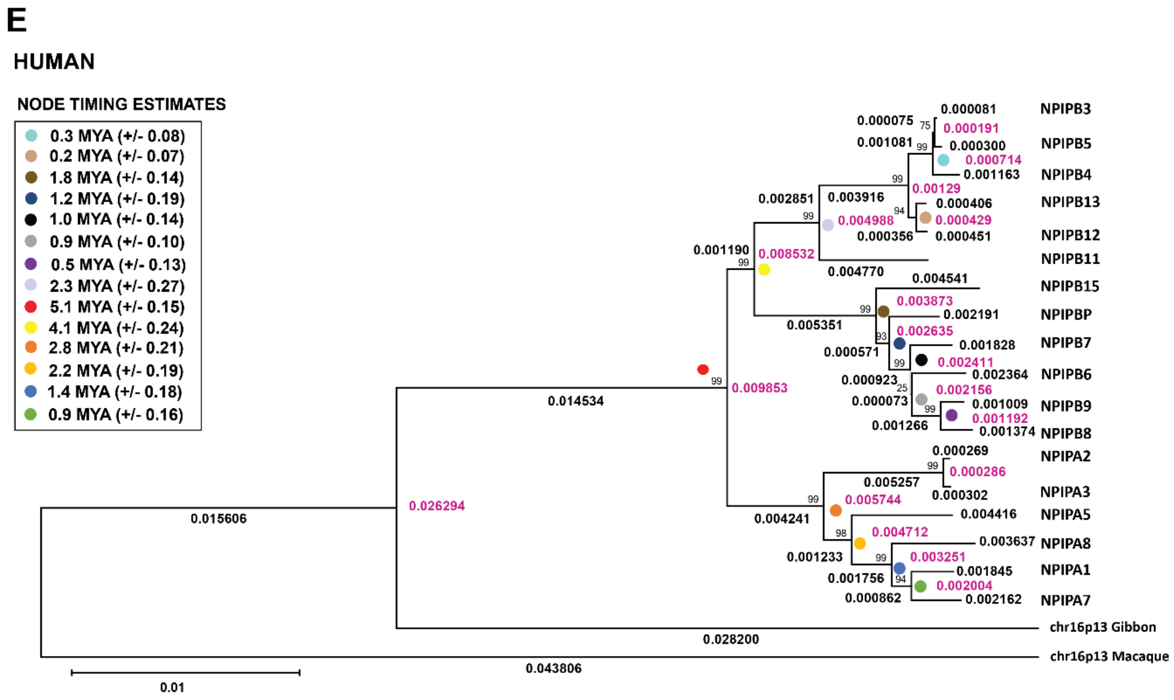

Figure S5: Evolutionary analysis and timing estimates of LCR16a copies in primates. An unrooted neighbour-joining tree was constructed using the MEGA5 complete deletion option based on ~9 kbp of aligned sequence representing LCR16a paralogs identified in marmoset (**A**), orangutan (**B**), gorilla (**C**), chimpanzee (**D**) and human (**E**), including the ancestral sequence in macaque as an outgroup. The evolutionary distances were computed using the Kimura 2-parameter method and timing estimates were performed using a divergence time of 25 mya for macaque and 35 mya for macaque/marmoset. Coloured dots represent the timing estimates of each node identified in the phylogeny.

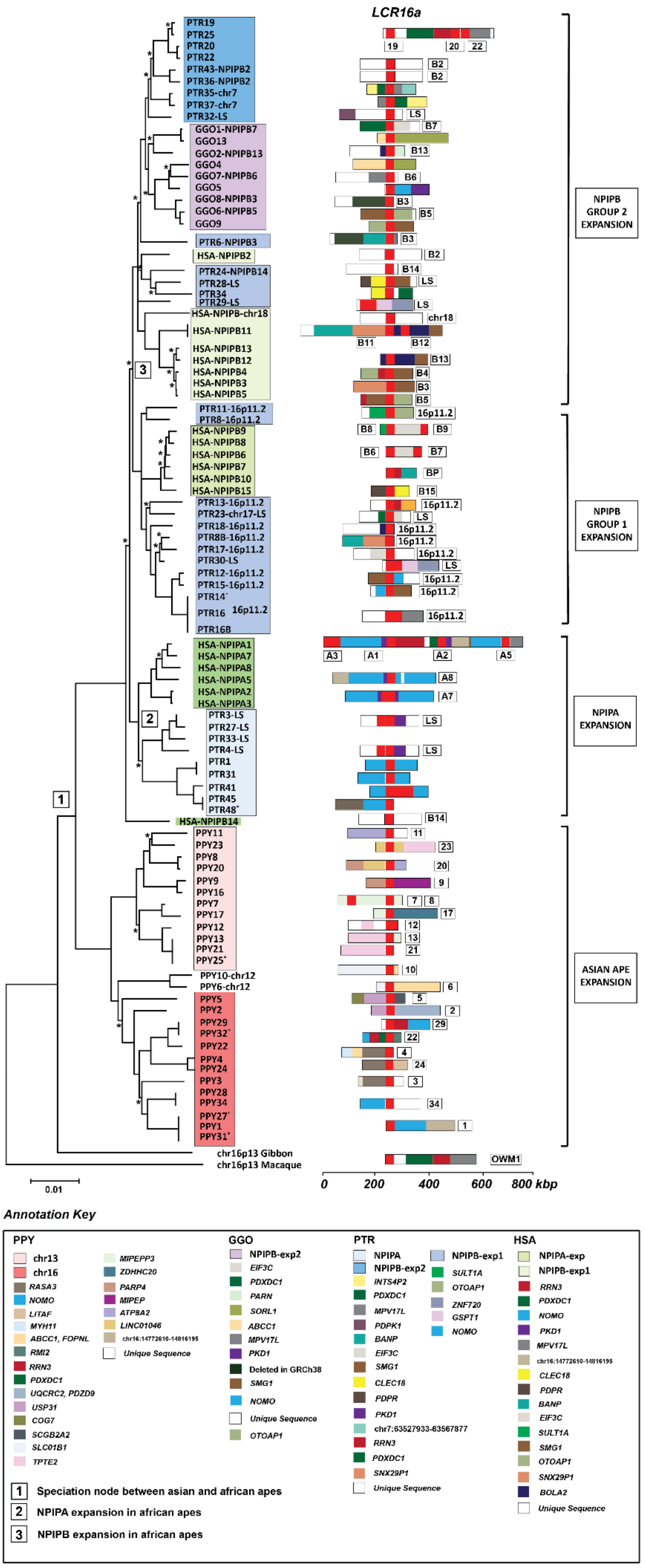

LCR16a
NPIPB GROUP 2 EXPANSION
NPIPB GROUP 1 EXPANSION
NPIPA EXPANSION
ASIAN APE EXPANSION
PTR19
PTR25
PTR20
PTR22
PTR43-NPIPB2
PTR36-NPIPB2
PTR35-chr7
PTR37-chr7
PTR32-LS
GGO1-NPIPB7
GGO13
GGO2-NPIPB13
GGO4
GGO7-NPIPB6
GGO5
GGO8-NPIPB3
GGO6-NPIPB5
GGO9
PTR6-NPIPB3
HSA-NPIPB2
PTR24-NPIPB14
PTR28-LS
PTR34
PTR29-LS
HSA-NPIPB-chr18
HSA-NPIPB11
HSA-NPIPB13
HSA-NPIPB12
HSA-NPIPB4
HSA-NPIPB3
HSA-NPIPB5
PTR11-16p11.2
PTR8-16p11.2
HSA-NPIPB9
HSA-NPIPB8
HSA-NPIPB6
HSA-NPIPB7
HSA-NPIPB10
HSA-NPIPB15
PTR13-16p11.2
PTR23-chr17-LS
PTR18-16p11.2
PTR8B-16p11.2
PTR17-16p11.2
PTR30-LS
PTR12-16p11.2
PTR15-16p11.2
PTR14
PTR16 16p11.2
PTR16B
HSA-NPIPA1
HSA-NPIPA7
HSA-NPIPA8
HSA-NPIPA5
HSA-NPIPA2
HSA-NPIPA3
PTR3-LS
PTR27-LS
PTR33-LS
PTR4-LS
PTR1
PTR31
PTR41
PTR45
PTR48
HSA-NPIPB14
PPY11
PPY23
PPY8
PPY20
PPY9
PPY16
PPY7
PPY17
PPY12
PPY13
PPY21
PPY25
PPY10-chr12
PPY6-chr12
PPY5
PPY2
PPY29
PPY32
PPY22
PPY4
PPY24
PPY3
PPY28
PPY34
PPY27
PPY1
PPY31
chr16p13 Gibbon
chr16p13 Macaque
0.01
OWM1
0
200
400
600
800 kbp
Annotation Key
PPY
chr13
chr16
RASA3
NOMO
LITAF
MYH11
ABCC1, FOPNL
RMI2
RRN3
PDXDC1
UQCRC2, PDZD9
USP31
COG7
SCGB2A2
SLC01B1
TPTE2
MIPEPP3
ZDHHC20
PARP4
MIPEP
ATP8A2
LINC01046
chr16:14772610-14816195
Unique Sequence
GGO
NPIPB-exp2
EIF3C
PDXDC1
PARN
SORL1
ABCC1
MPV17L
PKD1
Deleted in GRCh38
SMG1
NOMO
Unique Sequence
OTOAP1
PTR
NPIPA
NPIPB-exp2
INTS4P2
PDXDC1
MPV17L
PDPK1
BANP
EIF3C
SMG1
CLEC18
PDPR
PKD1
chr7:63527933-63567877
RRN3
PDXDC1
SNX29P1
Unique Sequence
NPIPB-exp1
SULT1A
OTOAP1
ZNF720
GSPT1
NOMO
HSA
NPIPA-exp
NPIPB-exp1
RRN3
PDXDC1
NOMO
PKD1
MPV17L
chr16:14772610-14816195
CLEC18
PDPR
BANP
EIF3C
SULT1A
SMG1
OTOAP1
SNX29P1
BOLA2
Unique Sequence
1 Speciation node between asian and african apes
2 NPIPA expansion in african apes
3 NPIPB expansion in african apes

Figure S6: Evolutionary analysis of LCR16a copies in great apes. An unrooted neighbour-joining tree was constructed using the MEGA5 complete deletion option based on ~6 kbp of aligned sequence representing 86 LCR16a paralogs identified in the great ape lineage (human = HSA, chimpanzee = PTR, gorilla = GGO, PPY = orangutan). The ancestral sequence in macaque is used as an outgroup and evolutionary distances were computed using the Kimura 2-parameter method. Mosaic duplication block architecture is represented by coloured blocks and individual duplicons are referred to based on their gene content. HSA LCR16a copies are identified based on RefSeq nomenclature. Key nodes defining independent LCR16a clades are defined by boxed numbers. Independent LCR16a expansion in among Asian apes (box 1) includes two major clades corresponding to chromosome 13 (pink) and chromosome 16 (red) LCR16a duplications. Among African apes, three additional clades correspond to expansions of *NPIPA* (box 2) and two separate expansions of *NPIPB* (box 3). The LCR16a 'core element' is shared amongst all duplication blocks (red).

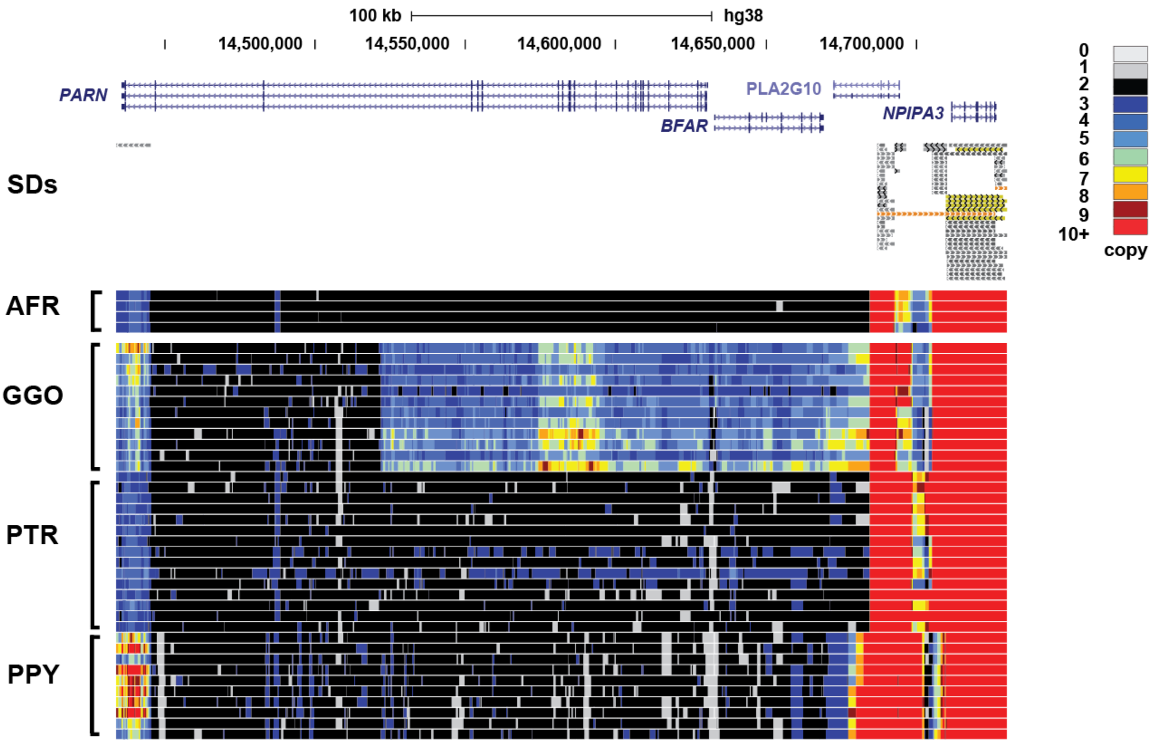

Figure S7: Lineage-specific duplicate genes. Pictured are UCSC Genome Browser snapshots (human build GRCh38) of three genes *PARN*, *BFAR*, and *PLA2G10*. Copy number (CN) heatmaps, with CN index shown, produced from Illumina read-depth predictions representative of modern humans from the Human Genome Diversity Project (HGDP) cohort, and nonhuman primates (NHPs). A large 130 kbp lineage-specific duplication of *PARN*, *BFAR*, and *PLA2G10* is identified in the gorilla, which is flanked by a copy of LCR16a.

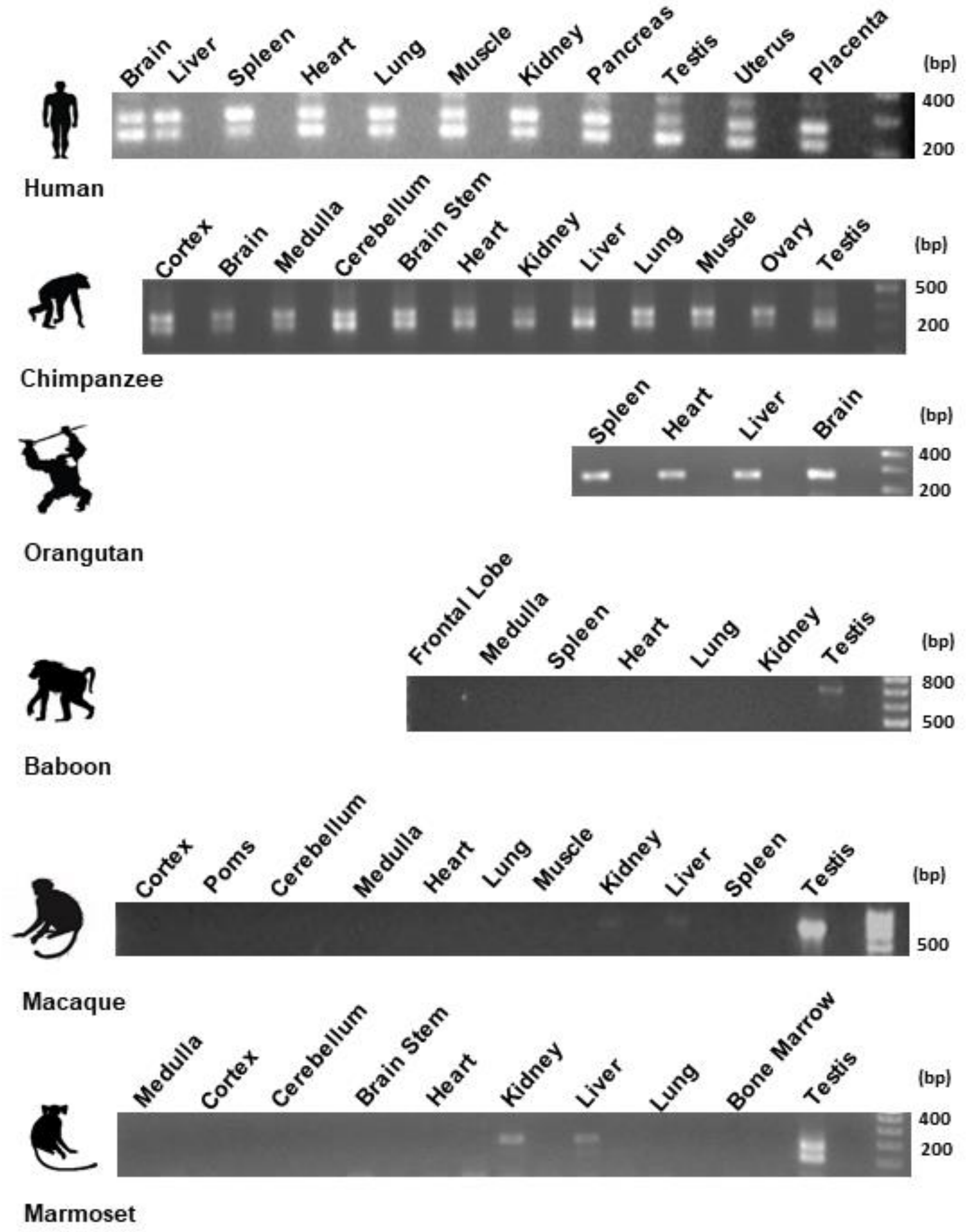

Figure S8: *NPIP* expression in a diversity panel of tissues/subtissues originating from human and NHP primary source material. RT-PCR reactions were performed using primers (Additional file 2: Table S7) designed to the canonical *NPIP* transcript described previously [13]. cDNA was prepared from mRNA generated from tissue source material (Methods) originating from chimpanzee, orangutan, baboon, macaque, and marmoset. Reactions were visualized on a 2% agarose gel, with primers designed to the ubiquitously expressed gene *UBE1* used as a control. A pattern of ubiquitous expression is observed in all great apes, while tissue-specific expression, largely limited to the testis, is observed in NWM and OWM.

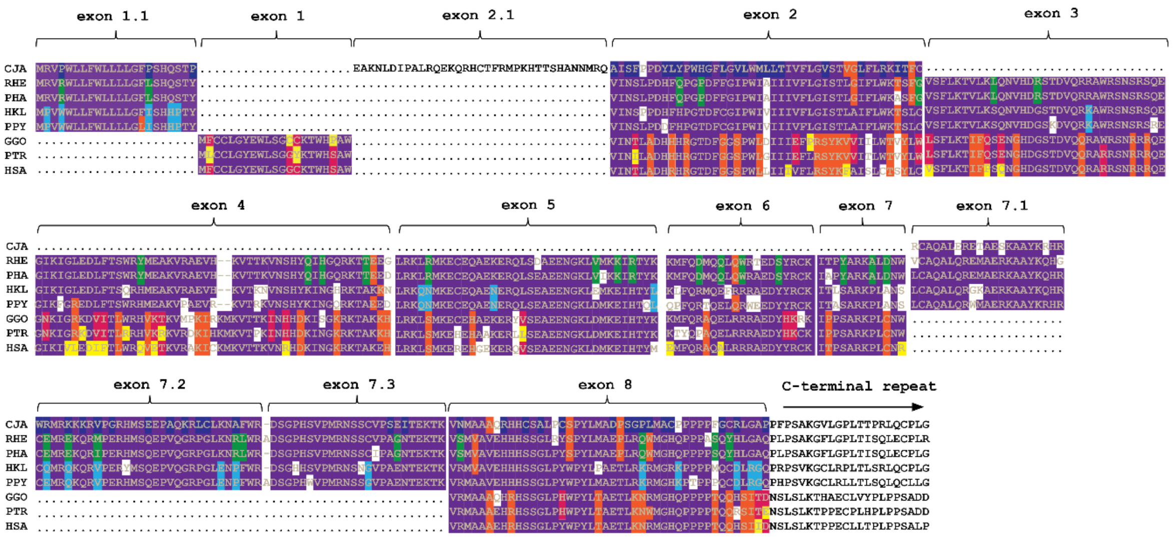

Figure S9: Gene amino acid structure of *NPIP* isoforms throughout primate evolution. A multiple sequence alignment compares *NPIP* isoforms across eight primate lineages. Individual exons are translated based on the putative open reading frames (ORFs) generated from gene models predicted using PacBio Iso-Seq and RefSeq gene annotations. Purple shading shows conserved amino acids. Marked changes at the N- and C-termini are shown during primate evolution.

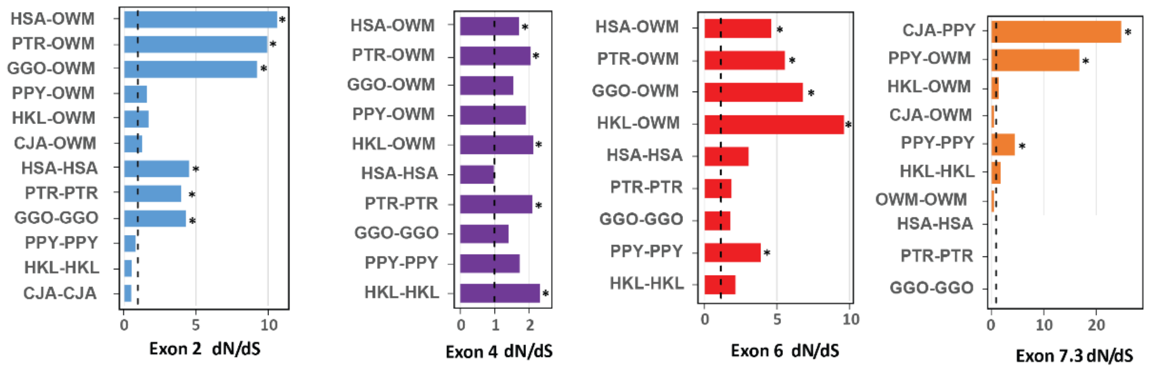

Figure S10: Selection analysis across four *NPIP*-coding exons based on an excess of nonsynonymous amino acid replacements. Graphs represent the ratio of dN/dS from individual exons between species. For each exon (exons 1-7.3 based on analysis of different primate lineages), we compared the number of nonsynonymous substitutions per site (dN) to the number of synonymous substitutions per site (dS) (Additional file 2: Table S5). Within and between species, comparisons were made (HSA=human, PTR=chimpanzee, GGO=gorilla, PPY=orangutan, HKL=gibbon, OWM=Old World monkey-baboon and macaque). Among OWM species a single copy of *NPIP* exists and thus all comparisons are orthologous. dN/dS ratios significantly greater than 1.0 are taken as evidence of positive selection (Z test transformation) (asterisks). Exons 2 and 6 show the most extreme levels of positive selection. Specifically, in exon 2 we find selection restricted to the African ape lineage (blue graph); in comparison, positive selection is observed in exon 6 (red graph) and exon 4 (blue graph) among all great apes. Note, selection is restricted to the orangutan lineage in exon 7.3 (orange graph) indicating species-specific differences for *NPIP* exons.

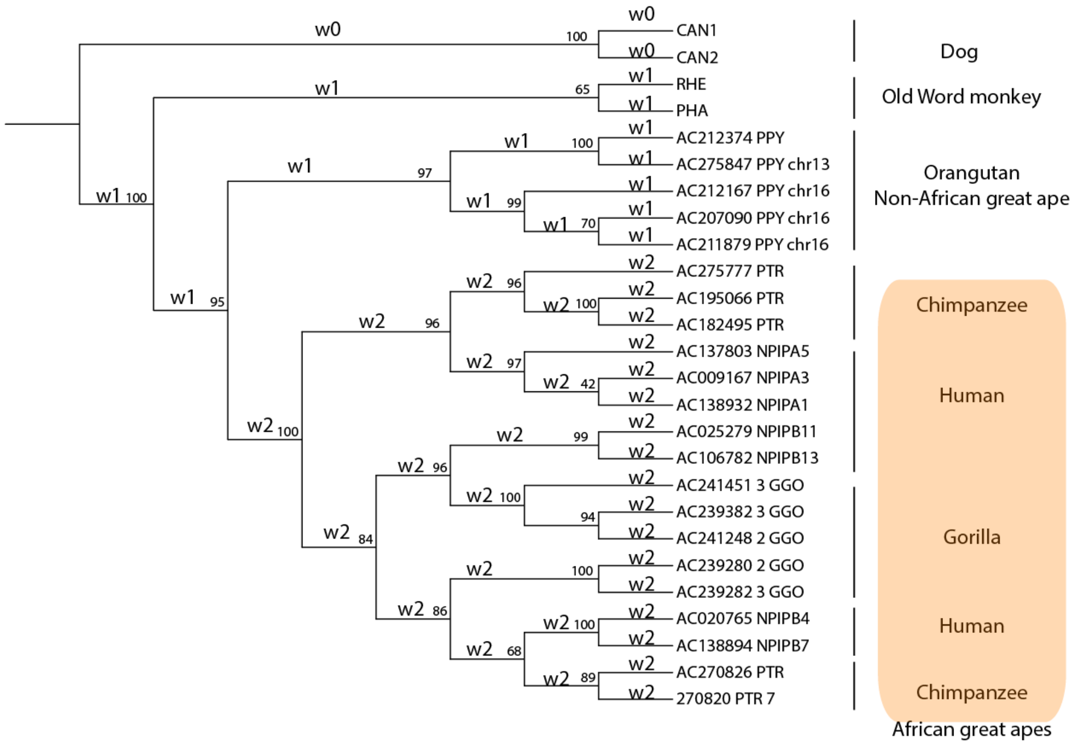

H0: w0=w1=w2=1; (netural evolution on all lineages?)
lnL= -3718.735169 (#parameters=51)

H1: w1=w2=1; w0 free; (Selection on the dog lineages?)
lnL=-3718.000394(#parameters=52); w0=0.7828; H0 vs H1: p_value= 0.2254

H2: w0=1; w1=w2 free; (Selection on the primate lineages?)
lnL= -3709.921393(#parameters=52); w2=1.7961; H0 vs H2: p_value= 2.6867e-5

H3: w0=w1=1; w2 free; (Selection on the African great ape lineages?)
lnL= -3710.773721(#parameters=52); w2=1.96412; H0 vs H3: p_value=6.5975e-5

H4: w0=w2=1; w1 free; (Selection on the orangutan and Old World monkey lineages?)
lnL= -3717.525219(#parameters=52); w1=1.46481; H0 vs H4: p_value= 0.1198

Figure S11: Evidence for positive selection in the African ape lineages using branch model analysis (PAML). The gene tree was reconstructed using a maximum likelihood method (IQ-TREE), from sequences representing dog (n=2), macaque (n=1), baboon (n=1), orangutan (n=5), gorilla (n=5), chimpanzee (n=5), and human (n=7). The *w's* in black and red represent fixed and free parameters, respectively, in the PAML analysis. Numbers below branches are the percentage of bootstrap supports for the inferred gene tree. P values are computed using the likelihood ratio test.

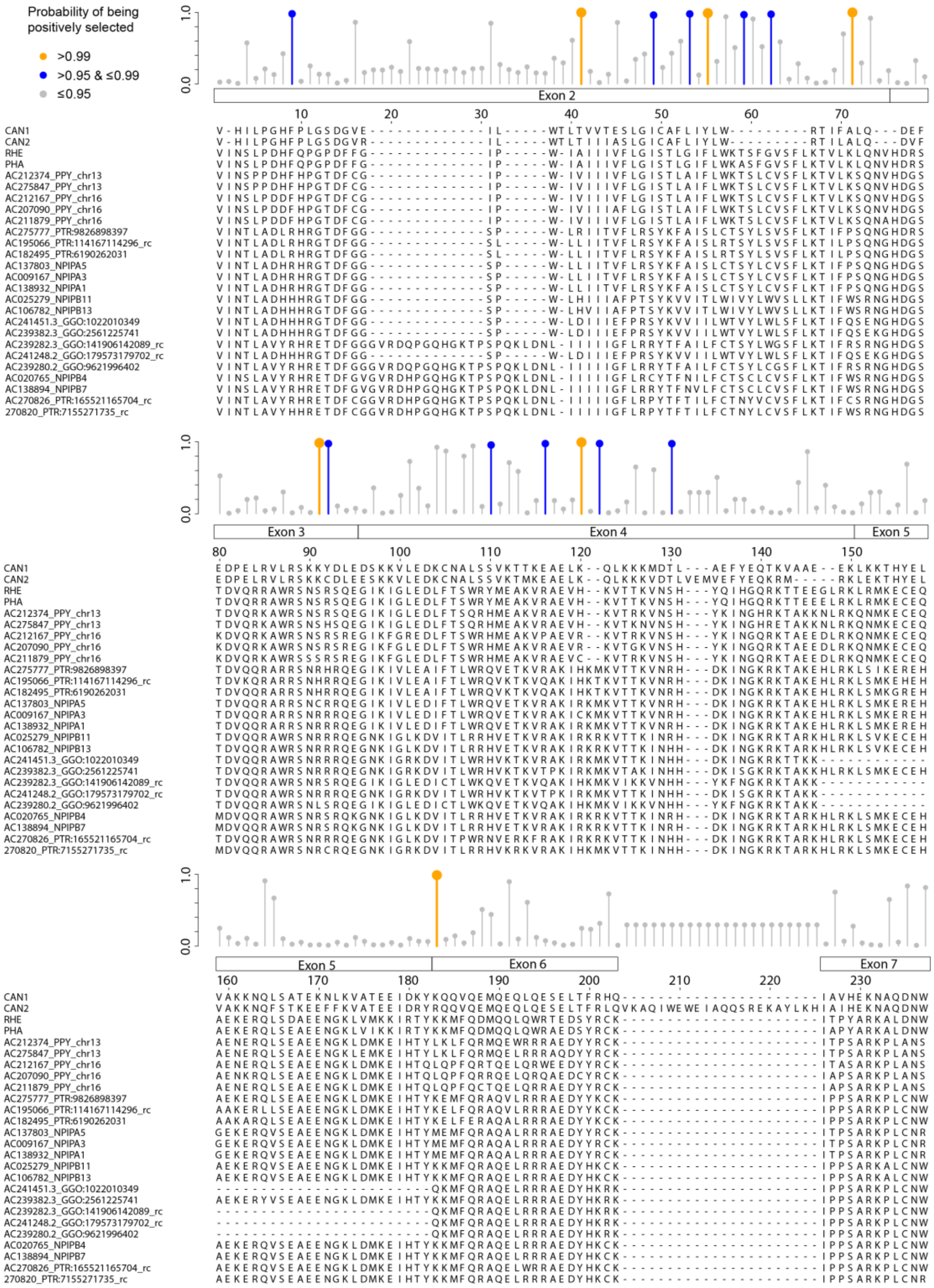

Figure S12: Evidence for positively selected amino acid sites within the African ape lineage. The branch-site test of positive selection (PAML v14.9) identifies 16 positively selected sites in exon 2, 3, 4, and 6, (8, 2, 5, and 1 sites in exon 2, 3, 4, and 6, respectively) using 26 *NPIP* sequences (dog (n=2), macaque (n=1), baboon (n=1), orangutan (n=5), gorilla (n=5), chimpanzee (n=5), and human (n=7)).

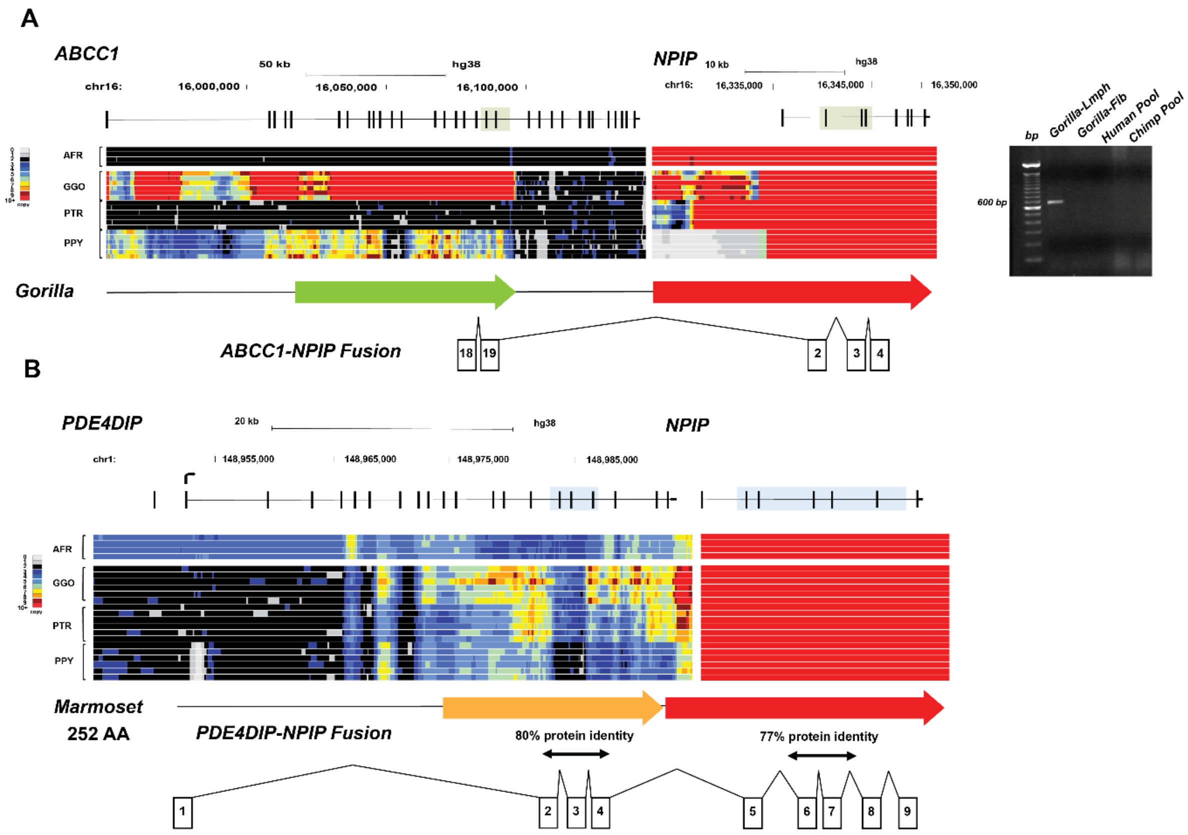

Figure S13: ***NPIP*** **gene fusion transcripts detected using PacBio Iso-Seq and RT-PCR. A)** An *ABCC1-NPIP* fusion transcript detected in gorilla. A schematic of an LCR16a duplication block on chr16p13.11 (UCSC Genome Browser snapshot). Annotation tracks include RefSeq gene annotation and copy number (CN) heatmaps (CN index shown) produced from Illumina read-depth profiles from modern humans and NHPs. Lineage-specific duplications are observed in *ABCC1* for gorilla and orangutan. BAC sequence analysis shows that the *ABCC1* duplication sits adjacent to a copy of LCR16a in gorilla (green and red arrows). An *ABCC1-NPIP* fusion transcript (exons 18 and 19 of *ABCC1* and exons 2-4 of *NPIP*) is detected from sequenced RT-PCR products originating from cDNA generated from gorilla lymphoblast source material. The corresponding product is missing from pooled cDNA (human and chimpanzee source tissue). **B)** A *PDE4DIP-NPIP* fusion transcript is detected from PacBio Iso-Seq experiments. A 252 amino acid putative ORF is predicted. The transcript originates from a lineage-specific duplication of *PDE4DIP* and LCR16a (orange and red arrows) in marmoset, creating a fusion transcript that includes exons 15-17 of *PDE4DIP* and exons 3-6 of *NPIP-S*.

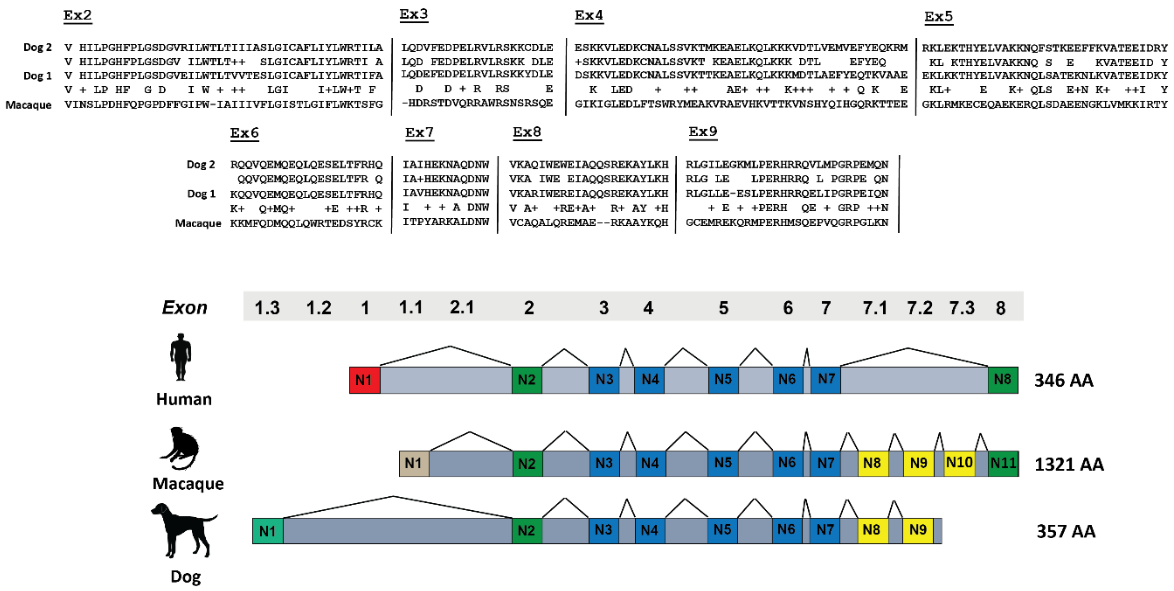

Figure S14: NPIP protein alignment and exon comparison between dog, macaque, and human. The *NPIP* ancestral gene structure is largely conserved between orthologs. Translation of putative protein-coding exons between macaque and dog *NPIP* copies identifies 8/11 exons shared between the two gene models (note three predicted canine-specific exons not shown).

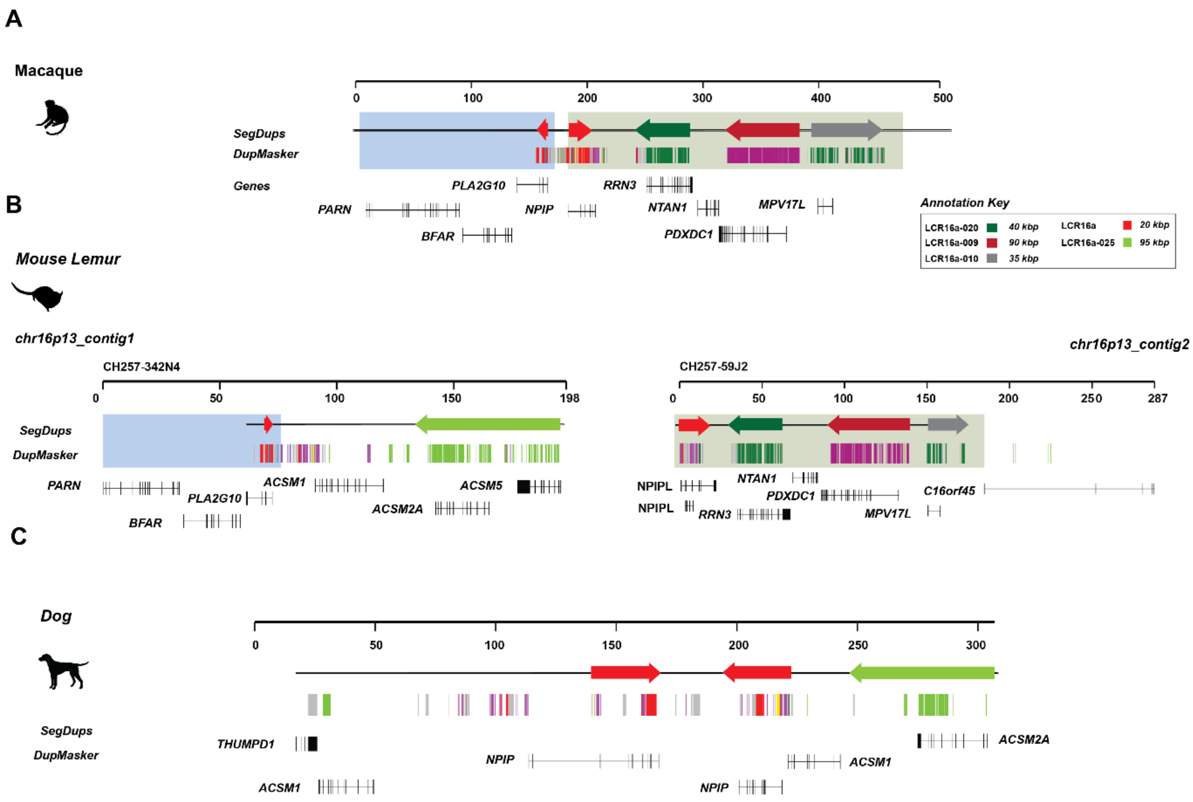

Figure S15: Cross-species comparison of the predicted ancestral *NPIP* organization between macaque, mouse lemur, and dog. Annotations include SDs (coloured arrows), gene models (black arrows), and DupMasker [50]. **A)** Large-insert clone-based assembly of the macaque locus demonstrates three ancestral duplicons, plus LCR16a (red arrow) representing a single copy of *NPIP*. **B)** Contigs mapping to the ancestral locus in the mouse lemur show that the organization is largely conserved (blue and cream shading) with the exception of a lineage-specific duplication, which includes *ACSM2A* and *ACSM5* (green arrow). **C)** Analysis of the canFAM3.0 reference assembly (chr6:24589985-24896899) identifies two unannotated copies of *NPIP* organized in an inverted orientation. Similar to the mouse lemur, an *ACSM2A* duplication is identified adjacent to *NPIP*.

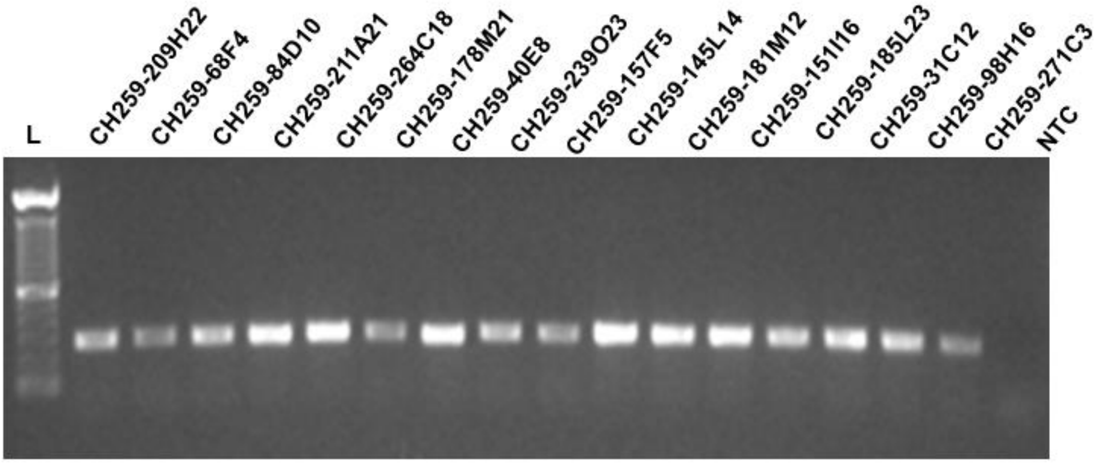

Figure S16: PCR-based testing of LCR16a-positive BAC clones derived from the CH259 large-insert clone library. DNA extracted from 16 LCR16a-positive BAC clones by hybridization were PCR confirmed for the presence of the LCR16a core duplicon. A ~300 bp product was visualized on a 2% agarose gel.

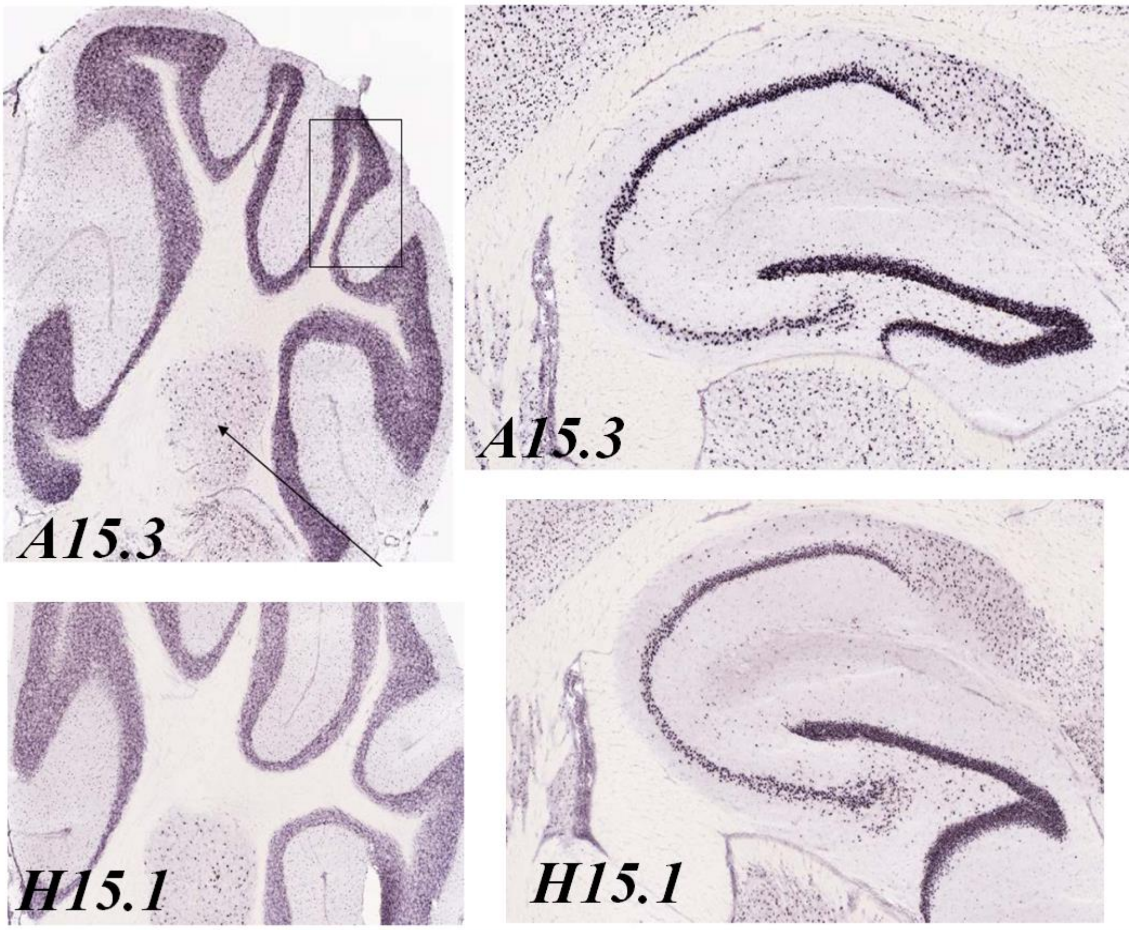

Figure S17: ISH expression analysis for BAC transgenic mice. Patterns of neuronal expression are consistent between BAC transgenic lines A15.3 and H15.1 in the hippocampus (left) and dentate gyrus (right). A15.3 and H15.1 correspond to different *NPIP* copies (RP11-344H15 and RP11-1236O14).

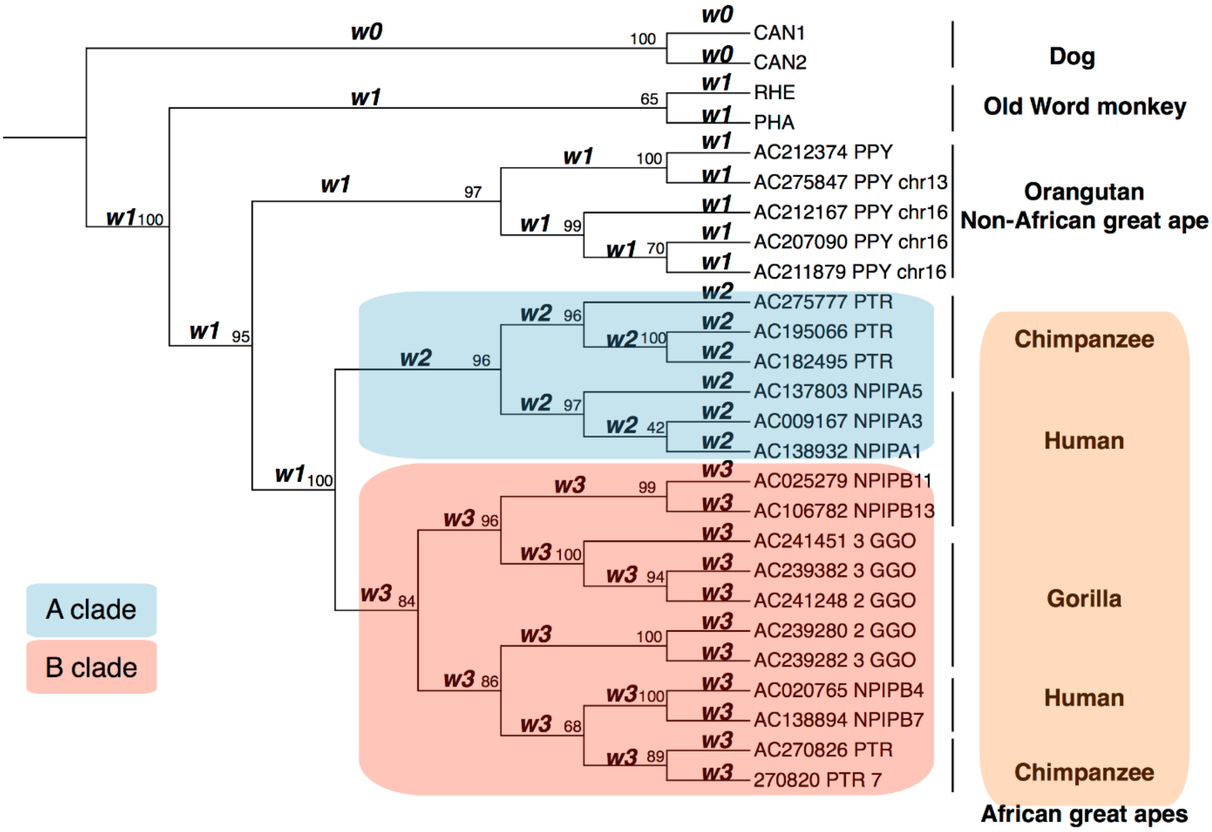

H0: *w*0=*w*1=*w*2=*w*3=1; (netural evolution on all lineages?)
lnL= -3718.735169 (#parameters=51)

H5_A clade free: *w*0=*w*1=*w*3=1; *w*2 free; (Selection on the A clade of Afircan great apes?)
lnL=-3717.877165(#parameters=52); *w*2=1.57116; H0 vs H5_A clade: p_value= 0.1902

H5_B clade free: *w*0=*w*1=*w*2=1; *w*3 free; (Selection on the B clade of Afircan great apes?)
lnL=-3712.902393(#parameters=52); *w*3=2.16776; H0 vs H5_B clade: p_value= 0.0006

Figure S18: Evidence for positive selection in the *NPIPB* subtype using branch model analysis (PAML). The gene tree was reconstructed using a maximum likelihood method (IQ-TREE). The *w's* in black and red represent fixed and free parameters, respectively, in the PAML analysis. Numbers below branches are the percentage of bootstrap supports for the inferred gene tree. P values are computed using the likelihood ratio test.

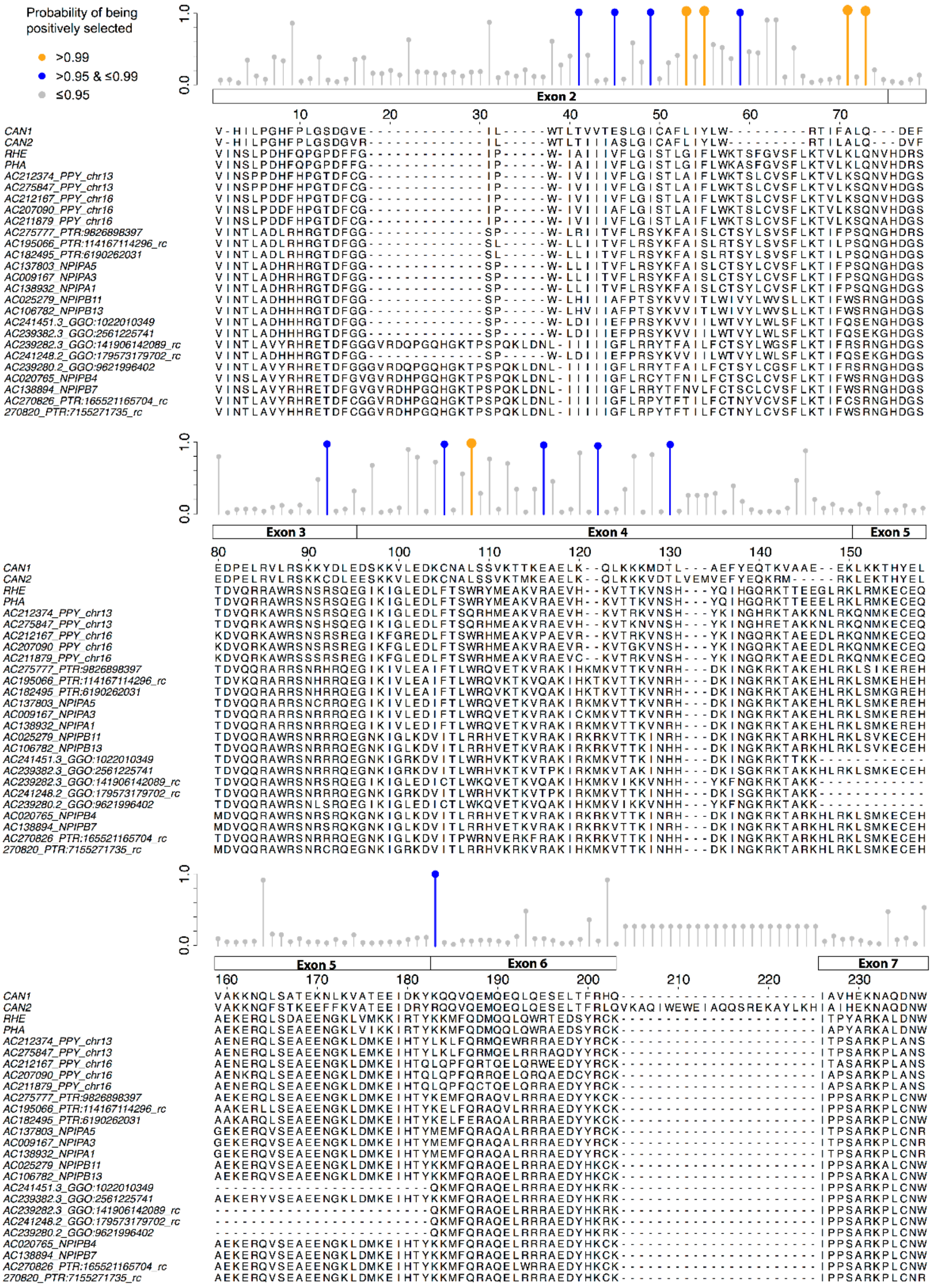

Figure S19: Evidence for positive selection relating to the *NPIPB* subtype within the African ape lineage. The branch-site test of positive selection (PAML v14.9) identifies 15 positively selected sites in exons 2, 3, 4, and 6, using 26 *NPIP* sequences (dog (n=2), macaque (n=1), baboon (n=1), orangutan (n=5), gorilla (n=5), chimpanzee (n=5), and human (n=7)).
