# Supplementary material for: An evolutionary driver of interspersed segmental duplications in primates
Source: Genome Biol. 2020 Aug 10;21:202. doi: 10.1186/s13059-020-02074-4 (PMC7419210; doi:10.1186/s13059-020-02074-4)
Supplement: Supplementary file 3 — Additional file 3. Review history. [file 13059_2020_2074_MOESM3_ESM.docx]

**Review History**

**First round of review**

**Reviewer 1**

A major, unsolved mystery of primate genomes is the organization of duplicated sequences into an interspersed architecture, as opposed to the tandem or inverted duplication architecture that typifies the genomes of other mammals. Here, Cantsilieris et al. report on the evolution and function of one sequence - LCR16a - and its potential role as an active driver of interspersed segmental duplications. The authors show that the LCR16a locus has undergone independent bouts of expansion along different primate lineages, leading to species-specific SD complexes flanking the LCR16a core duplicon. LCR16a encodes a rapidly evolving gene, NPIP, which has experienced considerable intron-exon restructuring during primate evolution and wholesale changes in gene expression. The authors also summarize a series of BAC transgenic experiments, wherein they surveyed patterns of expression of randomly integrated human and baboon BAC clones that span the LCR16a duplicon into the mouse genome.

Overall, this paper describes an impressive body of work, is well-organized, and exciting to read. The authors leverage state-of-the-art sequencing methods and genomic tools to uncover intriguing patterns of evolution at a locus that was previously inaccessible due to its duplicated architecture. Like any good study, their analysis of the LCR16a core duplicon opens up far more questions than it answers: What is the mechanism for LCR16a duplication? What is the biological function of NPIP? Why has this locus expanded in some primate lineages, but not others? However, despite these notable strengths, I do have a few major comments. I enumerate these larger criticisms below and follow up with a series of more minor points.

MAJOR COMMENTS:

I have a few concerns related to the conclusion of adaptive human-lineage specific evolution of LCR16a (page 9). First, there are insufficient details provided to fully decipher how the PAML analyses were conducted. Notably, what model of molecular evolution is assumed? How is the input tree calculated (e.g., with allowance for rate heterogeneity, invoking which model of evolution)? Second, a key assumption of the branch test is that the rate of molecular evolution proceeds in a clock-like fashion across all lineages. This assumption is violated for duplicated sequences that exchange variants via interlocus gene conversion. If the rate of gene conversion differs between species (as is almost certainly the case given that they differ in the number of LCR16a copies), this phenomenon can potentially lead to false positive signals of lineage-specific shifts in the rate of evolution. More generally, the potentially confounding effects of interlocus gene conversion (on the inference of tree topologies and rates of evolution) are not given explicit mention anywhere in the manuscript. Third, the conclusion that "our results support a major burst of positive selection specifically in the African ape lineage" does not clearly follow from the analyses presented. The branch tests the authors carry out only include human NPIP sequences, to the exclusion of sequences from non-human great apes, and cannot, therefore, be generalized beyond humans.

Second, I found the BAC transgenic experiments difficult to interpret. In particular, the random integration of clones into unknown loci in the genome and use of a hybrid (F2) genetic background make it challenging to conclude whether differences in expression are due to differences in (1) the chromatin environment of various integration sites, (2) differences in the number of BAC clones that inserted into the genome, (3) segregating modifiers that modulate expression in the genetic background, or (4) species-specific expression differences, as the authors assert. I think that an explicit mention of these caveats is warranted. In addition, data from biological replicates are not provided (either in the main text or supplement), raising questions about how robust the gene expression profiles are across replicates. Furthermore, as far as I can tell, there are no control animals used in this experiment. Do the NPIP riboprobes light up any regions in non-transgenic, control mice? Additionally, how were the riboprobes designed? If they were designed from human NPIP sequences, it may not be surprising that hybridization signals are attenuated for the baboon BAC transgenic.

Finally, although the discussion does a wonderful job of summarizing the key results in this paper, I found myself wishing that the authors had done more to contextualize their findings within the space of current literature, speculate on the mechanisms of LCR16a duplication, and suggest new steps forward in this research program. I recognize the subjective nature of this comment, but would like to encourage the authors to expand the Discussion beyond a recap of major findings. For example, the enrichment of SINEs, especially Alu elements, at LCR16a donor and acceptor sequences implicates Alus as likely agents of LCR16a duplication. Although there is some allusion to this point, it strikes me that a broader discussion of this idea is justified, particularly given the prominence of Alus in primate genome evolution. Second, the authors present possible evidence for adaptive evolution of NPIP, and the Discussion could provide a forum for speculating on the agents driving its adaptive evolution. What do we know about the function of this gene? Why might it be a target of evolution in great apes, to the apparent exclusion of other mammals?

MINOR COMMENTS:

Page 3, line15: Possible typo: extraneous "with" following "juxtaposing".

Page 4, first paragraph of results, and Table 1: I found the numbers of positively hybridizing clones difficult to interpret without information about the coverage of each library. While this information is given in the Methods, it would help the reader (or at least this one!) to mention in the main text that all libraries have ~4x coverage and/or add a column with library coverage to Table 1.

Page 7, first two paragraphs: I had a really difficult time lining up this text with Figure 4a. For example, while it is stated that "The macaque and chimpanzee haplotypes… [contain] the four ancestral duplicons…", only 2 duplicons are shown in the cartoon representation of the macaque haplotype. The 286kb region in chimpanzee referenced on line 5 is not demarcated on the figure. Given the lack of precise scaling, I'm not sure whether this region corresponds to the WSSD track harboring the LCR16a dups in inverted or tandem orientation. Similarly, the 200kb segment resulting in incomplete duplication of TEKT5 and RMI2 in marmoset is difficult to identify. Also, RMI2 is not pictured in Figure 4a.  

Page 8, first paragraph, first sentence: it is not clear what tissue material was used for the isolation of cDNA sequences.

Page 9, line 17: Clarify that the selection model is significantly better fit to the data than a model of neutral evolution.

Page 16, BAC transgenic: Have these mouse transgenic models been submitted to a public repository?

Figure S10: The legend refers to Ka/Ks ratios, but the plots show dN/dS ratios. Also, the first sentence of the legend states that "Graphs [represent] positive selection…". It would be more accurate to state "Graphs represent the ratio of dN/dS".

**Reviewer 2**

In this study (GBIO-D-19-01891) the authors examined the evolution of LCR16a, a core driver for many segmental duplications (SD), in primate lineages. Building upon a large body of previous work from the group, the authors showed a novel expansion of LCR16-assoicated SDs in the marmoset genome, in addition to the previous known expansions in great apes. In addition to the genomic analysis, the authors also demonstrated the expression pattern of LCR16-assoicated SDs in different primate tissues, as well as in mouse models with primate BAC inserts.

Overall, this manuscript presents a well-organized study. The written is clear and most of the datasets and analyses were described sufficiently. The results are important for understanding the primate genome evolution and will be of interest to readers of Genome Biology.

I do have a few minor comments:
*     Page 6 "Additionally, we mapped several loci from the grey mouse lemur and squirrel monkey corresponding to the ancestral location at chr16p13.1 …". Please provide the mapping information in the two species, may be as a part of Table S3.
*     Page 7 "Sequence Properties of LCR16a donors and acceptors": Please provide more description of the value "E" in table 3. I assume it's a ratio (i.e., enrichment) between the target regions and the random genomic regions, but it is unclear based on the description. Also more information should be provided for the simulations of the null distribution. How long are the randomly sampled regions? "SINE" and "LINE" indicate the number of elements or the length of the elements?
*     The authors used the term "African great apes" and "African apes" in the manuscript, although in some places I think they meant "African great apes and human" (e.g. in the conclusions part of the abstract). Human is a part of the great ape lineage or African ape lineage, but not African apes.
*     Table S5, the table showed the results from both BAC clones and draft assembly. Little description is given for the draft genome assemblies. Please explain why results are shown from both datasets. Please provide brief descriptions for the draft assemblies, including the download locations and version numbers.
*     There are some discrepancies between the text and figures/tables. In addition, some descriptions in the figure/table legends mismatch the figures/tables. I provide a few examples below, but this is not an exhaustive list. The authors should double-check all the legends to ensure the consistency.
o     The number of clones indicated in Table 1 is different from the clones listed in Table S1 and S2. Please clarify the difference.
o     Table 2: The first line is listed as "Primates/Prosimian". Prosiman is a part of the primate lineage. Please revise or clarify.
o     Table S3: Please explain the empty cells in the "point one" and "point two" columns.
o     Supp Fig S4A, the species labels (e.g. AFR) are not defined.
o     Supp Fig 6: The legend lists "human = HSA" but there is no HSA label in the tree.
o     Supp Fig S11: some gene labels are missing (e.g., CAN1, CAN2).

**Authors’ response to reviewers**

Reviewer reports:

Reviewer #1: A major, unsolved mystery of primate genomes is the organization of duplicated sequences into an interspersed architecture, as opposed to the tandem or inverted duplication architecture that typifies the genomes of other mammals. Here, Cantsilieris et al. report on the evolution and function of one sequence - LCR16a - and its potential role as an active driver of interspersed segmental duplications. The authors show that the LCR16a locus has undergone independent bouts of expansion along different primate lineages, leading to species-specific SD complexes flanking the LCR16a core duplicon. LCR16a encodes a rapidly evolving gene, NPIP, which has experienced considerable intron-exon restructuring during primate evolution and wholesale changes in gene expression. The authors also summarize a series of BAC transgenic experiments, wherein they surveyed patterns of expression of randomly integrated human and baboon BAC clones that span the LCR16a duplicon into the mouse genome.

Overall, this paper describes an impressive body of work, is well-organized, and exciting to read. The authors leverage state-of-the-art sequencing methods and genomic tools to uncover intriguing patterns of evolution at a locus that was previously inaccessible due to its duplicated architecture. Like any good study, their analysis of the LCR16a core duplicon opens up far more questions than it answers: What is the mechanism for LCR16a duplication? What is the biological function of NPIP? Why has this locus expanded in some primate lineages, but not others? However, despite these notable strengths, I do have a few major comments. I enumerate these larger criticisms below and follow up with a series of more minor points.

MAJOR COMMENTS:

1) I have a few concerns related to the conclusion of adaptive human-lineage specific evolution of LCR16a (page 9).

First, there are insufficient details provided to fully decipher how the PAML analyses were conducted.

Notably, what model of molecular evolution is assumed?

*We specifically tested several codon substitution models implemented in PAML assuming a model of neutral evolution from the outset and have added more details regarding precisely how the PAML analyses were performed:

Maximum likelihood analysis of positive selection for NPIP using PAML
We performed a maximum likelihood analysis using the branch models of PAML [19] and the entire gene model with the exclusion of exons 1 and 8, which are highly variable among the NPIP gene copies from dog (n=2), macaque (n=1), baboon (n=1), orangutan (n=5), gorilla (n=5), chimpanzee (n=5), and human (n=7) (Figure S11-S12). Note that we excluded the marmoset paralogs in this analysis due to the dramatic restructuring of the New World gene model. The input phylogeny is inferred using these 26 NPIP paralogs and the maximum likelihood-based method in IQ-TREE [48].

To search for evidence for selection in the dog lineage, we first constructed a null model under neutrality (H0) by setting w=1 for all branches on the gene tree (Figure S11). To test if selection occurred on the dog lineage, we set w=1 for all branches except the dog lineages (w0) (H1). The two models are not significantly different (H0 vs. H1, likelihood ratio test (LRT), p=0.2254, d.f.=1), suggesting that the dog NPIP copies likely evolved neutrally.

Next, we considered the primate lineages. We first built a model (H2) to infer selection on all ape lineages (a single free parameter w:= w1=w2, and set w0=1; Figure S11). Our data show that the H2 model is a significantly better fit than H0 (p=2.68e-5, LRT, d.f.=1), and w2 is greater than 1 (w2=1.796), suggesting positive selection acting within the primate clade. To further test if selection specifically acts on the African ape branches, we considered two additional models: selection on the African ape sequences (H3: a free parameter w2, and set w0=w1=1) or only within the Old World monkey and orangutan (H4: a free parameter w1, and set w0=w2=1). We found that while the model H3 shows evidence for positive selection on the African ape branches (p=6.59e-5, LRT, d.f.=1), the estimated w1 on the Old World monkey and orangutan branches in H4 is not significantly different from 1 (H4, p=0.1198, LRT, d.f.=1) (Figure S11). Thus, our results are consistent with the major bouts of positive selection occurring in the African ape lineages.

To identify sites that are likely the targets of positive selection, we applied the branch-site test of positive selection implemented in PAML (model=2, NSsites=2, fix_omega=0) to the same 26 sequences from exons 2 to 7 with the same configuration in the model H3. We identified 16 positively selected sites (8, 2, 5, and 1 sites in exons 2, 3, 4, and 6, respectively) in exons 2, 3, 4, and 6 (Figure S12).*


How is the input tree calculated (e.g., with allowance for rate heterogeneity, invoking which model of evolution)?

*The input for inferring the gene tree is based on the NPIP gene copies from dog (n=2), macaque (n=1), baboon (n=1), orangutan (n=5), gorilla (n=5), chimpanzee (n=5), and human (n=7) (Figure S12). Note that we excluded exons 1 and 8, which are highly variable among sequences, and the marmoset paralogs in this analysis due to the dramatic restructuring of the New World gene model. The input phylogeny is inferred using these 26 NPIP paralogs and the maximum likelihood-based method in IQ-TREE. IQ-TREE uses ModelFinder (Kalyaanamoorthy et al. 2017 Nat. Methods) to determine the best-fit model among 286 combinations of a variety of models for base substitution, codon frequencies, and rate heterogeneity across sites [48]. The best-fit model in this analysis is GTR+F+R2.*

2) A key assumption of the branch test is that the rate of molecular evolution proceeds in a clock-like fashion across all lineages. This assumption is violated for duplicated sequences that exchange variants via interlocus gene conversion. If the rate of gene conversion differs between species (as is almost certainly the case given that they differ in the number of LCR16a copies), this phenomenon can potentially lead to false positive signals of lineage-specific shifts in the rate of evolution. More generally, the potentially confounding effects of interlocus gene conversion (on the inference of tree topologies and rates of evolution) are not given explicit mention anywhere in the manuscript.

*The referee is correct that duplicated regions, especially high-identify segmental duplication regions such as NPIP, are notorious for hotspots of gene conversion (see Nuttle et al, Nature, 2015; Dumont et al, PLOS One 2013; Jackson et al, AJHG, 2005; etc.). We are aware of this limitation and have taken steps to safeguard against it. First, the average length of such interlocus gene conversions is relatively short (1.3 kbp) (Dumont et al, 2013) and we have constructed phylogenetic trees based on a minimum of 7.5 kbp in NWM and 12.4 kbp of sequence in other primates. These alignments are derived from multiple intronic sequences across 20 kbp to mitigate such effects. Second, we use OWM as an outgroup because most members of this species have only a single locus [13,14] mapping to the same position across species. Therefore all genomic loci are, effectively, orthologous to this ancestral locus (including converted loci) and we set its divergence from the human–ape branch at ~25 mya and ~35 mya for OWM–NWM separation [17]. While an independent expansion has occurred in NWM, using this timeframe, we compute ~25 mya, which is consistent with fact that 10/11 of the loci are non-orthologous to other apes and the general phylogeny of NWM. Despite these safeguards, gene conversion events may have still obfuscated some timing estimates and we have added a sentence to that effect to the main

“While we cannot completely rule out the effects of interlocus gene conversion, the fact that 10/11 marmoset loci map to non-orthologous locations when compared to other primates is consistent with a more recent dispersal of LCR16a in this lineage. In addition, it should be noted that OWM species, in general, have a single copy of LCR16 mapping to the same ancestral location[13]. In this context, all duplicate copies are orthologous to this ancestral locus irrespective of lineage-specific expansions or subsequent gene conversion events, which may confound the topology and timing estimates of the terminal branches.”*

3) The conclusion that "our results support a major burst of positive selection specifically in the African ape lineage" does not clearly follow from the analyses presented. The branch tests the authors carry out only include human NPIP sequences, to the exclusion of sequences from non-human great apes, and cannot, therefore, be generalized beyond humans.

*This is a fair point as we were partially referring to earlier work that had been performed by our lab that included an analysis of orangutan, gorilla and chimpanzee and showed positive selection restricted to the African ape lineage (Johnson et al. Nature, 2001). As part of this study, we have expanded the analysis using nearly complete gene model from dog, macaque, orangutan, gorilla, chimpanzee and human (Supplemental Fig. S11 and S12). We found no evidence for selection on the dog lineages (H1), but do find evidence of positive selection within the primate lineages (H2). Additional analyses show that the signal is restricted to the African great ape lineages (H3), but not in the orangutan and OWM lineages (H4). These findings replicate our prior analysis (Supplemental Fig. S11 and S12, Supplemental Table S5).

We have included this new analysis, referenced the older work and modified the text as follows:

“These results combined with our earlier work[13] support a major burst of positive selection specifically in the African ape lineage, while other NHPs (OWM or NWM lineages) and mammals (canids) show patterns of amino acid replacement indistinguishable from neutral evolution (Supplemental Table S5, Supplemental Fig. S11-S12 and S18-S19)”*

4) I found the BAC transgenic experiments difficult to interpret. In particular, the random integration of clones into unknown loci in the genome and use of a hybrid (F2) genetic background make it challenging to conclude whether differences in expression are due to differences in (1) the chromatin environment of various integration sites, (2) differences in the number of BAC clones that inserted into the genome, (3) segregating modifiers that modulate expression in the genetic background, or (4) species-specific expression differences, as the authors assert. I think that an explicit mention of these caveats is warranted.

*We have added details regarding the mouse transgenesis to the main text and the supplemental material. In short, we did have different founding line with different sites of integration and observed virtually identical expression patterns for human and baboon copies. Other concerns, however, are valid and we have made this clearer in the main text in the context of the potential caveats that the referee mentions:

“These observations, however, should be regarded with caution because of potential genetic background effects from the non-isogenic mice, variability in the number of copy number of BAC transgenes, and the fact that relatively few independent integrations have been studied for each construct.”

Data from biological replicates are not provided (either in the main text or supplement), raising questions about how robust the gene expression profiles are across replicates. Furthermore, as far as I can tell, there are no control animals used in this experiment.

We did have biological replicates and initially established two independent lines for each transgenic. Namely, A15.26, A15.3, O14.20, O14.23, H15.1, and H15.2 corresponding to three human BACs (AC092137, AC141267, AC142080) and one baboon BAC (AC092562) for which there were two independent lines established (I13.43, and I13.49). The initial expression results by RT-PCR were nearly identical with respect to ubiquitous expression (for all human BAC integrants) and testis specificity (baboon for both independent lines) so we proceeded with fewer mice for the in situ experiments but still included biological replicates.

We used four human BAC transgenic lines representing two independent integrations of BAC RP11-344H15 (A15.26 and A15.3) and two independent integrations of baboon BAC RP41-285I13 (I13.43 and I13.49) for the in situ experiments. It should also be noted that the three independent paralogs of the human NPIP versions each essentially gave nearly identical in situ results consistent with the observations from the primary tissue (Supplemental Fig. S17). We do note, however, there was some evidence of differences in levels of expression although no difference in the overall pattern with respect to the primary tissue and we agree that genetic background, modifiers and site of integration all are potential confounding factors. As mentioned above, we have made this clearer in the main text in the context of the potential caveats and included this information in the Supplemental Methods.*

Furthermore, as far as I can tell, there are no control animals used in this experiment. Do the NPIP riboprobes light up any regions in non-transgenic, control mice? Additionally, how were the riboprobes designed? If they were designed from human NPIP sequences, it may not be surprising that hybridization signals are attenuated for the baboon BAC transgenic.

*A control probe from the mouse locus, Drd1a, was used as a positive control for the ISH experiments and was hybridized to tissue sections from each of mouse transgenic lines. NPIP probes specific to the corresponding sequence in both baboon and human were designed based on cDNA sequence available for representative loci at that time. In the case of baboon, we specifically designed a 659 bp probe derived from cDNA (XM_001109190) from macaque, which is 99% identical. In the case of human, we selected two probes based on available human cDNA (see Supplemental Table S9). A homologous NPIP gene and/or the gene family does not appear to exist in the mouse. Sequence analysis of the corresponding homologous locus shows that it is highly divergent and lacks any significant sequence homology with the transcripts identified amongst primates. We have added these details to the main text and to the supplemental methods section of the document.

“Because the NPIP gene family has been a target of positive selection in the human lineage compared to OWM, we designed ISH probes to the homologous region for humans and OWM cDNA separately (Supplemental Methods)”*

5) Although the discussion does a wonderful job of summarizing the key results in this paper, I found myself wishing that the authors had done more to contextualize their findings within the space of current literature, speculate on the mechanisms of LCR16a duplication, and suggest new steps forward in this research program. I recognize the subjective nature of this comment, but would like to encourage the authors to expand the Discussion beyond a recap of major findings. For example, the enrichment of SINEs, especially Alu elements, at LCR16a donor and acceptor sequences implicates Alus as likely agents of LCR16a duplication. Although there is some allusion to this point, it strikes me that a broader discussion of this idea is justified, particularly given the prominence of Alus in primate genome evolution.

*We have now expanded the discussion in the context of our major findings on the association of Alu repeats and potential insights into mechanism.*

*“It is interesting in this context that the only common feature of the LCR16a acceptor and donor regions are that that they tend to be GC-rich and significantly enriched for SINE (in particular Alu) repeats (Supplemental Fig. S2 and S3). This independent association with Alu repeats in multiple primate lineages may provide some insight into mechanism of origin and propagation. Among common repeat elements, Alu repeats are known to be enriched and possibly selected for in early replicating GC-rich regions of the genome[28], they are preferential sites for structural variation and segmental duplication possibly due to homology directed repair/recombination [29, 30], and Alu-rich DNA appears to be organized in the interphase nucleus along the surface of chromatin facing the nuclear envelope[31]. These apparently unique properties of this repeat may facilitate repair and replication of non-allelic homologous segments of DNA through homology, proximity, and accessibility leading to the enrichment of Alu repeats at donor, acceptor, as well as breakpoint regions. Moreover, the fact that the primate-specific Alu repeat subfamily experienced a burst of retrotransposition 40 million years ago[32] may also explain why these complex LCR16a-associated duplications have been largely restricted to NWM and OWM species such as the apes. These observations are consistent with the broader hypothesis that the expansion of the Alu repeat mobile element sensitized primate genome to segmental duplications[29, 33].”*

Second, the authors present possible evidence for adaptive evolution of NPIP, and the Discussion could provide a forum for speculating on the agents driving its adaptive evolution. What do we know about the function of this gene? Why might it be a target of evolution in great apes, to the apparent exclusion of other mammals?

*We have also elaborated more in the final paragraph of the discussion on some of the next steps in NPIP research with a specific emphasis on what we know about the function of this gene.

“Our comparative analyses suggest that the widespread neuronal expression of this gene family occurred early in great ape evolution prior to the major bouts of exonic positive selection in the African ape lineage, but that the propensity of LCR16a to create interspersed duplications is a far more ancient property. A critical next step in this research is a determination of the molecular function and phenotypic consequence of the gain and loss of individual NPIP genes. At present there are only a few clues. Consistent with the BAC transgenic in situ experiments, NPIP has been reported to be highly expressed (eightfold) in the foveomacula when compared to the retina[37]—second in transcript abundance only after the cone opsin genes. In a separate study, analysis of the single-cell gene expression data from primate cerebral organoid tissue reveals that one member, NPIPB5, shows distinguishable levels of human-specific gene expression in excitatory neurons and radial glia cells[38]. This is particularly noteworthy because the NPIPB subfamily has expanded almost exclusively in chimpanzee, human, and gorilla and is underlying much of the African ape positive selection. There is also evidence that the gene family continues to experience rapid positive selection specifically in the human lineage. For example, we recently identified a previously unknown member of the NPIPB family, NPIPB16, mapping to a 383 kbp Melanesian-specific duplication that originated ~400 kya in a Denisova hominin [39]. Despite its recent origin, NPIPB16 is expressed, maintains an ORF, and shows strong signals of positive selection (~3% amino acid divergence) consistent with other gene paralogs we report here. Remarkably, the 383 kbp segment of DNA that introgressed into the Melanesian ancestral populations ~40,000 kya has now risen to high frequency (>79%) among modern-day Papuan populations likely as a result of partial selective sweep. These findings suggest that NPIP continues to not only restructure hominin genomes but also rapidly adapt and evolve possibly in response to a pressure or agent still challenging the human and African great ape species more broadly”*


MINOR COMMENTS:

1) Page 3, line15: Possible typo: extraneous "with" following "juxtaposing".

*This has been corrected and now reads as “In most cases they are found juxtaposing with other SDs of diverse evolutionary origin”*

2) Page 4, first paragraph of results, and Table 1: I found the numbers of positively hybridizing clones difficult to interpret without information about the coverage of each library. While this information is given in the Methods, it would help the reader (or at least this one!) to mention in the main text that all libraries have ~4x coverage and/or add a column with library coverage to Table 1.

*A column has been included in Table 1 demonstrating the individual coverage estimate for each library where possible. We have also included an average coverage estimate in the main text: “one non-ape species, marmoset, stood out with 110 positively hybridizing clones suggesting an expansion of >15 copies in that NWM lineage (average library coverage 6-7x).”*

3) Page 7, first two paragraphs: I had a really difficult time lining up this text with Figure 4a. For example, while it is stated that "The macaque and chimpanzee haplotypes… [contain] the four ancestral duplicons…", only 2 duplicons are shown in the cartoon representation of the macaque haplotype.

*We have clarified the text by adding references to the gene names and coloured arrows representing the ancestral duplicons. The sentence now reads “The macaque and chimpanzee haplotypes, for example, show the simplest organization containing four ancestral duplicons, which include the genes MPV17L, PDXDC1, RRN3 and NPIPA (green, maroon, grey and red arrows, respectively). These duplications are shared amongst all the lineages and likely represent the primate archetype (Fig. 4A).”*

4) The 286kb region in chimpanzee referenced on line 5 is not demarcated on the figure. Given the lack of precise scaling, I'm not sure whether this region corresponds to the WSSD track harboring the LCR16a dups in inverted or tandem orientation.

*We have updated the text to include specific references to Fig4A in order to increase clarity: “In chimpanzee, we identify two additional inverted copies of LCR16a, which are absent from the macaque assembly. These chimpanzee-specific LCR16a copies reside in a large ~168 kbp region of increased read depth (detected by whole-genome shotgun sequence detection [WSSD]; blue underlining bar) and add an additional 40 kbp of sequence to the locus (red shading) (Fig.4A).”*
 *We have also updated the figure accordingly demonstrating the region we refer to with a black bar.*

5) The 200kb segment resulting in incomplete duplication of TEKT5 and RMI2 in marmoset is difficult to identify. Also, RMI2 is not pictured in Figure 4a.

*We have corrected the two errors from Figure 4A that the reviewer has highlighted. The figure has been updated to correctly include RMI2. The 200 kbp segment referred to in the text is now indicated by the black bar in the figure.*

6) Page 8, first paragraph, first sentence: it is not clear what tissue material was used for the isolation of cDNA sequences.

*A comprehensive list of primate tissues and subtissues is included in the Methods under tissue samples. We have included a reference to the Methods and Supplemental Figure S8 in this sentence.*

7) Page 9, line 17: Clarify that the selection model is significantly better fit to the data than a model of neutral evolution.

*We have clarified this in the text and it now reads “In the dog, we found that there was no significant difference between the null model under neutrality and test for positive selection (p=0.2254, LRT d.f.=1) indicating that the dog NPIP copies likely evolved neutrally. In comparison, the model under selection within the primate lineage was a significantly better fit to the data than the neutral model (p=2.6867e-5, LRT, d.f.=1).”*

8) Page 16, BAC transgenic: Have these mouse transgenic models been submitted to a public repository?
*Yes, we have deposited four representative transgenic strains as part of the Jackson laboratory Cryo recovery service (https://www.jax.org/mouse-search?searchTerm=NPIP). Frozen embryos can be recovered into viable mice within 11-14 days. Specifically, we deposited two congenic lines corresponding to the human NPIP copy corresponding to H15 (RP11-344H15) transgenic (Stock no: 012727 and 012729) and the original founder line on a mixed background (Stock no: 12728). We also included a congenic line corresponding to the baboon transgenic congenic line B6.Cg-Tg(RP41-285I13)I13.49Eich/J (Stock no :12731).*

*B6.Cg-Tg(RP11-344H15)aH15Eich/J
Cryo Recovery
Stock No: 012727
Congenic Transgenic

B6.Cg-Tg(RP11-344H15)cH15Eich/J
Cryo Recovery
Stock No: 012729
Congenic Transgenic

B6.Cg-Tg(RP41-285I13)I13.49Eich/J
Cryo Recovery
Stock No: 012731
Congenic Transgenic

B6;SJL-Tg(RP11-344H15)bH15Eich/J
Cryo Recovery
Stock No: 012728
Transgenic*

9) Figure S10: The legend refers to Ka/Ks ratios, but the plots show dN/dS ratios. Also, the first sentence of the legend states that "Graphs [represent] positive selection…". It would be more accurate to state "Graphs represent the ratio of dN/dS".

*The legend for Supplementary S10 has been updated to the following “Graphs represent the ratio of dN/dS from individual exons between species” as requested by the reviewer. We have also updated Ka/Ka ratios to dN/dS ratios in the legend and throughout Supplementary Table S5.*
 
Reviewer #2: In this study (GBIO-D-19-01891) the authors examined the evolution of LCR16a, a core driver for many segmental duplications (SD), in primate lineages. Building upon a large body of previous work from the group, the authors showed a novel expansion of LCR16-assoicated SDs in the marmoset genome, in addition to the previous known expansions in great apes. In addition to the genomic analysis, the authors also demonstrated the expression pattern of LCR16-assoicated SDs in different primate tissues, as well as in mouse models with primate BAC inserts.

Overall, this manuscript presents a well-organized study. The written is clear and most of the datasets and analyses were described sufficiently. The results are important for understanding the primate genome evolution and will be of interest to readers of Genome Biology.

MINOR COMMENTS

1) Page 6 "Additionally, we mapped several loci from the grey mouse lemur and squirrel monkey corresponding to the ancestral location at chr16p13.1 …". Please provide the mapping information in the two species, may be as a part of Table S3.

*The map location has been added to Supplemental Table S3 and a reference to this has been updated in the main text per the reviewer’s request.*

2) Page 7 "Sequence Properties of LCR16a donors and acceptors": Please provide more description of the value "E" in table 3. I assume it's a ratio (i.e., enrichment) between the target regions and the random genomic regions, but it is unclear based on the description. Also more information should be provided for the simulations of the null distribution. How long are the randomly sampled regions? "SINE" and "LINE" indicate the number of elements or the length of the elements? *

*The "E" value presented in Table 3 represents the enrichment coefficient based on 10,000 permutations. It was calculated simply based on the observed value divided by the expected where the expected values were defined as the mean of the 10,000 genome-wide permutations. Randomly selected regions were chosen to match the lengths of the donor, acceptor, and pre-integration sites. The numbers refer to the counts of LINE and SINE in the distributed segments and the E value represents their enrichment factor comparing the observed versus random expectation. We have added these additional details to the footnote of the table.*

3) The authors used the term "African great apes" and "African apes" in the manuscript, although in some places I think they meant "African great apes and human" (e.g. in the conclusions part of the abstract). Human is a part of the great ape lineage or African ape lineage, but not African apes.

*We have corrected this the conclusion now reads “Dramatic changes to NPIP gene structure and altered tissue expression preceded major bouts of positive selection in the African ape lineage suggestive of a gene undergoing strong adaptive evolution.”*

4) Table S5, the table showed the results from both BAC clones and draft assembly. Little description is given for the draft genome assemblies. Please explain why results are shown from both datasets.

*We have clarified this in the sequence, selection and duplication analysis methods of the main text:

“We extracted coding sequences from high-quality contiguous BAC sequence (~79) and from working draft assemblies primarily ordered and orientated into multiple contigs using Sanger sequencing (~72). In order to create optimal global alignments for downstream analysis, groups of finished and unfinished clones were analyzed separately.”*

5) Please provide brief descriptions for the draft assemblies, including the download locations and version numbers.

*We have also added a column to Supplementary Table S2 to designate whether the clone sequence is a finished or working draft assembly with corresponding accession numbers in NCBI.

In the main text under “Data Access” we included a statement that all clone sequencing data can be accessed under NCBI Bio Project ID: PRJNA593285.*

6) There are some discrepancies between the text and figures/tables. In addition, some descriptions in the figure/table legends mismatch the figures/tables. I provide a few examples below, but this is not an exhaustive list. The authors should double-check all the legends to ensure the consistency.
The number of clones indicated in Table 1 is different from the clones listed in Table S1 and S2. Please clarify the difference.

*The clones in Table 1 represent the total number of clones recovered from BAC hybridizations using probes specific to LCR16a. We estimated LCR16a copy number in each lineage by taking the ratio of recovered clones by the estimated clone coverage in each library. We now include the coverage estimates in Table 1 and updated this under the Library hybridization and BAC end sequencing methods.

Supplemental Tables S1 and S2 represent a subset of nonredundant clones sequenced using SMRT long-read PacBio sequencing. These were selected from the total list of recovered clones (Table 1) using two approaches: 1) BAC-end sequence placements mapped to the human genome and 2) Illumina-based short-read sequencing in the absence of BAC-end sequence data. We have clarified this in the Methods:

“In order to prioritize clones for high-quality sequence and assembly, we used repeat-masked BAC end sequence pairs that were rescored for quality and mapped against the human reference genome (GRCh38). In the absence of BAC-end sequence placements, we also sequenced a subset of BAC clones using short-read Illumina sequencing as outlined below.”*

7) Table 2: The first line is listed as "Primates/Prosimian". Prosiman is a part of the primate lineage. Please revise or clarify.

*This has been revised to include just the primate lineage in Table 2.*

8) Table S3: Please explain the empty cells in the "point one" and "point two" columns.

*The cells have been updated with the correct map locations as requested*

9) Supp Fig S4A, the species labels (e.g. AFR) are not defined.

*The species labels have now been defined in the figure legend: “The CN heatmaps include representations of AFR=African Humans, GGO=gorilla, PTR=chimpanzee, PPY=orangutan.”*

10) Supp Fig 6: The legend lists "human = HSA" but there is no HSA label in the tree.

*The species labels for HSA=human have now been added to the tree branches of Supplemental Fig. S6.*

11) Supp Fig S11: some gene labels are missing (e.g., CAN1, CAN2)

*The species labels have now been added to the tree branches of Supplemental Fig. S11.*

**Second round of review**

**Reviewer 1**

I thank the authors for their careful consideration and thorough responses to my prior comments. I have re-read the revised manuscript and have no further concerns.
